# Boosting Organic Long Persistent Luminescence by Enhancing Charge Separation Processes in Three‐Component Systems

**DOI:** 10.1002/advs.202501558

**Published:** 2025-06-30

**Authors:** Zi Ye, Wen Xia, Guangming Wang, Guoyi Wu, Hongxin Gao, Biao Xu, Kaka Zhang

**Affiliations:** ^1^ State Key Laboratory of Organometallic Chemistry and Shanghai Hongkong Joint Laboratory in Chemical Synthesis Key Laboratory of Synthetic and Self‐Assembly Chemistry for Organic Functional Molecules Ningbo Zhongke Creation Center of New Materials Shanghai Institute of Organic Chemistry University of Chinese Academy of Sciences Chinese Academy of Sciences 345 Lingling Road Shanghai 200032 P. R. China

**Keywords:** charge separation, difluoroboron β‐diketonate, organic afterglow, organic long persistent luminescence, phosphorescence

## Abstract

Organic long persistent luminescence (OLPL) materials feature power law emission decay and minutes‐/hours‐long afterglow durations because of retarded charge recombination. Unlike conventional room‐temperature phosphorescence (RTP) and thermally activated delayed fluorescence (TADF) afterglow, the emergence of OLPL must include a charge separation process in its photophysical mechanism; consequently, the reported OLPL examples are much fewer than conventional afterglow materials. The incorporation of an electron donor or acceptor is conceived to interact with the long‐lived excited state in conventional afterglow system, aiming to induce charge separation. Here, the study first builds two‐component RTP/TADF afterglow systems composed of difluoroboron β‐diketonate (BF_2_bdk) dopants and organic crystalline matrices, and then introduces an electron‐donating component into the two‐component BF_2_bdk‐matrix systems to enable the charge separation processes. The resultant three‐component materials exhibit visible‐light‐excitable OLPL afterglow lasting for several hours under ambient condition. Leveraging the efficient harvesting of singlet/triplet excitons by BF_2_bdk and the protective environment provided by the crystalline matrix, the three‐component materials exhibit an estimated OLPL efficiency of ≈10% and display OLPL brightness comparable to inorganic Sr_2_Al_14_O_25_/Eu^2+^, Dy^3+^ materials. Furthermore, the obtained OLPL materials show promising applications in afterglow displays and information storage, marking a significant step toward practical implementations of organic afterglow materials.

## Graphical abstract

Electron donor TMB is introduced into BF_2_bdk‐PhB system to enhance charge separation process. The resultant three‐component materials exhibit visible‐light‐excitable OLPL afterglow lasting for several hours under ambient environment, with OLPL efficiency of ≈10% and display OLPL brightness comparable to inorganic materials. Furthermore, the obtained OLPL materials show promising applications in afterglow displays and information storage.

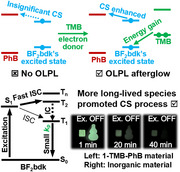

## 1 Introduction

Room‐temperature phosphorescence (RTP) and organic afterglow materials possess significant potential for applications in fields such as bioimaging, oxygen sensing, and security anti‐counterfeiting, owing to their long‐lived excited states.^[^ ^1^, ^2^, ^3^, ^4^, ^5^, ^6^, ^7^, ^8^, ^9^, ^10^, ^11^, ^12^, ^13^, ^14^, ^15^, ^16^ ^]^ Notably, organic long persistent luminescence (OLPL) materials can exhibit afterglow durations extending from several minutes to several hours upon excitation.^[^ ^17^, ^18^, ^19^, ^20^, ^21^, ^22^, ^23^, ^24^, ^25^, ^26^, ^27^, ^28^, ^29^, ^30^ ^]^ Compared to conventional inorganic long persistent luminescence materials, OLPL materials offer several advantages: lower processing temperatures (≈200 °C or less), cost‐effective raw materials that are often sustainable (free from rare earth metals), and the ability to disperse molecularly within a matrix to achieve transparency—unlike the micron‐ or submicron‐sized particles typically used in inorganic systems.^[^ ^17^, ^18^, ^19^ ^]^ Additionally, polymer‐based OLPL materials possess superior mechanical properties and can be easily integrated with conventional polymer processing techniques for scalable production.^[^ ^31^ ^]^ However, there is a limited range of OLPL materials, especially those that are comparable to inorganic materials in terms of afterglow brightness and duration. The development of high‐performance OLPL materials is thus a crucial scientific challenge, essential for their widespread application in various sectors such as home and public safety, lighting and decoration, personal accessories and outdoor gear, education and toys, emergency preparedness, and healthcare and wellness.^[^ ^17^, ^18^, ^21^, ^26^, ^31^, ^32^, ^33^, ^34^ ^]^

As early as 1997, Yamamoto, Ohkita and co‐workers discovered that *N*,*N*,*N*′,*N*′‐tetramethylbenzidine (TMB) embedded in a poly(alkyl methacrylate) matrix could undergo two‐photon ionization under strong light irradiation at 20 K, leading to the formation of charge‐separated states, followed by a retarded charge recombination and a prolonged OLPL afterglow lasting several hours.^[^ ^35^ ^]^ Subsequently, until 2017, Adachi, Kabe, and co‐workers reported the first room‐temperature OLPL sample with afterglow exceeding 1 h under nitrogen upon excitation with a standard LED white light, which was prepared as a two‐component thin film TMB:PPT (PPT represents 2,8‐bis(diphenylphosphoryl)dibenzo[b,d] thiophene) by dispersing the electron donor TMB into the electron acceptor PPT via the melt‐casting method in an inert gas atmosphere.^[^ ^17^ ^]^ In this system, upon photo‐excitation, electrons can transfer from the highest occupied molecular orbital (HOMO) of the donor to that of the excited‐state acceptor, leading to the formation of charge transfer states. Subsequently, the acceptor radical anions diffuse away, effectively separating the donor radical cations from the acceptor radical anions and resulting in the establishment of charge‐separated states. Following this, retarded charge recombination between the radical anions and radical cations occurs, generating exciplex emission (a transition from the acceptor's the lowest unoccupied molecular orbital (LUMO) to the donor's HOMO) and achieving an OLPL afterglow with hour‐long durations. Later at 2020, Sameul, Zysman‐Colman, and coworkers reported the first room‐temperature OLPL materials based on two‐photon ionization (TPI) mechanism, whose OLPL afterglow duration exceeded 1 h under oxygen‐free conditions at room temperature.^[^ ^19^ ^]^ This was accomplished by doping the molecule 3‐(4‐(9H‐carbazol‐9‐yl)phenyl)acenaphtho [1,2‐b] pyrazine‐8,9‐dicarbonitrile (CzPhAP) into organic matrices such as PPT, 2,2′,2′′‐(1,3,5‐benzinetriyl)‐tris(1phenyl‐1‐H‐benzimidazole) (TPBi), or poly(methyl methacrylate) (PMMA). Under strong light irradiation, CzPhAP molecules can be ionized by absorbing two photons in succession, and the ionized electrons can be trapped by the matrices, leading to charge separation, followed by the retarded charge recombination and the release of an hour‐long OLPL afterglow.

To date, there have been only ≈30 reported studies on OLPL afterglow materials (in striking contrast to the thousands of reports on organic RTP materials), which are mainly based on donor‐acceptor mechanisms, some on TPI mechanism, and a few on undefined mechanism (Figure S1, Supporting Information). A common characteristic of these mechanisms is their reliance on charge separation and subsequent charge recombination processes to exhibit OLPL afterglow that conforms to a power‐law decay characteristic. Differently, in TPI‐triggered OLPL systems, high excitation power is typically required to induce charge separation;^[^ ^19^, ^20^, ^22^ ^]^ whereas in OLPL systems based on donor‐acceptor mechanism, charge separation can be achieved through intermolecular charge transfer with weak or normal excitation power.^[^ ^17^, ^18^, ^31^, ^32^ ^]^ However, due to the small oscillator strength of the intermolecular charge transfer states, the efficiency of harvesting singlet/triplet excitons during charge recombination is typically low in donor‐acceptor mechanism.^[^ ^23^ ^]^ Therefore, developing a new organic system that allows charge separation by normal/weak excitation source (preferably, visible light) and can efficiently harvest singlet/triplet excitons upon recombination, is of paramount importance.

Given the extensive literature on RTP and organic afterglow materials, some of which exhibit both high afterglow quantum yields and long extended afterglow lifetimes.^[^ ^36^, ^37^, ^38^, ^39^, ^40^, ^41^ ^]^ We envisage that if a new component is introduced into these afterglow systems to interact with the long‐lived excited states of the original components, and the electron transfer between the components is achieved by a rational choice of HOMO/LUMO energy structures, it would be possible to achieve charge separation of the system by weak or normal light excitation. The rigid environment originally protecting the long‐lived excited states can also stabilize the intermediates formed post‐charge separation. Moreover, if the system contains molecules that can efficiently capture the energy released during charge recombination, there exists a promising opportunity to develop high‐performance OLPL afterglow materials.

In recent years, our group has carried out a lot of two‐component organic afterglow materials based on **BF_2_bdk**‐matrix system, synthesizing hundreds to thousands of **BF_2_bdk** molecules. **BF_2_bdk** molucules exhibit high molar absorption coefficients (*ε*) and photoluminescence quantum yields (PLQY).^[^ ^38^, ^42^, ^43^, ^44^, ^45^, ^46^, ^47^ ^]^ We have also screened over a hundred organic small molecules and polymer matrices as organic matrices for the construction of afterglow materials.^[^ ^38^, ^43^, ^44^, ^48^ ^]^ Our findings indicate that the matrix not only provides a rigid environment that protect the triplet excited states but also interacts with the S_1_ states of **BF_2_bdk** molecules, lowering their S_1_ energy level while exerting minimal influence on their T_1_ energy level, thereby reducing Δ*E*_ST_ and promoting intersystem crossing.^[^ ^38^, ^42^, ^43^, ^44^, ^45^, ^49^ ^]^ Besides, through systematic modification of the structure of **BF_2_bdk** molecules, we have elucidated that the T_n_ (n = 2 or 3) excited states in the **BF_2_bdk** system can mediate the S_1_‐T_1_ intersystem crossing, and obtained various efficient long‐lived two‐component afterglow materials.^[^ ^43^, ^45^ ^]^ However, these two‐component materials can only exhibit RTP‐type or TADF‐type afterglow but not OLPL afterglow under weak or normal light excitation at ambient conditions.

**Figure 1:**
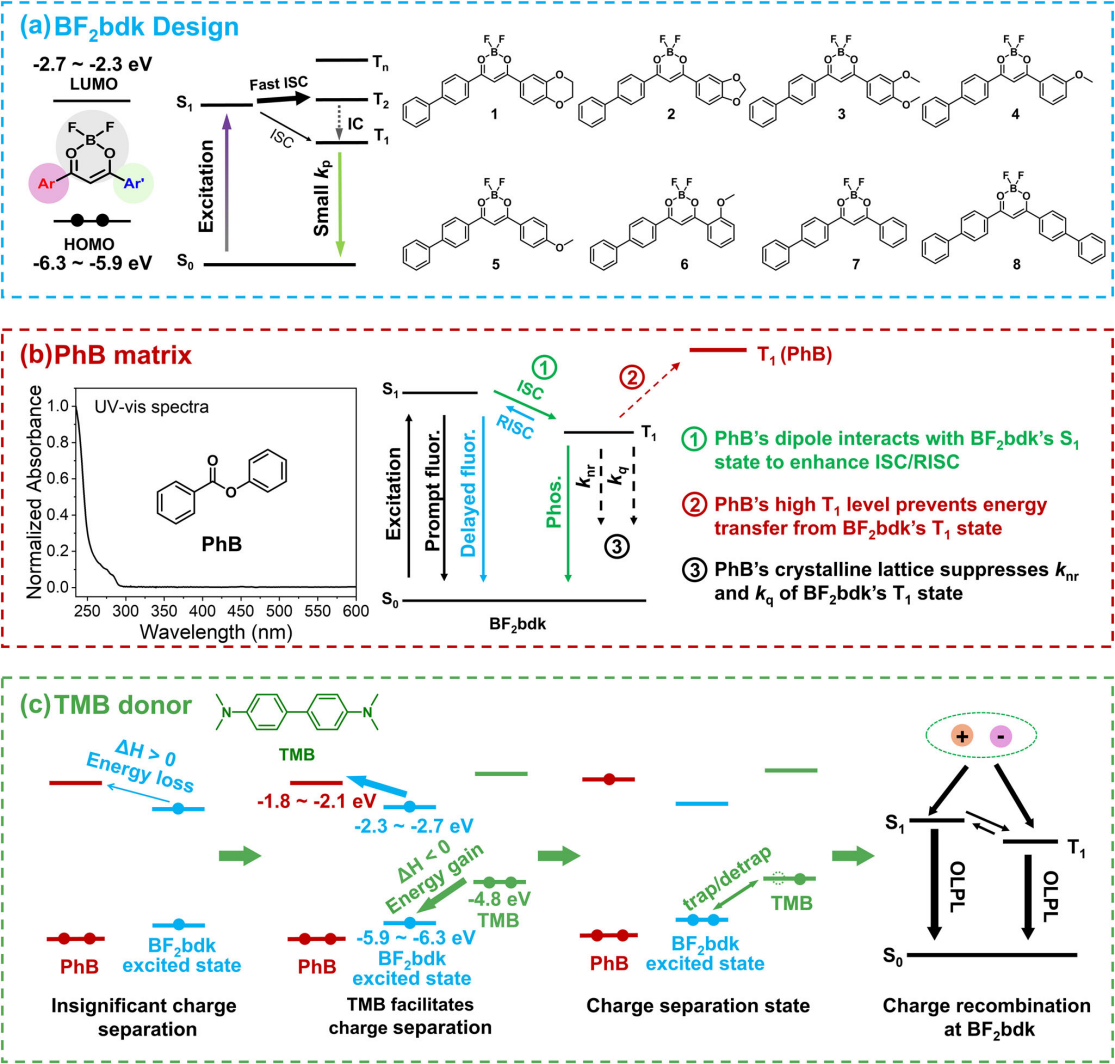
a) BF_2_bdk molecular design and the chemical structures of **BF_2_bdk** compounds in the present study. b) UV–vis spectra of PhB solution in dichloromethane and PhB's role in ISC/RISC enhancement, preventing energy transfer from BF_2_bdk's excited states, and suppressing nonradiative decay and oxygen quenching of BF_2_bdk's triplet excited states. c) The selection of TMB donor to facilitate charge separation and the proposed OLPL mechanism in the present **BF_2_bdk**‐TMB‐PhB three‐component system.

Herein, we introduce a third component, that is an electron donor, into the two‐component **BF_2_bdk**‐matrix system, to induce the formation of charge‐separated states in the resultant three‐component system (**Figure** **1**). The significant population of long‐lived **BF_2_bdk** excited states are also critical for enhanced charge separation in the system. Subsequently, retarded charge recombination occurs on the **BF_2_bdk** molecules, leveraging their capability to efficiently harvest singlet/triplet excitons. Consequently, the obtained **BF_2_bdk**‐donor‐matrix three‐component materials exhibit significant OLPL afterglow efficiency of ≈10% in ambient conditions, with afterglow intensities comparable to inorganic Sr_2_Al_14_O_25_/Eu^2+^, Dy^3+^ materials, as well as an OLPL duration exceeding 2 h. Interestingly, the obtained **BF_2_bdk**‐donor‐matrix materials also show excellent visible‐light‐excitable OLPL afterglow characteristics, which can be excited by normal/weak light sources, 405/420 nm violet/blue light, and even white lights from electric torch or indoor lighting to exhibit significant OLPL afterglow. Furthermore, these OLPL afterglow materials can be further fabricated as panel, exhibit promising applications in afterglow displays and information storage. This work not only proposes a new material design strategy for the enrichment of OLPL material types and the construction of high‐performance OLPL systems, but also explores their potential application scenarios, paving the way for their practical application.

## 2 Results and Discussion

### 2.1 **BF_2_bdk** Synthesis and **BF_2_bdk**‐PhB Two‐Component Afterglow Materials

**Figure 2:**
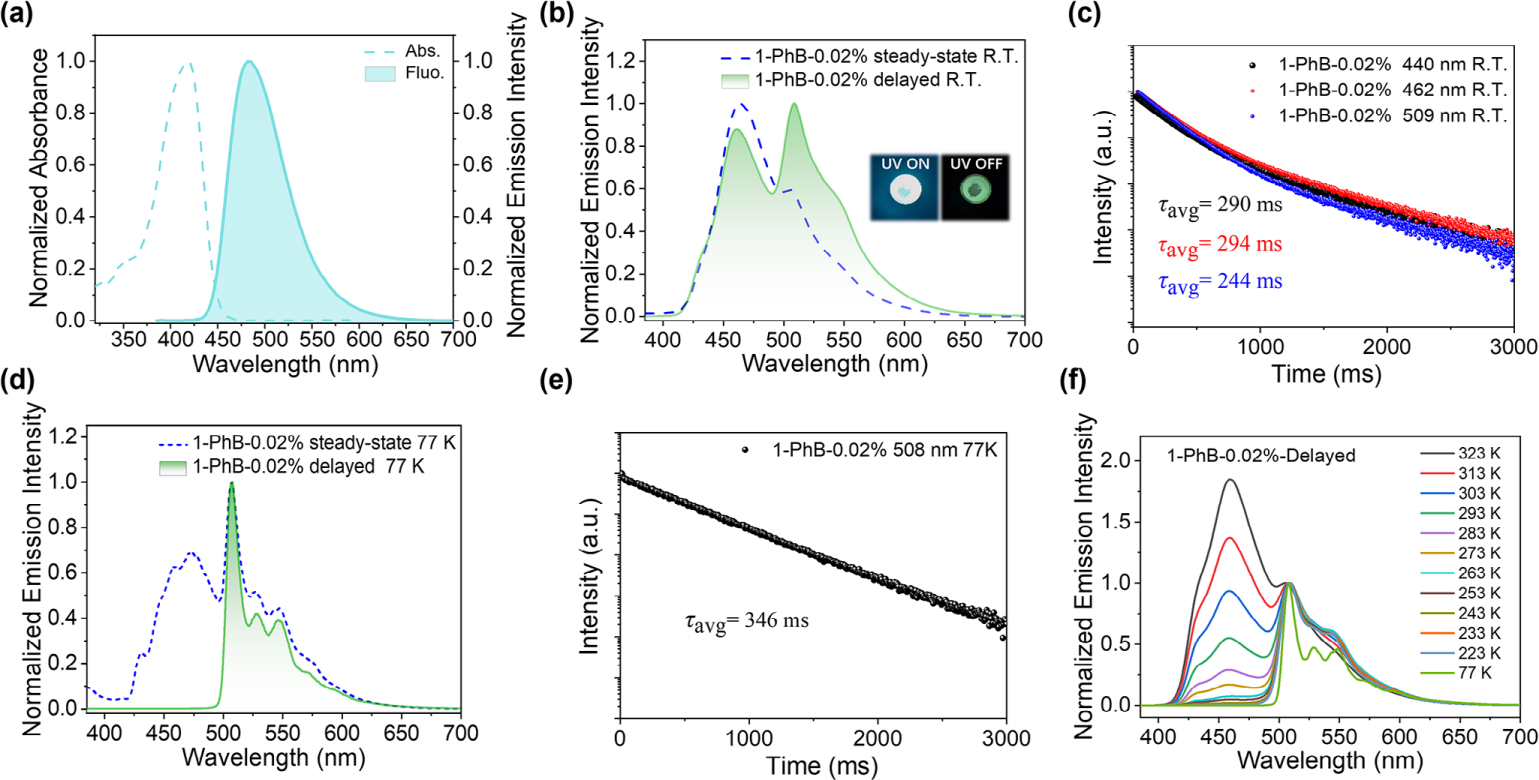
a) UV–vis absorption and steady‐state emission spectra of **1** in dichloromethane solution at room temperature. b) Steady‐state and delayed emission spectra and c) emission decay profiles (monitored at 462 and 509 nm) of **1**‐PhB‐0.02% materials excited at 365 nm at room temperature. d) Steady‐state and delayed spectra of **1**‐PhB‐0.02% materials excited at 365 nm at 77 K. e) Emission decay profiles (monitored at 508 nm) of **1**‐PhB‐0.02% materials excited at 365 nm at 77 K. f) Temperature‐dependent delayed emission spectra (normalized at phosphorescence maxima) of **1‐**PhB‐0.02% samples excited at 365 nm.

All **BF_2_bdk** molecules in Figure 1a were synthesized via Claisen condensation reaction between aromatic ketones and aromatic esters, followed by coordination with boron trifluoride diethyl etherate. After careful column chromatography, the obtained **BF_2_bdk** were further purified via recrystallization in spectroscopic grade dichloromethane/hexane, and their structures were validated through comprehensive characterization methods, while their high purity were confirmed by HPLC measurements (Supporting Information). All these **BF_2_bdk** molecules exhibit excellent photophysical properties in dichloromethane solution, demonstrating large *ε* and high PLQY

(Figure S2a,b and Table S1, Supporting Information). For instance, compound **1** in dichloromethane solution displays a UV‐vis absorption maximum at 419 nm (ε = 5.6 × 10^4^ M^−1^ cm^−1^), this belongs to the intramolecular charge transfer absorption band, and a fluorescence emission band at 472 nm (**Figure** **2**a) with PLQY of 47.0%, and fluorescence lifetime of 2.5 ns (Figure S3, Supporting Information). Compound **3** shows similar luminescent properties to compound **1**, with UV–vis absorption peak at 423 nm (ε = 5.0 × 10^4^ M^−1^ cm^−1^), fluorescence band at 496 nm and PLQY of 44.0% in dichloromethane. In various organic solvents, these **BF_2_bdk** molecules exhibit a pronounced solvatochromic effect, as evidenced by a significant red shift in their steady‐state emission spectra when the solvent is transitioned from *n*‐hexane to ethyl acetate, which correlates with their inherent intramolecular charge transfer characteristics (Figure S2c,d, Supporting Information). This can be further substantiated by time‐dependent density functional theory (TD‐DFT) calculation results, indicating that their excited states indeed exhibit notable intramolecular charge transfer characteristics (Figures S4–S11 and Table S2, Supporting Information).

None of these **BF_2_bdk** molecules show observable room‐temperature afterglow either in solution or in the solid state (Figure S12, Supporting Information). When doped into PhB matrix at a mass fraction concentration of 0.02% by melt casting method (Supporting Information), the obtained **BF_2_bdk**‐PhB melt‐cast samples exhibit bright room‐temperature afterglow in a dark room (Figure S13, Supporting Information). Interestingly, nearly all the obtained **BF_2_bdk**‐PhB melt‐cast samples demonstrate RTP/TADF dual emission characteristics under ambient condition (Figure 2b; Figure S14, Supporting Information), with their photophysical properties comprehensively characterized. For example, the room‐temperature steady‐state and delayed emission spectra of **1**‐PhB‐0.02% melt‐cast sample exhibit identical dual emission peaks at 462 and 509 nm, with PLQY of 67.1% (Figure 2b). The emission peak at 509 nm closely resembles its phosphorescence emission at 77 K (Figure 2d), indicative of RTP‐type afterglow, with phosphorescence lifetime of 244 ms at room temperature (Figure 2c). This phosphorescence emission peak is also remarkably pronounced in the steady‐state spectra both at room temperature and 77 K, suggesting the strong ISC capability of compound **1** (Figure 2d). Notably, the afterglow emission at 462 nm in the delayed spectrum of **1**‐PhB melt‐cast sample closely aligns with the fluorescent emission in the steady‐state spectrum, suggesting that this afterglow emission can be attributed to the TADF originating from **BF_2_bdk**’s S_1_ states, as previously demonstrated in other **BF_2_bdk**‐PhB system reported by us.^[^ ^38^, ^42^ ^]^

In order to further elucidate the mechanism underlying the afterglow emission of **1**‐PhB melt‐cast sample, as well as other PhB melt‐cast samples, a thorough investigation and discussion of of alternative afterglow mechanisms have been conducted in Text S1 (Supporting Information) and subsequently ruled out. Temperature‐dependent photoluminescence experiments have been further performed to explore the excited‐state properties of **BF_2_bdk**‐PhB melt‐cast samples (Figure 2f; Figure S15, Supporting Information). For example, at 77 K, the delayed emission spectrum of the **1**‐PhB‐0.02% sample displays a distinct phosphorescence emission ranging from 490 to 700 nm, with a maximum emission peak at 508 nm, thus allowing for an estimation of compound **1′**s T_1_ level at 2.44 eV. As the temperature increases, the afterglow emission at 458 nm in the delayed emission spectrum became more prominent, and ultimately dominating at 313 K. This temperature‐enhanced afterglow emission is characteristic of TADF or triplet‐triplet annihilation (TTA) emission, the latter being negligible at such low doping concentration (0.02 wt%). Based on the aforementioned experiments, it can be confirmed that the afterglow emission at 458 nm of **1**‐PhB‐0.02% melt sample is attributable to long‐lifetime TADF originating from compound **1′**s S_1_ emission, allowing for an estimation of compound **1′**s S_1_ level at 2.71 eV. The *k*_RISC_ of BF_2_bdk is in the range of 10^0^–10^1^ s^−1^, which is substantially smaller than those observed in conventional thermally activated delayed fluorescence (TADF) materials. In solution phase, the enhanced molecular motion of BF_2_bdk significantly increases non‐radiative decay rates. This *k*_RISC_ value of 10^0^–10^1^ s^−1^ becomes negligible compared to both the non‐radiative decay rate (*k*_nr_) and oxygen quenching (*k*_q_) in solution phase, resulting in complete suppression of TADF characteristics. In contrast, in solid‐state sample, the crystalline PhB matrix effectively restricts intramolecular motion of BF_2_bdk excited states and inhibits oxygen diffusion into its lattice structure to quench triplet excited states, thereby providing protection for the excited states of BF_2_bdk. Under this condition, *k*_nr_ and *k*_q_ become much smaller than *k*_RISC_, allowing the material to exhibit TADF characteristics. Thus, **1**‐PhB‐0.02% melt‐cast sample, as well as other **BF_2_bdk**‐PhB melt‐cast samples in this work, exhibits the distinctive RTP/TADF dual emission characteristics, which can also be corroborated by theoretical calculations (Figures S4–S11 and Table S3, Supporting Information). Regarding the S_1_‐T_1_ gap and RISC process, we understand that TADF emitters for OLED have *k*_RISC_ in the range of 10^3^–10^6^ s^−1^, so usually a small S_1_‐T_1_ gap of 0.2 eV or ≈0.1 eV is required to facilitate RISC process.^[^ ^50^ ^]^ It has been reported that *k*_RISC_ is sensitive to S_1_‐T_1_ gap; an increase of S_1_‐T_1_ gap by 0.1 eV would lead to a decrease of *k*_RISC_ by roughly 100 fold.^[^ ^51^ ^]^ In the present study, a S_1_‐T_1_ gap of 0.27 eV corresponding to a moderate *k*_RISC_ of 10^0−^10^1^ s^−1^ is reasonable. The TD‐DFT calculation results indicate that BF_2_bdk possesses rich excited‐state characteristics, with multiple T_n_ states exhibiting symmetries distinct from that of the S_1_ state; such differences in symmetry are favorable for ISC and RISC process, as per the EI‐Sayed rules. For example, compound **1** possesses substantial SOCME (T_1_‐S_1_) of 0.23 cm^−1^, SOCME (T_2_‐S_1_) of 0.35 cm^−1^, and SOCME (T_3_‐S_1_) of 0.62 cm^−1^. The SOCME between these T_n_ states and S_1_ are notably large for pure organic compound, facilitating the formation of various ISC and RISC pathways, particularly involving T_1_‐to‐T_2_‐to‐S_1_ and T_1_‐to‐T_3_‐to‐S_1_ RISC channels. Some reported studies also support the presence of these RISC channels bridged by T_2_ and T_3_ states.^[^ ^51^, ^52^, ^53^, ^54^, ^55^ ^]^ Based on prior TD‐DFT calculation experience with BF_2_bdk from our group,^[^ ^38^, ^42^, ^43^, ^44^, ^45^, ^47^ ^]^ the calculated triplet energy levels of **BF_2_bdk** (by TD‐DFT at B3LYP/6‐31g(d,p) level using optimized ground‐state geometry) agrees well with the experimental values; whereas, the singlet energy levels only display correspondence in UV–vis absorption, and discrepancies in the fluorescence emission, so we use experimental S_1_ energy level estimated from fluorescence maximum for the discussion of ISC/RISC. Table S3 (Supporting Information) summarizes the excited states properties of **BF_2_bdk**‐PhB‐0.02% melt‐cast samples, including both experimental and calculated values. For instance, the experimental T_1_ level at 2.44 eV of compound **1**, estimated from the phosphorescence maximum of **1**‐PhB‐0.02% melt‐cast sample, closely corresponds with the calculated value (2.368 eV), while its experimental S_1_ level (2.71 eV) and calculated T_2_ level (2.817 eV) also exhibit strong proximity. Such close alignment between the S_1_ and T_2_ levels adeptly explains the pronounced ISC characteristics of compound **1**, as well as the RTP/TADF dual emission characteristic of **1**‐PhB‐0.02% sample; as T_2_ can serve as a bridge to mediate S_1_‐T_n_ ISC and RISC processes. Compounds **2** and **4** have also been found to exhibit strong ISC efficiency as revealed by steady‐state emission spectra of BF_2_bdk‐PhB‐0.02% samples at 77 K (Figure S16b,d, Supporting Information) and corroborated by theoretical analysis (Table S3, Supporting Information). For compounds **3**, **5** to **8**, experimental and theoretical results show that their ISC tendency are still significant (Figure S16c,e–h and Table S3, Supporting Information) but less than compounds **1**, **2**, and **4**.

### 2.2 Adding the Third Component for Fabricating OLPL Materials

To further extend the afterglow durations, the electron donor TMB was introduced as the third component into the **BF_2_bdk**‐PhB two‐component system. After melting **BF_2_bdk**, TMB and PhB at a mass ratio of 2: 2: 1000 followed by room‐temperature cooling, **BF_2_bdk**‐TMB‐PhB‐0.2% melt‐cast samples are capable of being fabricated as bulk solid or film of different thickness, and bulk solid‐shaped samples exhibit significant afterglow persisting for over 30 min under ambient conditions (Figures S17 and S18, Supporting Information), especially for compounds **1**, **2**, and **3**. Regarding thermal stability of the components, we performed differential scanning calorimetry (DSC) and thermogravimetric analysis (TGA) for BF_2_bdk. For example, compound **1** has been found to show a melting point of 242 °C in its DSC profile (Figure S19a, Supporting Information). In its TGA curve, the very first weight loss ≈100 °C is from the evaporation of residual solvents in the lattice of compound **1**, and the weight% remains constant from 100 to 260 °C (Figure S19b, Supporting Information). TMB has a melting point of 193–195 °C and its TGA curve shows negligible weight loss from room temperature to 150 °C (Figure S19c, Supporting Information). PhB shows a melting point of ≈70 °C and a boiling point of ≈300 °C at normal pressure. Insignificant weight loss has been observed in the range of 30–70 °C. The weight loss above 80 °C can be ascribed to vaporization of PhB after melting, rather than thermal decomposition (Figure S19d, Supporting Information). Based on these results, all the three components are thermally stable at 80 °C during afterglow material fabrication.

**Figure 3:**
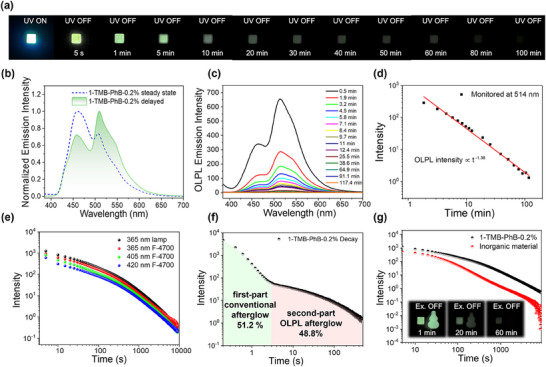
a) Photographs of **1**‐TMB‐PhB‐0.2% materials under a 365 nm UV lamp and after removal of the UV lamp. b) Room‐temperature steady‐state and delayed emission spectra of **1**‐TMB‐PhB‐0.2% materials excited at 365 nm. c) Delayed emission spectra of the **1**‐TMB‐PhB‐0.2% materials excited at 365 nm as a function of delay time. d) Logarithmic plot of the OLPL emission intensity (monitored at 514 nm) of **1**‐TMB‐PhB‐0.2% materials at different delayed times. e) Room‐temperature emission decay profiles (monitored ≈514 nm) of **1**‐TMB‐PhB‐0.2% materials excited at 365 nm lamp (a handheld UV lamp, 5 W), 365 nm, 405 nm and 420 nm (excitation wavelengths of F‐4700, EX/EM slits at 20/20 nm). f) Room‐temperature afterglow decay profiles (1 ms delay, monitored at 514 nm, excited at 365 nm) of **1**‐TMB‐PhB‐0.2% materials. g) Room‐temperature emission decay profiles of **1**‐TMB‐PhB‐0.2% materials mointered at 514 nm excited at 365 nm and inorgnaic Sr_2_Al_14_O_25_/Eu^2+^, Dy^3+^ materials mointered at 518 nm excited by room light. The inset shows photographs of **1**‐TMB‐PhB materials after removal of 365 nm UV lamp (left) and Sr_2_Al_14_O_25_/Eu^2+^, Dy^3+^ materials (right) after ceasing room light.

Significantly, the obtained **1**‐PhB‐TMB‐0.2% melt‐cast sample displays a green afterglow lasting for over 120 min in a dark room after the removal of a 5 W 365 nm light source (**Figure** **3**a,b), which can still be detected in its delayed emission spectra even 2 h post‐excitation (Figure 3c); such a long afterglow duration aligns with the characteristics of OLPL afterglow.^[^ ^17^, ^18^, ^19^, ^20^, ^21^, ^22^, ^23^, ^24^, ^25^, ^26^, ^27^, ^28^ ^]^ We repeat the photophysical measurements on **1**‐TMB‐PhB‐0.2% materials to study the reproducibility (Figure S20, Supporting Information). Three independent **1**‐TMB‐PhB‐0.2% samples were prepared parallelly. The OLPL spectra exhibited nearly identical afterglow intensities and decay kinetics across all samples, with error bars further confirming excellent data reproducibility (Figure S20d, Supporting Information). Moreover, the afterglow intensity of **1**‐PhB‐TMB‐0.2% melt‐cast sample, which is obtained from the delayed emission spectra collected at different delay time, exhibit a power‐law decay relationship with respect to the delay time, specifically afterglow intensity ≈t^−1.36^ (Figure 3d). This power‐law decay conforms to Debye‐Edwards law, where intensity ≈t^−m^, and can be used to elucidate the retarded charge recombination processes in OLPL materials.^[^ ^56^ ^]^ Here, m represents the rate of charge recombination within the range of 0–2, the larger the value of m, the faster the charge recombination process.^[^ ^18^ ^]^ The above results robustly support that the prolonged afterglow of the **1**‐TMB‐PhB‐0.2% melt‐cast sample belongs to the OLPL afterglow. It should be noted that, similar to the steady‐state and delayed emission spectra of the **1**‐PhB melt‐cast sample, the delayed emission spectra (delay time ≥0.3 min, also referred to as OLPL spectra) of the **1**‐TMB‐PhB melt‐cast sample also exhibit dual‐emission characteristics, with emission peaks at 460 and 514 nm (Figure 3c). The OLPL afterglow emission peak at 460 nm closely resembles the TADF emission of **1**‐PhB melt‐cast sample (originating from compound **1′**s S_1_ emission), while the OLPL afterglow emission peak at 514 nm is comparable to the phosphorescence emission of **1**‐PhB melt‐cast sample (originating from compound **1′**s T_1_ emission) (Figures 2b and 3c); this indicates that the OLPL afterglow of **1**‐TMB‐PhB sample ultimately manifests as dual emission from compound **1′**s S_1_ and T_1_ states. It is noteworthy that the afterglow decay profile of **1**‐TMB‐PhB‐0.2% melt‐cast sample indicate that its OLPL afterglow can last for over 9000 s (Figure 3e). The PLQYs of **1**‐PhB‐0.2% and **1**‐PhB‐TMB‐0.2% samples are determined to be 57.3% and 20.6%, respectively. It is evident that with the addition of TMB, a substantial reduction of 36.7% (derived from 57.3% – 20.6%) in PLQY occurs. This can be ascribed to a greater number of excitons being conserved for the retarded OLPL afterglow emission in **1**‐TMB‐PhB‐0.2% sample, which shows a significant charge separation process compared to **1**‐PhB‐0.2% sample, the difference of 36.7% can be approximately correlated to the percentage of excited states of **1** that partake in the charge separation process. A significant decrease in PLQY means a large extent of charge separation, which is a prerequisite for high‐performance OLPL. Figure 3f shows that the afterglow decay profile exhibits two distinct decay modes, in which the second part of the decay profile (≈3 s post‐excitation) follows a power‐law decay relationship and can be attributed to OLPL afterglow. Conversely, the first part of the decay profile (upon ceasing excitation to delay time of 3 s) follows an exponential decay pattern similar to the afterglow decay profile of the **1**‐PhB melt‐cast sample (Figure S21a, Supporting Information); this implies that the first decay part can be attributed to conventional RTP/TADF afterglow. Notably, the **1**‐TMB‐PhB materials can be readily excited by the excitation source of Hitachi F‐4700 fluorescence spectrometer to display emission decay profile with two distinct decay modes (Figure 3f). The proportions of the first‐part conventional afterglow and the second‐part OLPL afterglow calculated to be 51.2% and 48.8%, respectively. If one can obtain the efficiency of the first‐part conventional afterglow, it is possible to estimate the OLPL efficiency by the emission decay profile and the afterglow proportions (Figure 3f). The PLQY of **1**‐TMB‐PhB‐0.2% materials at room temperature has been measured to be 20.6% by integrating sphere measurement system. **1**‐TMB‐PhB materials show fluorescence and phosphorescence bands in the steady‐state emission spectra. To calculate their conventional afterglow efficiency, one can first obtain the percentage of phosphorescence component from the steady‐state emission spectra and then the afterglow efficiency is the product of PLQY and phosphorescence percentage. However, the percentage of phosphorescence component is interfered by RISC processes for the **1**‐TMB‐PhB materials at room temperature, so we use the percentage of phosphorescence component of 44.39% in 77 K steady‐state emission spectra (Figure S36a, Supporting Information; **Table** **1**) for the calculation. The resultant value of conventional afterglow efficiency is estimated to be 9.1% (20.6% × 44.39%). While the second part of the afterglow decay profile, that is OLPL afterglow, remains undetectable by current PLQY measurement instruments due to its extended afterglow duration lasting minutes and even hours. Based on the proportion of the first‐part conventional afterglow (51.2%) and the second‐part OLPL afterglow (48.8%) from Figure 3f, the OLPL efficiency of **1**‐TMB‐PhB‐0.2% sample can be estimated as 8.7% (9.1% × 48.8%/51.2%) using the value of first part conventional afterglow efficiency of 9.1%. **2**‐TMB‐PhB‐0.2% melt‐cast sample also display significant OLPL behavior under ambient condition (Figure S21b, Supporting Information) with OLPL based on efficiency estimated to be 11.6% (Figure S21d, Supporting Information) based on the above method (Text S2, Supporting Information). It is known that OLPL efficiency is dependent on concentration of charge‐separated states, here we only give a rough estimate of OLPL efficiency.

**Table 1:** Photophysical data of BF_2_bdk‐TMB‐PhB materials.

Entry	Steady‐state emission (77 K)		Delayed emission (R.T.)		Intensity^a)^	Duration time^b)^/s	m value^c)^	PLQY^d)^
	S_1_ /%	T_1_ /%	S_1_ /%	T_1_ /%				
1	55.61	44.39	36.68	63.32	655	>9000	1.35	20.6%
2	47.46	52.54	20.99	79.01	555	>9000	1.31	25.0%
3	60.68	39.32	49.25	50.75	359	>9000	1.20	15.8%
4	74.46	25.54	14.01	85.99	129	>9000	1.17	11.8%
5	71.00	29.00	14.23	85.77	132	>9000	1.23	17.6%
6	37.00	63.00	12.33	87.67	31	2000	1.14	9.0%
7	57.05	42.95	4.69	95.31	36	3000	1.18	11.7%
8	76.70	23.30	13.11	86.89	120	7000	1.14	12.0%

^a)^ Intensity of emission maxima in the OLPL emission spectra at delay time of ≈0.5 min;

^b)^ Estimated from OLPL emission decay profiles;

^c)^ Fitted from the logarithmic plot of the OLPL emission intensity versus delayed times;

^d)^ Photoluminescence quantum yields measured by instrument equipped with integrating sphere system.

Notably, for afterglow material fabrication, the **BF_2_bdk** and TMB compounds have been purified via recrystallization before use, with their high purity verified by HPLC measurements (please find figures in the structural and purity characterization part and Figure S22a, Supporting Information). PhB matrix was used as received. To rule out the possible effect caused by impurity, we also performed recrystallization to obtain PhB’ with high purity (Figure S22b, Supporting Information). **1**‐TMB‐PhB and **1**‐TMB‐PhB’ melt‐cast samples show similar steady‐state and delayed emission spectra as well as afterglow decay profiles (Figure S23, Supporting Information), confirming that the OLPL afterglow emission of **1**‐TMB‐PhB melt‐cast sample are not caused by impurities. Also, three‐component samples were prepared using commercially available and distilled DCM solvents, respectively. Both samples exhibited similar steady‐state and delayed spectra and decay profiles, indicating that trace amount of impurities in the solvents had no impact on the properties of the three‐component materials (Figure S24, Supporting Information). Furthermore, the excitation spectra of BF_2_bdk‐TMB‐0.2% samples exhibit analogous peak maxima to those of the UV–vis absorption spectra of BF_2_bdk in DCM solution (Figures S2a and S25, Supporting Information). The congruence indicates that the luminescent properties of BF_2_bdk‐TMB‐0.2% samples are ascribed to BF_2_bdk dopants rather than from impurities. The duration of **1**‐TMB‐PhB‐0.2% sample is related to the excitation power and time (Figure S26, Supporting Information). It was found that the intensity and duration of OLPL increased with the enlargement of excitation power (Figure S26a, Supporting Information). Interestingly, an obvious OLPL signal could still be observed with excitation power of 2 µM/cm^2^, which indicates that OLPL phenomenon of this three‐component system is unlikely to be caused by two‐photon ionization mechanism. Meanwhile, when fixing excitation power at 2.1 mM/cm^2^, we changed the excitation time from 5 s to 1 min and found that the OLPL intensity and duration gradually increased. This is because the charge‐separated state formed in the material gradually increases with the increase of excitation time. After the excitation time exceeds 1 min, the increase in OLPL intensity and duration of the material is very small (Figure S26b, Supporting Information), as the charge‐separated state that the material can form reaches a threshold under a long excitation time. Additionally, our material exhibits similar decay curves in both atmospheric and vacuum environments (Figure S26c, Supporting Information), suggesting that the material is well protected by the PhB matrix and the formed radical ions will not be quenched by oxygen. Besides, **1**‐TMB‐PhB melt‐cast sample can be excited by both 405 and 420 nm light (Figure S27, Supporting Information) resulting in OLPL afterglow properties (including OLPL emission spectra and afterglow decay curves) similar to those obtained by 365 nm excitation (Figure S27, Supporting Information). Impressively, **1**‐TMB‐PhB melt‐cast sample can also be excited by the electric torch of a common mobile phone and exhibit a decent OLPL afterglow (Figure S27, Supporting Information), demonstrating its excellent visible‐light‐excitable OLPL afterglow characteristic. Additionally, a comparison with the afterglow emission spectra and afterglow decay curves of inorganic Sr_2_Al_14_O_25_/Eu^2+^, Dy^3+^ materials reveals that the OLPL performance of **1**‐TMB‐PhB melt‐cast sample is comparable to that of inorganic Sr_2_Al_14_O_25_/Eu^2+^, Dy^3+^ materials (Figure 3g; Figure S28, Supporting Information), thereby validating the feasibility of the proposed three‐component design strategy for achieving high‐performance OLPL materials in this work.

Previous literature has utilized TMB as an electron donor to form exciplex with the electron acceptor PPT to generate hours‐long OLPL afterglow emission.^[^ ^17^, ^34^ ^]^ However, in this work, the obtained TMB‐PhB‐0.2% melt‐cast sample can only exhibit a very weak green afterglow, with insignificant OLPL emission detected (Figure S29, Supporting Information). Besides, TMB‐PhB melt‐cast sample exhibits a similar UV–vis absorption to TMB‐COP sample (Figure S29a, Supporting Information), where COP refers to cyclo olefin polymer. The steady‐state emission spectrum of TMB‐PhB melt‐cast sample closely resemble that of the TMB‐COP sample, except that the fluorescence maxima is red‐shifted from 389 nm (for TMB‐COP sample) to 403 nm (for TMB‐PhB sample) due to the increase of matrix's polarity (Figure S29b, Supporting Information). Notably, the steady‐state emission spectra of TMB‐PhB sample also exhibit a small emission peak at 500 nm (Figure S29b, Supporting Information), which corresponds to the phosphorescence emission observed in the steady‐state and delayed emission spectra of both TMB‐COP and TMB‐PhB samples at 77 K (Figure S30, Supporting Information); this phosphorescence emission can be attributed to TMB's phosphorescence emission as reported.^[^ ^17^ ^]^ The phosphorescence emission in the steady‐state emission spectrum of TMB‐PhB melt‐cast sample is due to the protective environment provided by crystalline PhB matrix that prevents TMB's T_1_ states from the oxygen quenching, whereas COP allows oxygen permeation under ambient conditions, thus diminishing the phosphorescence emission. The above experimental results and discussions can rule out the possibility of TMB‐PhB forming an exciplex. Moreover, it has been reported that TMB‐PPT exciplex can also serve as the long‐lived energy source to deliver its persistent luminescence to the doped emitters via energy transfer processes, resulting in multi‐color long‐lived OLPL afterglow.^[^ ^29^, ^32^ ^]^ Although TMB is also employed as an electron donor in this work, the OLPL afterglow mechanism of the **1**‐TMB‐PhB system diverges from those reported OLPL afterglow system based on donor‐acceptor mechanisms, including exciplex as well as the exciplex OLPL energy transfer systems, primarily due to the following reasons: 1) No significant OLPL afterglow is observed and detected for TMB‐PhB‐0.2% melt‐cast sample under 365 nm excitation; 2) compared to **1**‐TMB‐PhB‐0.2% melt‐cast sample, 4CzPN‐TMB‐PhB‐0.2% and NBN‐TMB‐PhB‐0.2% melt‐cast samples (the chemical structures of NBN and 4CzPN dopants can be found in (Figure S31a,d, Supporting Information) show insignificant OLPL afterglow at room temperature (Figure S31c,f, Supporting Information), which suggest that the exciplex OLPL energy transfer is unlikely to occur in the present system; 3) **1**‐TMB‐PhB‐0.2% melt‐cast sample can be excited by visible light (such as 405 or 420 nm) and exhibit a remarkable OLPL afterglow under ambient conditions, while the UV‐vis absorption of TMB‐PhB melt‐cast sample at 365 nm or even 405 nm is very weak or almost negligible (Figure S29a, Supporting Information), with only compound **1** showing visible absorption properties (Figure S2, Supporting Information). Hence, the photophysical processes of OLPL afterglow are most likely to begin in the photo‐excitation of **BF_2_bdk** molecules. On the other hand, the absence of OLPL afterglow in the 4CzPN‐TMB‐PhB‐0.2% and NBN‐TMB‐PhB‐0.2% samples (4CzPN and NBN have TADF lifetimes of ≈1 ms or shorter) suggest that a long‐lived T_1_ or S_1_ states (emission lifetime, ≈100 ms or longer) should be also necessary for the enhanced charge separation process in the present **BF_2_bdk**‐TMB‐PhB system.

**Figure 4:**
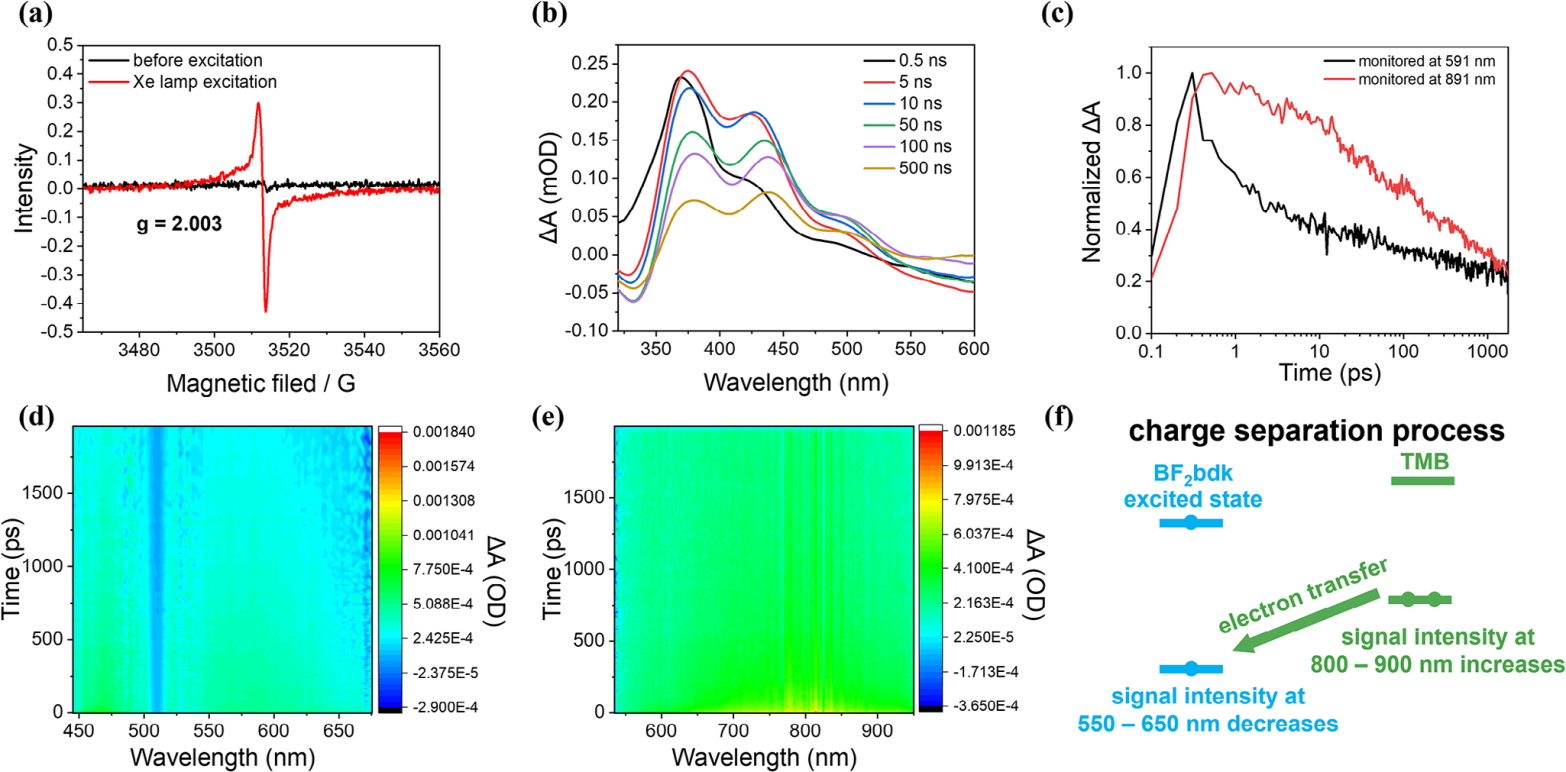
a) Electron spin resonance spectra of **1**‐TMB‐PhB‐0.2% materials before excitation and after excitation with a Xenon lamp. b) The transient absorption spectra of 1‐TMB‐PhB‐0.2% materials (excited by a 290 nm pump laser) collected at delay times from 0.5 to 500 ns. c) The transient absorption decay kinetics (excited by a 400 nm pulse laser) monitored at 591 and 891 nm of **1**‐TMB‐PhB‐0.2% materials. d) Transient absorption spectra of **1**‐TMB‐PhB‐0.2% materials (excited by a 400 nm pulse laser) collected at 430–670 nm. e) Transient absorption spectra of **1**‐TMB‐PhB‐0.2% materials (excited by a 400 nm pulse laser) collected at 540–950 nm. f) A scheme illustrates for electron transfer process from ground‐state TMB to excited‐state BF_2_bdk.

To further elucidate the mechanism underlying the long‐persistent afterglow of the **1**‐TMB‐PhB‐0.2% sample, we performe electron spin resonance (ESR) of **1**‐TMB‐PhB‐0.2% sample, the presence of radical species can be confirmed by ESR signal (**Figure** **4**a), which is similar to that of the TMB radical cation in the reported studies.^[^ ^57^, ^58^ ^]^ However, the ESR signal may still include contributions from BF_2_bdk radical cation and PhB radical anion. Transient absorption spectra (TAS) has been employed to investigate the radical intermediates potentially involved in the charge separated and charge recombination processes. The 350–410 nm, 410–480 nm, and 480–540 nm TAS bands can be assigned to PhB radical anion, TMB radical cation, and **1′**s exciton or radical cation, respectively (Figure 4b).^[^ ^59^, ^60^ ^]^ We also perform fs‐TAS experiment to acquire more evidence for the proposed electron transfer process (Figure 1c). Upon excited by a 400 nm pulse laser where TMB has orders of magnitude smaller ground‐state absorption than compound **1**, the transient absorption spectrum of **1**‐TMB‐PhB‐0.2% shows **1′**s excited state absorption signal from 540 to 660 nm, as well as two sets of signals of TMB radical cation in 440–490 nm and 700–900 nm regions (Figure 4d,e). Since the 400 nm pulse laser selectively excite compound **1**, the emergence of TMB radical cation signals should be the consequence of electron transfer from ground‐state TMB to excited‐state BF_2_bdk. Importantly, from the normalized decay profile of the transient absorption signals, at 0.33 ps, a reduction of **1′**s excited state absorption signal at 591 nm correlates with an augmentation of TMB radical cation signal at 891 nm (Figure 4c). This supports our proposed process of electron transfer from TMB to BF_2_bdk in the charge separation process (Figures 1c and 4f). We attempted to verify the electron transfer process from **BF_2_bdk** to PhB. However, since the absorption signal of the PhB radical anion lies in the 350–400 nm range, we conducted additional fs‐TAS experiments with shorter‐wavelength pulse lasers. Due to strong interference from the luminescence (negative signal) of TMB overlapping this spectral region, we have not yet obtained direct evidence for electron transfer from **BF_2_bdk** to PhB. We will strive to address this issue in future work. Based on these experimental results and analyses, we propose that the OLPL afterglow emission of the **1**‐TMB‐PhB melt‐cast sample involves two electron transfer processes leading to the formation of charge‐separated states, and the specific OLPL mechanism is as follows (Figure 1c): 1) upon irradiation, **BF_2_bdk** molecules are excited, accompanied by the transition of an electron from the HOMO to the LUMO; 2) an electron in the excited **BF_2_bdk**’s LUMO (−2.7–−2.3 eV) is transferred to PhB's LUMO (−1.8–−2.1 eV), while an electron in TMB's HOMO (−4.8 eV) donates to **BF_2_bdk**’s HOMO (−6.3–−5.9 eV), resulting in charge separation and the formation of PhB radical anions and TMB radical cations; 3) the retarded charge recombination occurs, which regenerates the excited **BF_2_bdk** and releases the OLPL afterglow lasting several hours. However, as for **1**‐TMB‐PhB‐0.2% melt‐cast sample, the low doping concentration of compound **1** and TMB (only 0.2%) results in considerable spatial separation around the PhB matrix, leading to the doubts about the feasibility of such an electron transfer process. Consequently, we conducted a model experiment by doping traditional donor‐acceptor pairs: tetrathiafulvalene (TTF) and 2,3,5,6‐tetrafluoro‐1,4‐benzoquinone (TFBQ) into the PhB matrix at the same low concentration (0.2 wt%) to investigate the electron transfer process. Notably, the obtained **TTF‐TFBQ**‐PhB‐0.2% sample exhibits a purple or brown color in daylight as compared to **TTF**‐PhB‐0.2% and **TFBQ**‐PhB‐0.2% (Figure S32, Supporting Information), which confirms that a doping concentration of 0.2 wt% is already sufficient to facilitate electron transfer between dopants. This model experiment supports the proposed electron transfer from TMB to **BF_2_bdk** at 0.2% doping concentration in the present system. On the other hand, the long‐lived excited state characteristics of **BF_2_bdk** (emission lifetime, ≈100 ms or longer) would increase the likelihood of this electron transfer process occurring. Regarding the dispersion states of the dopants, we first perform concentration‐dependent photophysical studies on **1**‐PhB two‐component samples. At 0.02% doping concentration, the room‐temperature steady‐state emission spectra of **1**‐PhB‐0.02% sample show a fluorescence band at 462 nm, as well as phosphorescence signal at 509 nm (Figure S33b, Supporting Information). Upon increasing the doping concentration to 0.2%, the steady‐state emission spectra exhibit significant change in spectral shape, and the fluorescence band shows a red shift (Figure S33a, Supporting Information), which suggests the presence of **BF_2_bdk** molecular aggregates in **1**‐PhB‐0.2% sample. Interestingly, **1**‐TMB‐PhB‐0.2% three‐component sample has been found to show similar steady‐state emission spectra to **1**‐PhB‐0.02% sample (Figure S33b,c, Supporting Information). These suggest the de‐aggregation of BF_2_bdk in **1**‐TMB‐PhB‐0.2% sample, which should be caused by the association of TMB and **BF_2_bdk**. The association of TMB and **BF_2_bdk** in PhB matrix can better explain the electron transfer from TMB to **BF_2_bdk** in the charge separation process. Our system demonstrates a unique charge separation mechanism involving two electron transfer processes: 1) electron transfer from the LUMO of **BF_2_bdk** to the LUMO of the PhB matrix; 2) electron transfer from the HOMO of TMB to the HOMO of **BF_2_bdk**. This is distinct from the reported donor‐acceptor and TPI OLPL systems. Significant OLPL afterglow can be achieved by normal or weak photo‐excitation in donor‐acceptor systems (which is an advantage over TPI OLPL systems that require high‐power excitation), whereas most donor‐acceptor exciplex systems show moderate or modest PLQY due to the inherently small oscillator strength of intermolecular charge‐transfer states. The present three‐component system allows normal/weak photo‐excitation and exhibits excellent light absorption and PLQY because of the large oscillator strength of **BF_2_bdk** luminophores. In addition, compared to the expensive electron‐transport materials such as PPT and TPBi, the cost‐effective PhB offers superior practicality for real‐world applications.

Similar to **1**‐TMB‐PhB and **2**‐TMB‐PhB materials, other **BF_2_bdk**‐TMB‐PhB samples also exhibit OLPL afterglow (Figure S34, Supporting Information). The comparison of the OLPL afterglow decay profiles as well as the OLPL emission spectra (0.5 min delay) of **BF_2_bdk**‐TMB‐PhB samples reveals that **1**‐TMB‐PhB and **2**‐TMB‐PhB samples demonstrate particularly remarkable OLPL afterglow performance (Table 1). In contrast, the OLPL afterglow of **3**‐TMB‐PhB, **4**‐TMB‐PhB, and **5**‐TMB‐PhB samples is moderate, while the OLPL of **6**‐TMB‐PhB, **7**‐TMB‐PhB, and **8**‐TMB‐PhB is relatively weak (Figure S35, Supporting Information; Table 1). From their OLPL emission spectra at various delay times, we can see that the OLPL afterglow intensity of all the **BF_2_bdk**‐TMB‐PhB samples follows the power‐law decay with intensity ≈t^−m^ (Figure S34, Supporting Information). **1**‐TMB‐PhB and **2**‐TMB‐PhB samples have larger m values (m = 1.35, 1.31), indicating a faster emission decay kinetics than other **BF_2_bdk**‐TMB‐PhB samples (m = 1.14–1.23, Table 1). Despite of this, **1**‐TMB‐PhB and **2**‐TMB‐PhB samples still show bright and long‐lasting afterglow, which suggest the involvement of high‐concentration radical intermediates in these two systems, that is, the enhanced charge separation processes. To better understand the differences in the OLPL afterglow performance among various **BF_2_bdk**‐TMB‐PhB samples, further in‐depth analyses of their photophysical data have been performed. First, after peaks separation, the contributions of S_1_ and T_1_ states emissions in the steady‐state emission spectra of **BF_2_bdk**‐TMB‐PhB samples at 77 K have been calculated (Table 1). The results indicate that all **BF_2_bdk**‐TMB‐PhB samples exhibit a certain proportion of T_1_ states emission in their 77 K steady‐state emission spectra, especially when **BF_2_bdk** is compounds **1** and **2,** where their T_1_ states emission proportion is ≈50% (Figure S36, Supporting Information; Table 1). Such a high proportion of T_1_ emission suggests that compounds **1** and **2** possess strong ISC capabilities, which favor the generation of more long‐lived **BF_2_bdk**’s states, thereby promoting the occurrence of charge separation and enhancing OLPL afterglow performance; the strong ISC capability of **BF_2_bdk** is also well demonstrated in the **BF_2_bdk**‐PhB systems above, as well as the TD‐DFT calculation results (Figures S4 and S5, Supporting Information). Additionally, these **BF_2_bdk**‐TMB‐PhB samples all display S_1_ and T_1_ dual afterglow emission characteristics, with commendable PLQY (Figure S37, Supporting Information; Table 1). The delayed emission spectra of **BF_2_bdk**‐TMB‐PhB samples have also been performed with peak separation, from which the contributions of TADF (S_1_ states emission) and RTP (T_1_ states emission) can be calculated (Table 1). The findings reveal a notable S_1_ contribution in the delayed emission spectra of **BF_2_bdk**‐TMB‐PhB samples, indicating that **BF_2_bdk** also possesses a respectable RISC capability; a strong RISC capability can facilitate the transition of T_1_ states to S_1_ states, enabling the efficient harvesting of singlet/triplet excitons. Besides, the balance between RISC and ISC facilitates the sustainable presence of the long‐lived **BF_2_bdk**’s S_1_ excited states, making the system more likely to undergo charge separation and thus facilitating the generation of the OLPL afterglow. The robust RISC capability of **BF_2_bdk** is attributed to their modest Δ*E*_ST_ and large SOCME as revealed in **BF_2_bdk**‐PhB systems (vide supra). Therefore, compounds **1** and **2** systems not only exhibit strong ISC abilities but also commendable RISC capabilities (Table 1), which is conducive to the generation of long‐lived **BF_2_bdk**’s S_1_ and T_1_ excited states, making it possible for more excitons to undergo charge separation; this accounts for their superior OLPL afterglow performance compared to other **BF_2_bdk**‐TMB‐PhB samples. For compound **3**, **4**, and **5** systems, all of which possess moderate ISC and RISC abilities (Figure S37, Supporting Information), their OLPL afterglow performance is slightly weaker than that of **1** and **2** systems (Table 1). In contrast, other **BF_2_bdk** systems, such as compounds **6**, **7**, and **8**, exhibit relatively weak RISC capabilities (Figure S37, Supporting Information), which hinder the harvesting of **BF_2_bdk** excitons during charge recombination, resulting in comparatively weaker OLPL afterglow performance (Table 1). To achieve high‐performance OLPL materials, the luminescent molecules need to possess strong ISC capability to increase the population of long‐lived excitons and promote charge separation process. At the same time, they should exhibit RISC capability to harvest both singlet and triplet excitons. For our **BF_2_bdk** molecules, when aromatic rings, especially alkoxy‐substituted aromatic rings, are introduced on both sides, the variety of excited states can be enriched, thereby increasing SOCME and facilitating the ISC process. Additionally, biphenyl groups have a higher T_1_ energy level compared to other common aromatic functional groups such as naphthalene and fluorene functional groups, so the resultant **BF_2_bdk** would show relatively high T_1_ levels and thus have relatively small Δ*E*_ST_. Besides, according to our previous study,^[^ ^45^ ^]^ the T_2_ and T_3_ energy levels of such **BF_2_bdk** compounds with biphenyl groups would be close to their S_1_ level, further facilitating both ISC and RISC processes. Furthermore, cyclizing the alkoxy groups can enhance molecular rigidity, reducing non‐radiative decay pathways. These are the structure‐property relationships in the present OLPL system.

### 2.3 Materials Function

**Figure 5:**
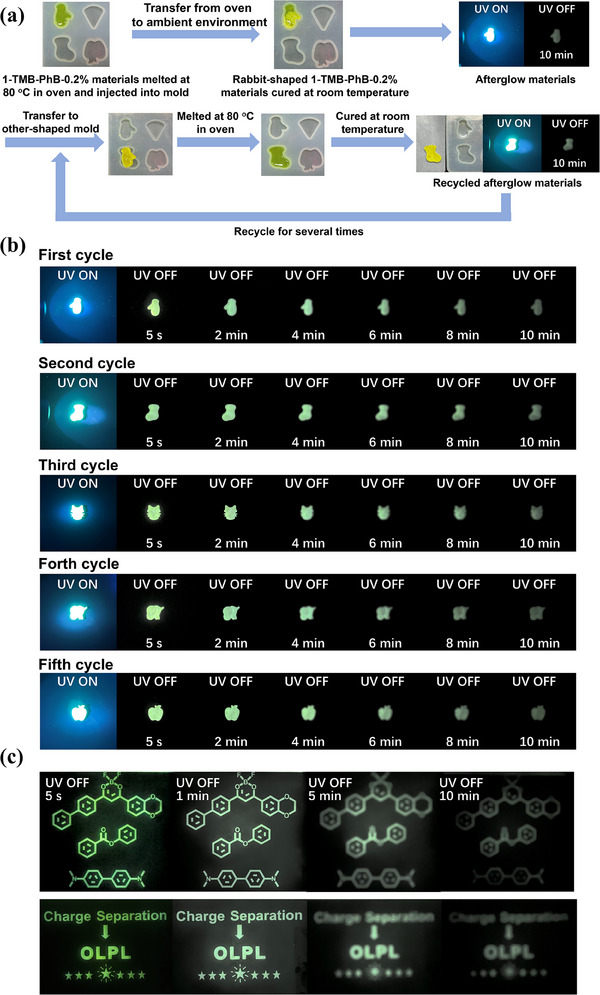
a) A flow chart of preparation of shaped 1‐TMB‐PhB‐0.2% materials and process for recycling of shaped 1‐TMB‐PhB‐0.2% materials. b) Photographs of freshely‐prepared and different‐round recycled 1‐TMB‐PhB‐0.2% materials under a 365 nm UV lamp and after removal of the UV lamp. c) Photographs of 1‐TMB‐PhB‐0.2% afterglow panels covered by patterned PET substrate after removal of 365 nm UV lamp.

The development of advanced luminescent materials has significant implications across various fields, including information technology, security and sustainable manufacturing. As industries increasingly seek innovative solutions for data storage and encryption, materials that combine durability, processability and exceptional photoluminescent properties become critical. In this context, our **BF_2_bdk**‐TMB‐PhB‐0.2% material emerges as a versatile candidate, offering promising functionalities that address these pressing needs. In this work, the **BF_2_bdk**‐TMB‐PhB‐0.2% material showcases remarkable afterglow properties, sustaining luminescence for several hours, an impressive duration that rivals traditional inorganic materials while offering unique advantages in processability. Notably, the **1**‐TMB‐PhB‐0.2% materials exhibit a phase transition from solid to liquid at 80 °C, allowing for effortless molding into various shapes through a simple cooling and curing process at room temperature (**Figure** **5**a). Upon excitation with a UV lamp emitting at 365 nm, this material displays a vivid green afterglow, persisting significantly even after the light source is removed. Specifically, the luminous effect remains evident up to 10 min post‐excitation (Figure 5b). This behavior not only underscores the material's inherent photoluminescent qualities but also illustrates its thermal reformability. After re‐melting at 80 °C and subsequent curing, we observed consistent retention of afterglow intensity across five cycles of this thermal processing (Figure 5b). The OLPL spectra of the **1**‐TMB‐PhB‐0.2% samples exhibit analogous peak shapes and intensities throughout the five cyclic processes (Figure S38a, Supporting Information). Moreover, the decay profiles of the materials from the first cycle and the fifth cycle are nearly identical (Figure S38b, Supporting Information). Such characteristics highlight the material's recyclability and versatility, enabling it to be reshaped with minimal loss in luminescence. We further investigated the stability of the **1**‐TMB‐PhB‐0.2% three‐component material under humid conditions. After exposing the material to a humid environment (75% relative humidity) for 3 and 10 h, the steady‐state and delayed spectra exhibited nearly identical peak shapes and intensities, and the lifetime decay curves displayed comparable profiles, indicating the material's high stability in humidity environments (Figure S39, Supporting Information). We further conducted photophysical measurements on the material subjected to ambient environment for two months and nine months. Remarkably, it show minimal luminescence loss and comparable decay profile to the freshly prepared sample (Figure S38, Supporting Information). This observation strongly implies that our material possesses a considerably long shelf life and excellent stability characteristics. All these experiments support the long‐term stability of our materials. In an innovative application, we encapsulated the **1**‐TMB‐PhB‐0.2% material between two glass plates measuring 10 cm × 10 cm, effectively producing a glow panel that retains a prolonged afterglow duration. Furthermore, when a patterned PET substrate was placed atop this glow panel and exposed to 365 nm radiation for just 15 s, distinct patterns emerged, maintaining notable brightness even after 10 min (Figure 5c). This capability positions our material as a promising candidate for applications in information storage and encryption, opening avenues for novel uses in security and data protection technologies. Through these investigations, we elucidate the multifunctional potential of **BF_2_bdk**‐TMB‐PhB‐0.2%, emphasizing both its luminescent durability and flexibility in application, which could revolutionize the field of smart materials.

## 3 Conclusion

In summary, this work reports a three‐component design strategy for the construction of high‐performance OLPL afterglow system. The **BF_2_bdk**‐TMB‐PhB systems are pioneered by us based on the judicious and intense studies in the field of organic afterglow materials and now this three‐component OLPL system has become the third mechanism, exhibiting advantages of high efficiency and low/normal power switching on compared to the first mechanism donor‐acceptor exciplex OLPL and the second mechanism two‐photon ionization by high‐power excitation. We use our **BF_2_bdk**‐TMB‐PhB model system to deeply investigate the essential steps for high performance OLPL, which is novel and significant for the field. It is known that, in OLPL mechanism, the charge separation and subsequent charge recombination are two essential steps whose efficiency are crucial for material performance. The topics of this study and our previous study^[^ ^61^ ^]^ are very different, one on enhanced charge separation by increased luminophore population and lifetimes, the other on efficient charge recombination by controlling radical cation stability. In this study, by incorporating the electron donor TMB into the **BF_2_bdk**‐PhB system, the obtained **BF_2_bdk**‐TMB‐PhB melt‐cast samples can exhibit significant visible‐light‐excitable OLPL afterglow under ambient conditions, with afterglow duration up to 2 h. These OLPL afterglow materials also show a comparable OLPL afterglow performance to the commercial inorganic materials, with OLPL efficiency of ≈10%. In‐depth investigations reveal that the electron‐donating capability of TMB, as well as the significant population of long‐lived **BF_2_bdk** excited states, are of crucial importance for the enhanced charge separation in the present system. The fs‐TAS experiments further support our proposed process of electron transfer from TMB to **BF_2_bdk** in the charge separation process. Besides, the efficient harvesting of singlet/triplet excitons by **BF_2_bdk**, coupled with the protective environment provided by the crystalline PhB matrix, are also critical to the achievement of the high‐performance OLPL in this work. Given their visible‐light excitation characteristics and hour‐long persistent luminescence, these OLPL materials show promising potential for applications in areas such as information storage and encryption. This research provides a novel design strategy for the construction of high‐performance OLPL systems and explores their possible practical applications. Future research will focus on further optimizing the molecular design to achieve even longer afterglow lifetimes and higher brightness, while exploring the integration of these materials into devices and applications.

## Conflict of Interest

The authors declare no conflict of interest.

## Supporting information



Supporting Information

## Data Availability

The data that support the findings of this study are available from the corresponding author upon reasonable request.
