# Supplementary material for: Boosting Organic Long Persistent Luminescence by Enhancing Charge Separation Processes in Three‐Component Systems
Source: Adv Sci (Weinh). 2025 Jun 30;12(37):e01558. doi: 10.1002/advs.202501558 (PMC12499435; doi:10.1002/advs.202501558)
Supplement: Supplementary file 1 — Supporting Information [file ADVS-12-e01558-s001.pdf]

ADVANCED SCIENCE
Open Access

Supporting Information

for *Adv. Sci.*, DOI 10.1002/advs.202501558

Boosting Organic Long Persistent Luminescence by Enhancing Charge Separation Processes in Three-Component Systems

*Zi Ye, Wen Xia, Guangming Wang, Guoyi Wu, Hongxin Gao, Biao Xu and Kaka Zhang**

Supporting Information

Boosting Organic Long Persistent Luminescence by Enhancing Charge Separation Processes in Three-Component Systems

Zi Ye,[#] Wen Xia,[#] Guangming Wang, Guoyi Wu, Hongxin Gao, Biao Xu, Kaka Zhang*

State Key Laboratory of Organometallic Chemistry and Shanghai Hongkong Joint Laboratory in Chemical Synthesis, Key Laboratory of Synthetic and Self-Assembly Chemistry for Organic Functional Molecules, Ningbo Zhongke Creation Center of New Materials, Shanghai Institute of Organic Chemistry, University of Chinese Academy of Sciences, Chinese Academy of Sciences, 345 Lingling Road, Shanghai 200032, People's Republic of China.

*E-mail: zhangkaka@sioc.ac.cn; [#]equal contribution.

Table of contents

1. Experimental Procedures

1.1 Materials

1.2 Physical mesaurements and instrumentation

1.3 TD-DFT calculations

1.4 Synthesis

1.5 Preparation

2. Supporting Results and Discussion

2.1 Text S1. Ruling other afterglow mechanism for the present BF_2bdk-PhB systems

2.2 Text S2. Estimating room-temperature OLPL efficiency

3. Figures and Tables

1. Experimental Procedures

1.1 Materials

4-Acetylbiphenyl (99%, Admas), 2,3-dihydro-benzo[1,4]dioxine-6-carboxylic acid methyl ester (98%, Bidepharm), 3,4-methylenedioxybenzoate (98%, Bidepharm), 3,4-dimethoxybenzoate (98%, Bidepharm), 2-methoxybenzoate (98%, Bidepharm), 3-methoxybenzoate (99%, Admas), 4-methoxybenzoate (99%, Admas), methyl benzoate (99%, Admas), 4-phenylbenzoate (95%, Bidepharm), sodium hydride (NaH, 60% dispersion in mineral oil, Aladdin), boron trifluoride diethyl etherate (98%, TCI), phenyl benzoate (PhB, 99%, Energy Chemical), N,N,N',N'-tetramethylbenzidine (TMB, 99%, Adamas), 3,4,5,6-tetra(9H-carbazol-9-yl)phthalonitrile (4CzPN, Leyan, 98%), inorganic $Sr_2Al_{14}O_{25}/Eu^{2+}$, Dy^{3+} materials (Yuan Jiangzhi's crystal luminous stone jewelry of Taobao).

1.2 Physical measurements and instrumentation

Nuclear magnetic resonance (NMR) spectra were recorded on a JEOL Fourier-transform NMR spectrometer (400 MHz), including ^{1}H NMR, ^{13}C{^{1}H} NMR, ^{19}F{^{1}H} NMR, ^{11}B{^{1}H} NMR. Mass spectra were performed on Agilent Q-TOF 6520 liquid chromatograph mass spectrometer. UV-vis absorption spectra were recorded on a Techcomp UV1050 and Shimadzu UVmini1285 UV-vis spectrophotometer. The steady-state and delayed emission spectra were collected by Hitachi F-4700 fluorescence spectrometer equipped with chopping systems; the delayed emission spectra were obtained with a delay time of approximately 1 ms. The OLPL spectra were collected by Hitachi F-4700 fluorescence spectrometer using luminescence mode. The decay profiles were collected by Hitachi F-4700 fluorescence spectrometer equipped with chopping systems. The fluorescence decay profiles in nanosecond region were recorded by using time-correlated single photon counting technique (TCSPC) on a Edinburgh FLS1000 fluorescence spectrometer equipped with a picosecond pulsed diode laser. Photoluminescence quantum yield was measured by a Hamamatsu absolute PL quantum yield measurement system based on a standard protocol (*Adv. Mater.* 1997, 9: 230). Electron Spin Resonance (ESR) Spectrum was collected on a Bruker EMX plus 6/1 spectrometer equipped with an Oxford Instrument ESR900 liquid He cryostat using an Oxford ITC 503

temperature controller. The ns-TA experiment was performed using the EDINBURGH-LP980 transient absorption spectrometer, which uses a 290 nm pump laser and a high-energy xenon lamp as the detection light source, generated by a commercial optical parametric amplifier (TOPAS-PRIME). The fs-TA experiment was performed with the laser of Pharos operating at 10 kHz and a TA-100DZ pump/probe setup (Time-Tech Spectra). Photographs and videos were captured by iPhone12 cameras. Before the capture, samples were irradiated by a 365 nm UV lamp (5 W) for approximately 15 s at a distance of approximately 15 cm. HPLC conditions for all compounds mentioned in this text: equipment: WATERS UPLC H CLASSS; column: ACQUITY UPLC BEH C18 1.7 μm, 2.1 × 50 mm; eluent: acetone/water = 100/0; flow rate: 0.6 mL/min.

1.3 TD-DFT calculations

TD-DFT calculations were performed to study the photophysical properties of molecularly dispersed **BF$_2$bdk** in the solid state. Since the afterglow properties are originated from the excited states of molecularly dispersed **BF$_2$bdk** in the crystalline PhB matrices where intermolecular rotation and vibration are largely restricted, the ground-state geometry of **BF$_2$bdk** was used for all the TD-DFT calculations. The ground-state geometry of **BF$_2$bdk** compounds were optimized by a DFT calculation using B3LYP functional and 6-31G(d, p) basis set. The singlet excited states and triplet excited states were calculated on ORCA 4.2.1 program with B3LYP functional and def2-TZVP(-f) basis set and analyzed by Multiwfn software. Spin-orbit coupling (SOC) matrix elements between the singlet excited states and triplet excited states were calculated with spin-orbit mean-field (SOMF) methods on ORCA 4.2.1 program with B3LYP functional and def2-TZVP(-f) basis set. The obtained electronic structures were analyzed by Multiwfn software. All isosurface maps to show the electron distribution and electronic transitions were rendered by Visual Molecular Dynamics (VMD) software based on the exported files from Multiwfn. (Neese F, *Wiley Interdiscip. Rev. Comput. Mol. Sci.* 2018, 8: 1327-1332; Becke AD, *Phys. Rev. A* 1988, 38: 3098-3100; Lee C, Yang W, Parr RG, *Phys. Rev. B.* 1988, 37: 785-789; Miehlich B, Savin A, Stoll H, Preuss H, *Chem. Phys. Lett.* 1989, 157: 200-206; Roy LE, Hay PJ, Martin RL, *J. Chem. Theory Comput.* 2008,

4: 1029–1031; Weigend F, Ahlrichs R, *Phys. Chem. Chem. Phys.* 2005, 7: 3297-3305; Lu T, Chen F, *J. Comput. Chem.* 2012, 33: 580-592; Humphrey W, Dalke A, Schulten K, *J. Mol. Graphics* 1996, 14: 33-38).

1.4 Synthesis

Synthesis of Compound 1

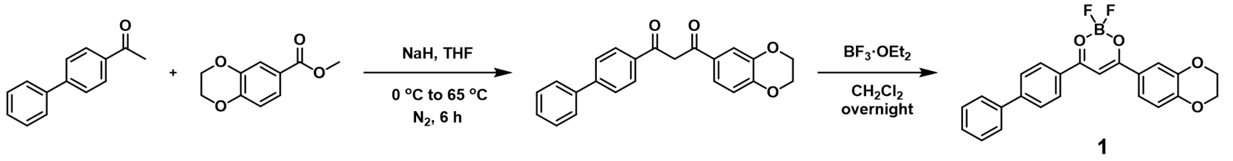

4-Acetylbiphenyl (196 mg, 1 mmol) and anhydrous THF (5.0 mL) were added to a 50 mL round-bottom flask and purged with nitrogen for 10 min. Then NaH (60% oil dispersion, 120 mg, 3 mmol) was added to the resulting solution at 0 °C, and stirred under nitrogen at 0 °C for 30 min, followed by slow addition of 2,3-dihydro-benzo[1,4]dioxine-6-carboxylic acid methyl ester (213 mg, 1.1 mmol) and heated to 65 °C. The resulting mixture was refluxed for 6 hours. The reaction mixture was allowed to cool to room temperature before being quenched carefully with water (10.0 mL) in ice bath. The pH was adjusted to 3 with HCl (aq). The aqueous suspension was extracted with dichloromethane (2 × 30.0 mL). The organic phase was dried over anhydrous sodium sulfate and concentrated under reduced pressure to obtain the crude product. The crude product was directly used for the next step without further purification.

To a solution of the crude product in 10.0 mL dichloromethane, 0.5 mL BF_3·Et_2O was added. The solution was stirred overnight. Water was added to the solution, and the organic layers were collected, washed with water, dried over anhydrous sodium sulfate, and concentrated under reduced pressure. The crude product was purified by column chromatography (petroleum ether/dichloromethane = 1:2) to yield **1** (160 mg, 40%) as a yellow solid. Compound **1** was further purified by recrystallization in spectroscopic grade dichloromethane/hexane. ^{1}H NMR (400 MHz, Chloroform-*d*) δ 8.20 (d, *J* = 8.2 Hz, 2H), 7.76 (d, *J* = 8.4 Hz, 2H), 7.71 (d, *J* = 7.7 Hz, 1H), 7.66 (d, *J* = 7.5 Hz, 3H), 7.56 – 7.36 (m, 4H), 7.24-7.16 (m, 2H), 3.86 (s, 3H). ^{13}C NMR (101 MHz, $CDCl_3$) δ 181.86, 181.14, 150.38, 147.56, 143.90, 139.26, 130.87, 129.29, 129.08, 128.75, 127.60, 127.31, 125.13, 123.55, 118.41, 118.04, 92.66, 77.32, 77.00, 76.68, 64.96, 64.00. ^{19}F NMR (376 MHz, $CDCl_3$, 298K, relative to $CFCl_3$) δ -140.36, -140.43. ^{11}B NMR (128 MHz, Chloroform-d, 298 K, relative to BF_3·Et_2O /ppm) δ 0.34. HRMS m/z found (calcd for $C_{17}H_{15}O_3BF_3$, $[M+H]^+$): 407.1261 (407.1261).

Synthesis of compound 2

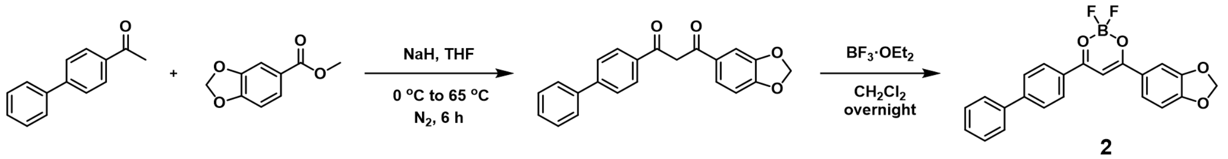

2

4-Acetylbiphenyl (196 mg, 1 mmol) and anhydrous THF (5.0 mL) were added to a 50 mL round-bottom flask and purged with nitrogen for 10 min. Then NaH (60% oil dispersion, 120 mg, 3 mmol) was added to the resulting solution at 0 °C, and stirred under nitrogen at 0 °C for 30 min, followed by slow addition of methyl 3,4-methylenedioxybenzoate (198 mg, 1.1 mmol) and heated to 65 °C. The resulting mixture was refluxed for 6 hours. The reaction mixture was allowed to cool to room temperature before being quenched carefully with water (10.0 mL) in ice bath. The pH was adjusted to 3 with HCl (aq). The aqueous suspension was extracted with dichloromethane (2 × 30.0 mL). The organic phase was dried over anhydrous sodium sulfate and concentrated under reduced pressure to obtain the crude product. The crude product was directly used for the next step without further purification.

To a solution of the crude product in 10.0 mL dichloromethane, 0.5 mL $BF_3·Et_2O$ was added. The solution was stirred overnight. Water was added to the solution, and the organic layers were collected, washed with water, dried over anhydrous sodium sulfate, and concentrated under reduced pressure. The crude product was purified by column chromatography (petroleum ether/dichloromethane = 1:4) to yield **2** (95 mg, 24%) as a yellow solid. The compound **2** was further purified by recrystallization in spectroscopic grade dichloromethane/hexane. ^{1}H NMR (400 MHz, Chloroform-*d*) δ 8.21 (d, *J* = 8.6 Hz, 2H), 7.84 (dd, *J* = 8.3, 1.9 Hz, 1H), 7.78 (d, *J* = 8.6 Hz, 2H), 7.67 (d, *J* = 6.9 Hz, 2H), 7.61 (d, *J* = 1.9 Hz, 1H), 7.53 – 7.42 (m, 3H), 7.10 (s, 1H), 6.97 (d, *J* = 8.3 Hz, 1H), 6.14 (s, 2H). ^{19}F NMR (376 MHz, $CDCl_3$, 298K, relative to $CFCl_3$) δ -140.46, -140.52. Because of its low solubility, we didn't obtain satisfactory ^{13}C and ^{11}B NMR spectra. HRMS m/z found (calcd for $C_{22}H_{15}BFO_4$ $[M-F]^+$, calcd for $C_{22}H_{15}BF_2NaO_4$ $[M+Na]^+$): 373.1068 (373.1042), 415.0941 (415.0924).

Synthesis of compound 3

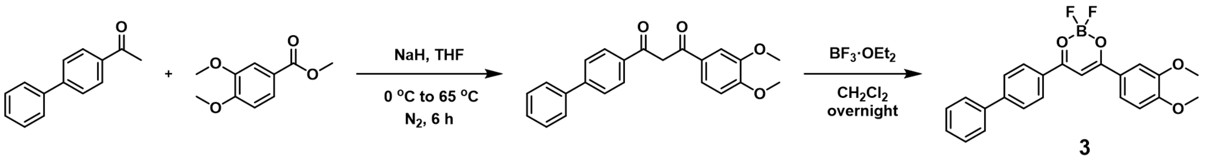

4-Acetylbiphenyl (196 mg, 1 mmol) and anhydrous THF (5.0 mL) were added to a 50 mL round-bottom flask and purged with nitrogen for 10 min. Then NaH (60% oil dispersion, 120 mg, 3 mmol) was added to the resulting solution at 0 °C, and stirred under nitrogen at 0 °C for 30 min, followed by slow addition of methyl 3,4-dimethoxybenzoate (215 mg, 1.1 mmol) and heated to 65 °C. The resulting mixture was refluxed for 6 hours. The reaction mixture was allowed to cool to room temperature before being quenched carefully with water (10.0 mL) in ice bath. The pH was adjusted to 3 with HCl (aq). The aqueous suspension was extracted with dichloromethane (2 × 30.0 mL). The organic phase was dried over anhydrous sodium sulfate and concentrated under reduced pressure to obtain the crude product. The crude product was directly used for the next step without further purification.

To a solution of the crude product in 10.0 mL dichloromethane, 0.5 mL $BF_3{\cdot}Et_2O$ was added. The solution was stirred overnight. Water was added to the solution, and the organic layers were collected, washed with water, dried over anhydrous sodium sulfate, and concentrated under reduced pressure. The crude product was purified by column chromatography (petroleum ether/dichloromethane = 1:1) to yield **3** (207 mg, 51%) as a yellow solid. The compound **3** was further purified by recrystallization in spectroscopic grade dichloromethane/hexane. ^{1}H NMR (400 MHz, Chloroform-*d*) δ 8.15 (d, *J* = 8.0 Hz, 2H), 7.80 (d, *J* = 8.5 Hz, 1H), 7.72 (d, *J* = 8.1 Hz, 2H), 7.67-7.55 (m, 3H), 7.54-7.38 (m, 3H), 7.13 (s, 1H), 6.96 (d, *J* = 8.5 Hz, 1H), 3.98 (d, *J* = 11.0 Hz, 6H). ^{13}C NMR (101 MHz, $CDCl_3$) δ 181.82, 180.63, 155.55, 149.42, 147.42, 139.20, 130.80, 129.21, 129.07, 128.75, 127.50, 127.26, 124.45, 124.37, 110.77, 110.71, 92.55, 56.32, 56.20. ^{19}F NMR (376 MHz, $CDCl_3$, 298K, relative to $CFCl_3$) δ -140.18, -140.24. ^{11}B NMR (128 MHz, Chloroform-d, 298 K, relative to $BF_3{\cdot}Et_2O$ /ppm) δ 0.40. HRMS m/z found (calcd for $C_{23}H_{19}BFO_4$ $[M-F]^+$, calcd for $C_{23}H_{19}BF_2NaO_4$ $[M+Na]^+$): 389.1369 (389.1355), 431.1261 (431.1237)

Synthesis of compound 4

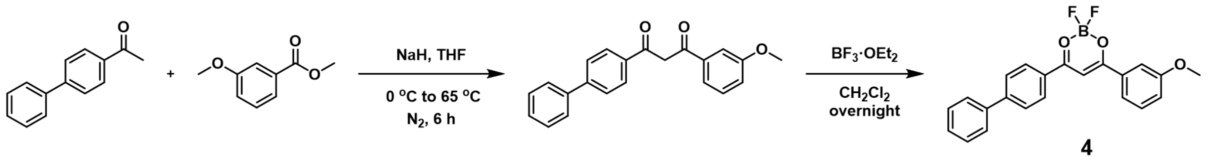

4-Acetylbiphenyl (196 mg, 1 mmol) and anhydrous THF (5.0 mL) were added to a 50 mL round-bottom flask and purged with nitrogen for 10 min. Then NaH (60% oil dispersion, 120 mg, 3 mmol) was added to the resulting solution at 0 °C, and stirred under nitrogen at 0 °C for 30 min, followed by slow addition of methyl 3-methoxybenzoate (182 mg, 1.1 mmol) and heated to 65 °C. The resulting mixture was refluxed for 6 hours. The reaction mixture was allowed to cool to room temperature before being quenched carefully with water (10.0 mL) in ice bath. The pH was adjusted to 3 with HCl (aq). The aqueous suspension was extracted with dichloromethane (2 × 30.0 mL). The organic phase was dried over anhydrous sodium sulfate and concentrated under reduced pressure to obtain the crude product. The crude product was directly used for the next step without further purification.

To a solution of the crude product in 10.0 mL dichloromethane, 0.5 mL $BF_3 \cdot Et_2O$ was added. The solution was stirred overnight. Water was added to the solution, and the organic layers were collected, washed with water, dried over anhydrous sodium sulfate, and concentrated under reduced pressure. The crude product was purified by column chromatography (petroleum ether/dichloromethane = 1:2) to yield **4** (101 mg, 27%) as a yellow solid. The compound **4** was further purified by recrystallization in spectroscopic grade dichloromethane/hexane. ^{1}H NMR (400 MHz, Chloroform-*d*) δ 8.20 (d, J = 8.2 Hz, 2H), 7.76 (d, J = 8.4 Hz, 2H), 7.71 (d, J = 7.7 Hz, 1H), 7.66 (d, J = 7.5 Hz, 3H), 7.56 – 7.36 (m, 4H), 7.24-7.16 (m, 2H), 3.86 (s, 3H). ^{13}C NMR (101 MHz, $CDCl_3$) δ 182.66, 182.57, 160.12, 148.05, 139.08, 133.34, 130.49, 130.06, 129.57, 129.10, 128.89, 127.65, 127.30, 121.77, 121.39, 112.99, 93.55, 55.62. ^{19}F NMR (376 MHz, $CDCl_3$, 298K, relative to $CFCl_3$) δ -139.41, -139.47. ^{11}B NMR (128 MHz, Chloroform-d, 298 K, relative to $BF_3 \cdot Et_2O$ /ppm) δ 0.36. HRMS m/z found (calcd for $C_{22}H_{17}BFO_3$ $[M-F]^+$, calcd for $C_{22}H_{17}BF_2NaO_3$ $[M+Na]^+$): 359.1264 (359.1249), 401.1141 (401.1131).

Synthesis of compound 5

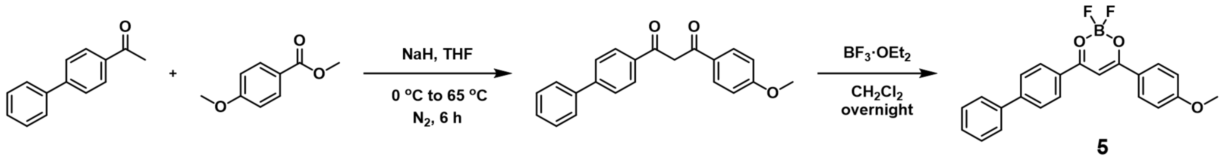

5

4-Acetylbiphenyl (196 mg, 1 mmol) and anhydrous THF (5.0 mL) were added to a 50 mL round-bottom flask and purged with nitrogen for 10 min. Then NaH (60% oil dispersion, 120 mg, 3 mmol) was added to the resulting solution at 0 °C, and stirred under nitrogen at 0 °C for 30 min, followed by slow addition of methyl 4-methoxybenzoate (182 mg, 1.1 mmol) and heated to 65 °C. The resulting mixture was refluxed for 6 hours. The reaction mixture was allowed to cool to room temperature before being quenched carefully with water (10.0 mL) in ice bath. The pH was adjusted to 3 with HCl (aq). The aqueous suspension was extracted with dichloromethane (2 × 30.0 mL). The organic phase was dried over anhydrous sodium sulfate and concentrated under reduced pressure to obtain the crude product. The crude product was directly used for the next step without further purification.

To a solution of the crude product in 10.0 mL dichloromethane, 0.5 mL $BF_3 \cdot Et_2O$ was added. The solution was stirred overnight. Water was added to the solution, and the organic layers were collected, washed with water, dried over anhydrous sodium sulfate, and concentrated under reduced pressure. The crude product was purified by column chromatography (petroleum ether/dichloromethane = 1:2) to yield **5** (96 mg, 25%) as a green solid. The compound **5** was further purified by recrystallization in spectroscopic grade dichloromethane/hexane. ^{1}H NMR (400 MHz, Chloroform-*d*) δ 8.18 (dd, J = 13.3, 8.6 Hz, 4H), 7.77 (d, J = 8.3 Hz, 2H), 7.67 (d, J = 7.1 Hz, 2H), 7.56-7.38 (m, 3H), 7.14 (s, 1H), 7.04 (d, J = 9.1 Hz, 2H), 3.94 (s, 3H). ^{13}C NMR (101 MHz, $CDCl_3$) δ 181.96, 181.00, 165.72, 147.51, 139.30, 131.61, 130.97, 129.25, 129.09, 128.75, 127.61, 127.32, 124.28, 114.68, 92.42, 55.80. ^{19}F NMR (376 MHz, $CDCl_3$, 298K, relative to $CFCl_3$) δ -140.43, -140.49. ^{11}B NMR (128 MHz, Chloroform-d, 298 K, relative to $BF_3 \cdot Et_2O$ /ppm) δ 0.32. HRMS m/z found (calcd for $C_{22}H_{17}BFO_3$ $[M-F]^+$, calcd for $C_{22}H_{17}BF_2NaO_3$ $[M+Na]^+$): 359.1264 (359.1249), 401.1141 (401.1131).

Synthesis of compound 6

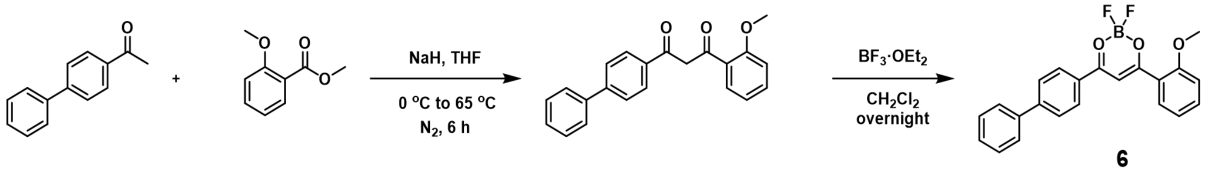

4-Acetylbiphenyl (196 mg, 1 mmol) and anhydrous THF (5.0 mL) were added to a 50 mL round-bottom flask and purged with nitrogen for 10 min. Then NaH (60% oil dispersion, 120 mg, 3 mmol) was added to the resulting solution at 0 °C, and stirred under nitrogen at 0 °C for 30 min, followed by slow addition of methyl 2-methoxybenzoate (182 mg, 1.1 mmol) and heated to 65 °C. The resulting mixture was refluxed for 6 hours. The reaction mixture was allowed to cool to room temperature before being quenched carefully with water (10.0 mL) in ice bath. The pH was adjusted to 3 with HCl (aq). The aqueous suspension was extracted with dichloromethane (2 × 30.0 mL). The organic phase was dried over anhydrous sodium sulfate and concentrated under reduced pressure to obtain the crude product. The crude product was directly used for the next step without further purification.

To a solution of the crude product in 10.0 mL dichloromethane, 0.5 mL $BF_3 \cdot Et_2O$ was added. The solution was stirred overnight. Water was added to the solution, and the organic layers were collected, washed with water, dried over anhydrous sodium sulfate, and concentrated under reduced pressure. The crude product was purified by column chromatography (petroleum ether/dichloromethane = 1:2) to yield **6** (103 mg, 27%) as a yellow solid. The compound **6** was further purified by recrystallization in spectroscopic grade dichloromethane/hexane. ^{1}H NMR (400 MHz, Chloroform-*d*) δ 8.25 (dd, *J* = 8.0, 1.8 Hz, 1H), 8.19 (d, *J* = 8.5 Hz, 2H), 7.77 (d, *J* = 8.7 Hz, 3H), 7.70 – 7.58 (m, 3H), 7.47 (dt, *J* = 25.2, 7.1 Hz, 3H), 7.12 (t, *J* = 7.6 Hz, 1H), 7.06 (d, *J* = 8.4 Hz, 1H), 4.06 (s, 3H). ^{13}C NMR (101 MHz, $CDCl_3$) δ 182.23, 180.64, 160.76, 147.60, 139.31, 136.35, 132.05, 131.14, 129.54, 129.09, 128.75, 127.61, 127.30, 121.33, 121.12, 111.92, 98.62, 56.10. ^{19}F NMR (376 MHz, $CDCl_3$, 298K, relative to $CFCl_3$) δ -139.87, -139.93. ^{11}B NMR (128 MHz, Chloroform-d, 298 K, relative to $BF_3 \cdot Et_2O$ /ppm) δ 0.29. HRMS m/z found (calcd for $C_{22}H_{17}BFO_3$ $[M-F]^+$, calcd for $C_{22}H_{17}BF_2NaO_3$ $[M+Na]^+$): 359.1264 (359.1249), 401.1141 (401.1131).

Synthesis of compound 7

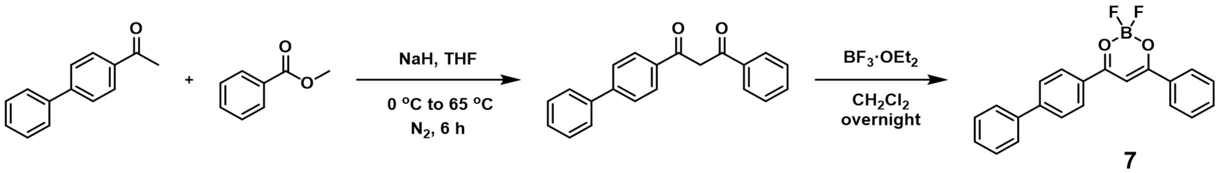

7

4-Acetylbiphenyl (196 mg, 1 mmol) and anhydrous THF (5.0 mL) were added to a 50 mL round-bottom flask and purged with nitrogen for 10 min. Then NaH (60% oil dispersion, 120 mg, 3 mmol) was added to the resulting solution at 0 °C, and stirred under nitrogen at 0 °C for 30 min, followed by slow addition of methoxybenzoate (150 mg, 1.1 mmol) and heated to 65 °C. The resulting mixture was refluxed for 6 hours. The reaction mixture was allowed to cool to room temperature before being quenched carefully with water (10.0 mL) in ice bath. The pH was adjusted to 3 with HCl (aq). The aqueous suspension was extracted with dichloromethane (2 × 30.0 mL). The organic phase was dried over anhydrous sodium sulfate and concentrated under reduced pressure to obtain the crude product. The crude product was directly used for the next step without further purification.

To a solution of the crude product in 10.0 mL dichloromethane, 0.5 mL $BF_3·Et_2O$ was added. The solution was stirred overnight. Water was added to the solution, and the organic layers were collected, washed with water, dried over anhydrous sodium sulfate, and concentrated under reduced pressure. The crude product was purified by column chromatography (petroleum ether/dichloromethane = 1:2) to yield **7** (91 mg, 26%) as a green solid. The compound **7** was further purified by recrystallization in spectroscopic grade dichloromethane/hexane. ^{1}H NMR (400 MHz, Chloroform-*d*) δ 8.19 (dd, *J* = 24.9, 7.8 Hz, 4H), 7.78 (d, *J* = 8.5 Hz, 2H), 7.68 (m, 3H), 7.51 (m, 5H), 7.23 (s, 1H). ^{13}C NMR (101 MHz, $CDCl_3$) δ 182.83, 182.63, 148.03, 139.08, 135.18, 131.98, 130.50, 129.56, 129.15, 129.10, 128.88, 127.64, 127.30, 93.33. ^{19}F NMR (376 MHz, $CDCl_3$, 298K, relative to $CFCl_3$) δ -139.71, -139.77. ^{11}B NMR (128 MHz, Chloroform-d, 298 K, relative to $BF_3·Et_2O$ /ppm) δ 0.40. HRMS m/z found (calcd for $C_{21}H_{15}BFO_2$ $[M-F]^+$, calcd for $C_{21}H_{15}BF_2NaO_2$ $[M+Na]^+$): 329.1158 (329.1149), 371.1041 (371.1025).

Synthesis of compound 8

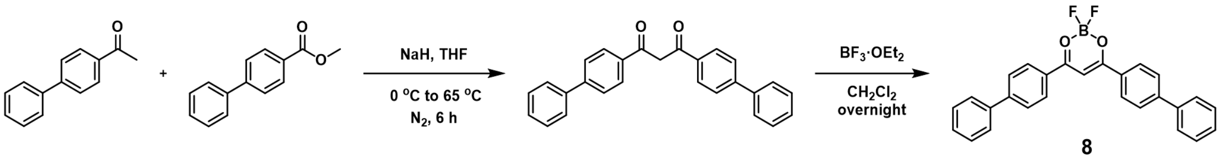

8

4-Acetylbiphenyl (196 mg, 1 mmol) and anhydrous THF (5.0 mL) were added to a 50 mL round-bottom flask and purged with nitrogen for 10 min. Then NaH (60% oil dispersion, 120 mg, 3 mmol) was added to the resulting solution at 0 °C, and stirred under nitrogen at 0 °C for 30 min, followed by slow addition of methyl 4-phenylbenzoate (254 mg, 1.2 mmol) and heated to 65 °C. The resulting mixture was refluxed for 6 hours. The reaction mixture was allowed to cool to room temperature before being quenched carefully with water (10.0 mL) in ice bath. The pH was adjusted to 3 with HCl (aq). The aqueous suspension was extracted with dichloromethane (2 × 30.0 mL). The organic phase was dried over anhydrous sodium sulfate and concentrated under reduced pressure to obtain the crude product. The crude product was directly used for the next step without further purification.

To a solution of the crude product in 10.0 mL dichloromethane, 0.5 mL $BF_3{\cdot}Et_2O$ was added. The solution was stirred overnight. Water was added to the solution, and the organic layers were collected, washed with water, dried over anhydrous sodium sulfate, and concentrated under reduced pressure. The crude product was purified by column chromatography (petroleum ether/dichloromethane = 1:1) to yield **8** (153 mg, 36%) as a green solid. The compound **8** was further purified by recrystallization in spectroscopic grade dichloromethane/hexane. ^{1}H NMR (400 MHz, Chloroform-*d*) δ 8.25 (d, J = 8.6 Hz, 4H), 7.80 (d, J = 8.5 Hz, 4H), 7.68 (d, J = 6.9 Hz, 4H), 7.58 – 7.37 (m, 6H), 7.27 (s, 1H). ^{13}C NMR (101 MHz, $CDCl_3$) δ 182.36, 148.02, 139.18, 130.69, 129.56, 129.12, 128.88, 127.71, 127.34, 93.29. ^{19}F NMR (376 MHz, $CDCl_3$, 298K, relative to $CFCl_3$) δ -139.88, -139.96. ^{11}B NMR (128 MHz, Chloroform-d, 298 K, relative to $BF_3{\cdot}Et_2O$ /ppm) δ 0.46. HRMS m/z found (calcd for $C_{27}H_{19}BFO_2$ $[M-F]^+$, calcd for $C_{27}H_{19}BF_2NaO_2$ $[M+Na]^+$): 405.1450 (405.1457), 447.1337 (447.1338).

1.5 Preparation

Preparation of 1-TMB-PhB-0.2% afterglow materials

Before preparation, **1-8 and TMB** were purified *via* recrystallization in spectroscopic grade dichloromethane/*n*-hexane. For the preparation of **1**-PhB-TMB-0.2% afterglow materials, 400 μL **1** in dichloromethane (1 mg/mL), 200 μL TMB in dichloromethane (2 mg/mL) and 200 mg phenyl benzoate (PhB) were added into a 3 mL sample bottle, and then heated to 80 °C to evaporate organic solvent and form a molten mixture. After the solid is completely melted, the sample bottle is transferred to room temperature for cooling and crystallization naturally, then melt-cast **1**-TMB-PhB-0.2% afterglow materials were obtained. Other three-component afterglow materials were prepared by the same procedures. For the preparation of **1**-TMB-PhB-0.2% film, the molten mixture of **1**, PhB and TMB was first cast onto a 1 cm ×1 cm quartz plate and then immediately covered by another 1 cm ×1 cm quartz plate. The sandwiched molten mixture formed a thin film after cooling.

Preparation of TMB-PhB and TMB-PhB-fluorescent dopant materials

For the preparation of TMB-PhB-0.2% afterglow materials, 200 μL TMB in dichloromethane (2 mg/mL) and 200 mg PhB were added into a 3 mL sample bottle, and then heated to 80 °C to evaporate organic solvent and form a molten mixture. After the solid is completely melted, the sample bottle is transferred to room temperature for cooling and crystallization naturally, then melt-cast TMB-PhB-0.2% afterglow materials were obtained. 4CzPN-TMB-PhB-0.2% and NBN-TMB-PhB-0.2% materials were prepared through similar processes, except that an additional 0.4 mg 4CzPN or NBN (chemical structures can be found in the following) was added.

Preparation of TMB-COP-0.2% film

For the preparation of TMB-COP-0.2% materials, 200 μL TMB in dichloromethane (2 mg/mL) and 100 mg COP in 2 mL cyclohexane (50 mg/mL) were added into a 3 mL sample bottle. After homogeneous dissolution, the solution was slowly evaporated on a quartz sheet to obtain the TMB-COP-0.2% transparent film.

Preparation of 1-PhB-0.02% afterglow materials

For the preparation of **1**-PhB-0.02% afterglow materials, 40 μL **1** in dichloromethane (1 mg/mL) and 200 mg PhB were added into a 3 mL sample bottle, and then heated to 80 °C to evaporate organic solvent and form a molten mixture. After the solid is completely melted, the sample bottle is transferred to room temperature for cooling and crystallization naturally, then melt-cast **1**-PhB-0.02% afterglow materials were obtained. Other BF_2bdk-PhB-0.02% afterglow materials were prepared as the same processes.

2. Supporting Results and Discussion

Text S1. Ruling other afterglow mechanism for the present BF_2bdk-PhB systems

Room-temperature afterglow can be generated due to the energy transfer from room-temperature phosphorescent (RTP) donors to luminescent acceptors (*Nat. Commun.* 2020, *11*, 4802; *Angew. Chem. Int. Ed.* 2020, *59*, 9393); this energy transfer process necessitates sufficient excitation of the RTP donors. In the present study, we obtained BF_2bdk-PhB afterglow materials that can be effectively excited using both 365 nm UV lamp and 405 nm light irradiation. Notably, the PhB matrices exhibit minimal UV-visible absorption at these wavelengths. Therefore, it is reasonable to dismiss the hypothesis that room-temperature afterglow in this system arises from energy transfer from the PhB matrices to BF_2bdk dopants.

Recent investigations have indicated that the triplet excited states of organic matrices may facilitate singlet-to-triplet intersystem crossing (ISC) of luminescent dopants, provided the T_1 energy levels of the matrices fall within the S_1 and T_1 states of the dopants. This mechanism has been implicated in the realization of organic room-temperature afterglow (*Angew. Chem. Int. Ed.* 2020, *59*, 16054). However, this ISC mediation is not applicable to the BF_2bdk-PhB system under investigation, as the T_1 level of the PhB matrices (3.53 eV, estimated by TD-B3LYP/6-31g(d,p) calculation) is considerably higher than the S_1 and T_1 states of the BF_2bdk molecules. Furthermore, the elevated T_1 levels of the organic matrices serve a critical role in mitigating afterglow quenching resulting from triplet-to-triplet energy transfers from luminescent dopants to the organic matrices, thereby facilitating the development of

high-performance afterglow materials (*Adv. Funct. Mater.* 2013, *23*, 3386; *Angew. Chem. Int. Ed.* 2021, *60*, 17138).

Within organic materials, a donor-acceptor mechanism can induce charge-separated states, as observed in certain organic systems (*Nature* 2017, *550*, 384). The prolonged luminescence seen in these organic environments is generally attributed to retarded charge recombination occurring within rigid solid matrices. In organic donor-acceptor afterglow systems, the generation of charge-separated states through intermolecular charge transfer between donor and acceptor molecules is essential for achieving long persistent luminescent properties. Nonetheless, our findings indicate negligible intermolecular charge transfer between the BF_2bdk and the PhB matrices, attributable to the latter's lower HOMO and higher LUMO levels. This lack of charge transfer precludes the possibility of long persistent luminescence mechanisms in this system (Scheme1).

Emerging literature suggests that impurities might also contribute to organic room-temperature phosphorescence (*Nat. Mater.* 2021, *20*, 175). In the current study, we meticulously purified the BF_2bdk compounds *via* column chromatography and subjected them to three rounds of recrystallization in spectroscopic-grade dichloromethane/*n*-hexane to ensure their purity. High-performance liquid chromatography (HPLC) analyses corroborate their high purity (Figure S20, S40, S44, S50, S56, S62, S68, S74 and S80). Besides, the high-purity BF_2bdk powders and PhB matrices do not exhibit any room-temperature afterglow characteristics, while the BF_2bdk-PhB afterglow materials, formed by doping BF_2bdk into PhB matrices, demonstrate exceptional afterglow properties under ambient conditions. Remarkably, even at low doping concentrations (e.g., 0.02 wt%), the afterglow materials retain significant afterglow capabilities (Figure S13 and S14). In such low-concentration scenarios, any potential impurities would be spatially segregated from the BF_2bdk molecules by the organic matrix, thus minimizing the statistical likelihood of charge separation and recombination processes (*Nat. Mater.* 2021, *20*, 175) occurring between BF_2bdk and potential impurities. Consequently, the experimental and analytical results presented herein effectively negate the possibility that the observed room-temperature afterglow properties of BF_2bdk-PhB materials stem from impurities.

Text S2. Estimating room-temperature OLPL efficiency

Based on literature survey, one can obtain some background about the estimation of room-temperature OLPL efficiency. In the reported studies where the OLPL materials are sensitive to air (for example, *Nature* **2017**, *550*, 384), the PLQY values of the materials under the protection of nitrogen and exposing to air be measured, respectively, by an instrument equipped with an integrating sphere. The PLQY difference ($PLQY_{nitrogen}$ – $PLQY_{air}$) can be roughly considered as the efficiency of triplet and charge-separation-related component of emission (*Nature* **2017**, *550*, 384). In some reported studies such as *Nat. Commun.* (**2020**, *11*, 191) and *Nat. Mater.* (**2022**, *21*, 338), OLPL efficiency has not been discussed. In other reported studies, for instance, the two-photon ionization OLPL system (*Adv. Mater.* **2020**, *32*, 2003911), OLPL afterglow brightness is not in a linear relationship with excitation intensity. In n-type OLPL and polymer-based OLPL systems, the OLPL intensity would also depend on excitation time (due to the presence of oxygen and other factors). In the aspect of instrument, because OLPL materials usually have minutes/hours-long durations, the PLQY values obtained from integrating sphere measurement systems (such as Hamamatsu absolute PLQY measurement systems) don't involve the contribution of OLPL afterglow. Consequently, the measurement or estimation of OLPL efficiency is not easy, as written in *Nat. Commun.* (**2020**, *11*, 191), "the quantum efficiency of LPL emission is difficult to define, because the charge accumulation and release processes are slow and complicated in contrast to those of long-lived phosphorescence". The **BF_2bdk**-TMB-PhB OLPL materials in the present work are relatively simple, since it can be excited by normal power UVA and visible lamps under ambient conditions. Our OLPL materials are not sensitive to air since the triplets and radical intermediates can be well protected by the crystalline lattice of PhB matrix. Notably, the **1**-TMB-PhB materials can be readily excited by the excitation source of Hitachi F-4700 fluorescence spectrometer to display emission decay profile with two distinct decay modes (Figure 3f in the main text). The proportions of the first-part conventional afterglow and the second-part OLPL afterglow can be calculated to be 51.2% and 48.8%, respectively. Based on related studies (*Nature* **2012**, *492*, 234; *Adv. Mater.* **2013**, *25*, 3707; *Nat. Commun.* **2020**, *11*, 842; *Angew. Chem. Int. Ed.* **2021**, *60*, 17138; *Adv. Mater.* **2024**, 2312439; *J. Am. Chem. Soc.*

2024, *146*, 24871), we conceive that, if one can obtain the efficiency of the first-part conventional afterglow, it is possible to estimate the OLPL efficiency by the emission decay profile and the afterglow proportions (Figure 3f). The PLQY of **1**-TMB-PhB materials at room temperature has been measured to be 20.6% by integrating sphere measurement system. **1**-TMB-PhB materials show fluorescence and phosphorescence bands in the steady-state emission spectra. Based on the literature reported method (for example, the materials in *Adv. Mater*. **2022**, *34*, 2108333; *Adv. Opt. Mater.* **2023**, 2302311; *Adv. Sci.* **2023**, *10*, 2207003; *Nat. Commun*. **2024**, *15*, 3660), to calculate their conventional afterglow efficiency, one can first obtain the percentage of phosphorescence component from the steady-state emission spectra and then the afterglow efficiency is the product of PLQY and phosphorescence percentage. However, the percentage of phosphorescence component is interfered by RISC processes for the **1**-TMB-PhB materials at room temperature, so we use the percentage of phosphorescence component of 44.39% in 77 K steady-state emission spectra (Figure S33a and Table 1) for the calculation. The resultant value of conventional afterglow efficiency is estimated to be 9.1% (20.6% × 44.39%). According to the afterglow proportions in Figure 3f, the OLPL efficiency can be estimated as 8.7% (9.1% × 48.8% / 51.2%). By using the same procedure, the conventional afterglow efficiency of **2**-TMB-PhB materials can be estimated as 13.1% (25.0% × 52.54%, Figure S33b and Table 1), while the OLPL efficiency of **2**-TMB-PhB materials can be estimated to be 11.6% (13.1% × 46.9% / 53.1%, Figure S19d). In the present work, we fully consider the specific property of our **BF_2bdk**-TMB-PhB afterglow materials and propose the above method to estimate OLPL efficiency.

3. Figures and Tables

(a) D-A mechanism

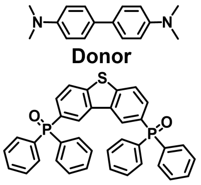

Donor

Acceptor

526 nm, **in N_2**

PLQY = 21%(7% in air), Φ_{OLPL} = 14%

Decay time > 5000 s

Duration time > 1 hour

Nature **2017**, *550,* 384.

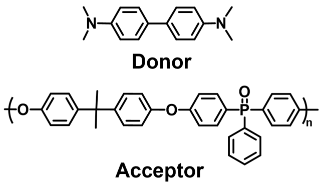

Donor

Acceptor

526 nm, **in N_2**

PLQY = 3%

Decay time ~ 300 s

Duration time > 7 min

Adv. Mater. **2018**, *30*, 1803713.

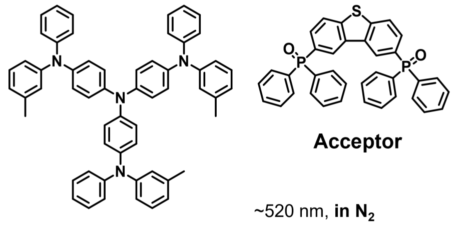

Donor

Acceptor

~520 nm, **in N_2**

PLQY = 31%

Decay time > 1000 s

Duration time = -

Chem. Lett. **2019**, *48*, 270.

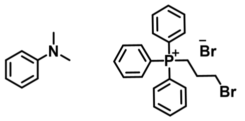

Donor **Acceptor**

500 nm, **under ambient conditions**

PLQY = -

Decay time = 7 hour

Duration time = 7 hour

Adv. Mater. **2020**, *32,* 2001026.

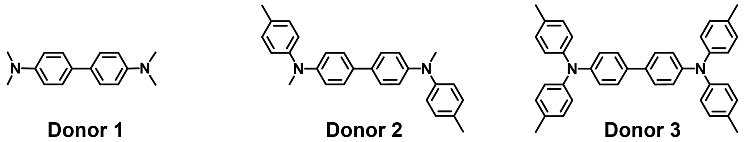

Donor 1 **Donor 2** **Donor 3**

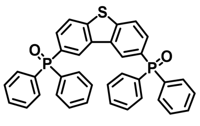

Acceptor

Entry	λ_{OLPL} / nm	PLQY / %	Decay time / s
D1/A	530	24	~1000
D2/A	~540	27	~800
D3/A	~530	28	~400

In N_2 *Nat. Commun.* **2020**, *11,* 191.

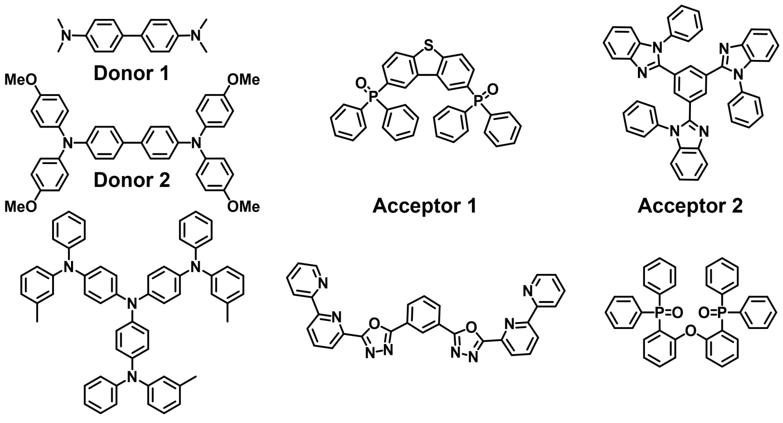

Donor 1

Donor 2

Donor 3

Acceptor 1 **Acceptor 2**

Acceptor 3 **Acceptor 4**

Entry	λ_{OLPL} / nm	PLQY / %	Decay time / s
D1/A1	527	6.5	868
D2/A1	524	9.3	1073
D3/A1	523	35.2	1352
D3/A2	557	16.6	110
D3/A3	587	10.9	47
D3/A4	471	7.6	229

In N_2 *Adv. Funct. Mater.* **2020**, *30*, 2000795.

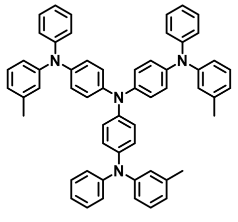

Donor

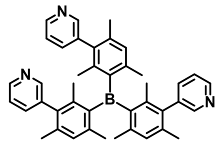

Acceptor

525 nm, **in N_2**

PLQY = -

Decay time ~ 120 s

Duration time > 100 s

Small Struct. **2023**, *4*, 2300052.

(a) D-A mechanism

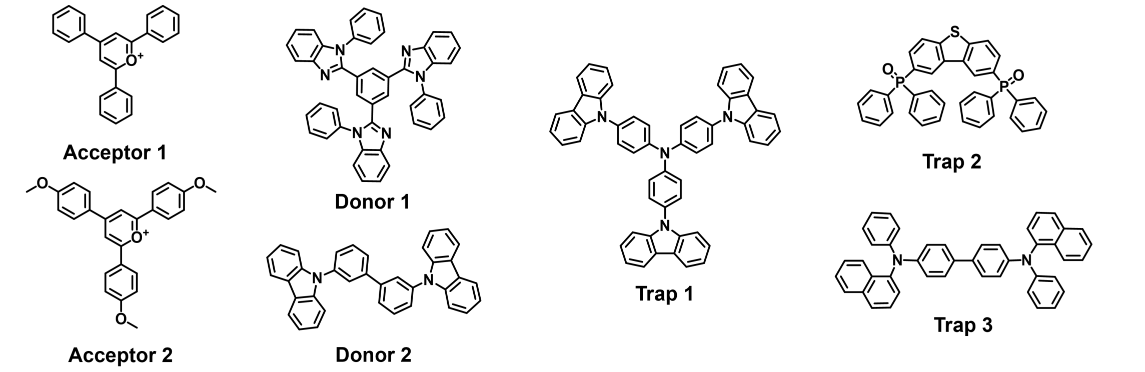

Entry	λ_{OLPL} / nm	PLQY / %	Decay time / s	Decay time in air / s
D1/A1	603	7	1830	160
D2/A1	731	1	33	-
D1/A2	-	1	-	-
D2/A2	624	7	610	-
D1/A1/T1	601	2	14550	1685
D1/A1/T2	597	9	850	415
D1/A1/D2	600	8	970	285
D1/A1/T3	601	1	12640	1060

in N_2, *Nat. Mater.* **2022**, *21,* 338.

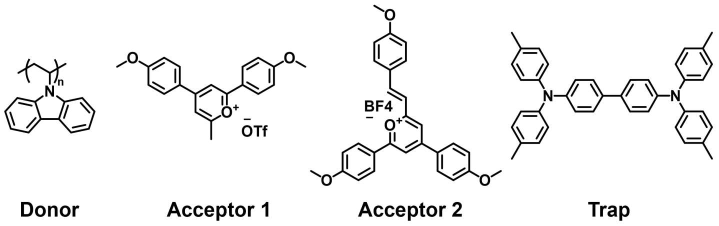

Entry	λ_{OLPL} / nm	PLQY / %	Decay time / s
D/A1	606	-	~5000
D/A2	650	-	~3000
D/A1/T	606	-	>10000

Under ambient conditions

Chem. Sci. **2023**, *14*, 8180.

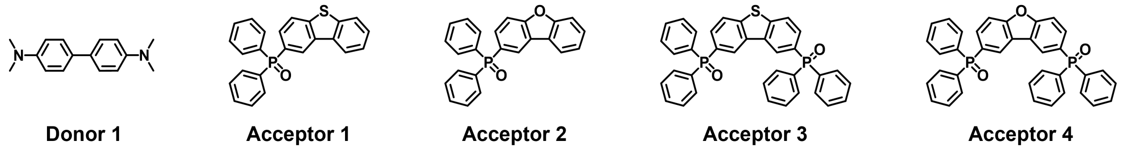

Entry	λ_{OLPL} / nm	PLQY/ %	Decay time / s	Duration time / s	Decay time	Duration time
D/A1	512	1.3	~8000	5163	>180 min	120 min
D/A2	-	6.3	~10	24	High-power excitation	30 min
D/A3	-	2.9	~2500	2017		5 min
D/A4	-	4.2	~100	288		1 min

In N_2 *Sci. China. Mater.* **2023**, *66,* 4756.

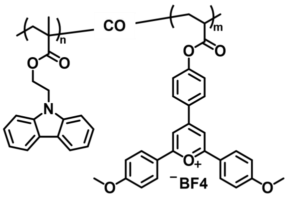

CP1: m/n = 0.003 CP2: m/n = 0.005

CP3: m/n = 0.012 CP4: m/n = 0.090

Entry	λ_{OLPL} / nm	PLQY / %	Decay time	Duration time
CP1	690	3.5	~4000 s	
CP2	698	3.1	~4000 s /~400 s in air	<10 min in air
CP3	714	1.3	~1000 s	
CP4	732	0.7	~10 s	

In N_2 *Angew.Chem. Int.Ed.* **2024**, *63*,e202314500.

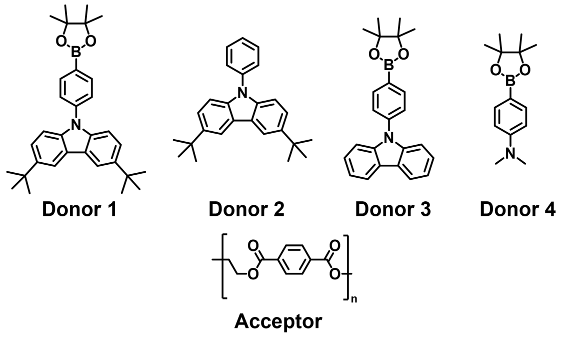

Entry	λ_{OLPL} / nm	Φ_{OLPL} / %	Decay time / s	Duration time / s
D1/A	500	21.3	~10000	39600 = 11 hour
D2/A	500	-	~5000	5000
D3/A	488	-	~3000	3000
D4/A	500	-	~200	200

Under ambient conditions *Adv. Mater.* **2024**, *36*, 2312439.

(b) TPI mechanism

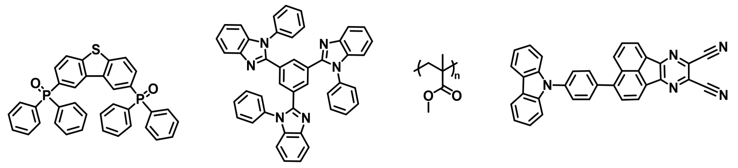

Host 1 | Host 2 | Host 3 | Guest

Entry	λ_{OLPL} / nm	PLQY in air / %	Decay time / s	Duration time / s
H1/G	586	49.9	~4500	>1000
H2/G	578	46.2	~1500	>1000
H3/G	560	29.1	~400	>400

Adv. Mater. **2020**, *32*, 2003911.

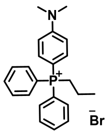

450 nm, **in air**

PLQY = 3.3%

Decay time ~ 20 min

Duration time ~ 12 min

J. Am. Chem. Soc. **2022**, *144*, 3050.

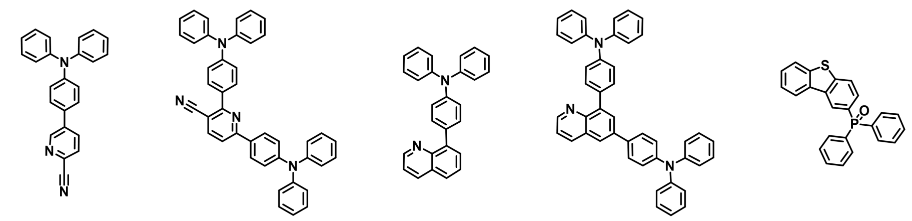

Guest 1 | Guest 2 | Guest 3 | Guest 4 | Host

Entry	λ_{OLPL} / nm	PLQY / %	Decay time / s	Duration time / s
D1/A	532	-	~2400	>1200
D2/A	538	-	~2500	>2400
D3/A	550	-	~3000	>2400
D4/A	577	-	~2300	>1200

Angew. Chem. Int. Ed. **2021**, *60*, 16984.

(c) Other mechanism

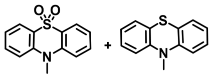

~497 nm, **in air**

$\Phi_{phos.}$ = 20%

Decay time ~ 1600 s

Duration time > 25 min

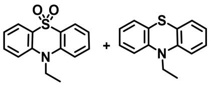

~503 nm, **in air**

$\Phi_{phos.}$ = 43%

Decay time ~ 1400 s

Duration time > 25 min

Adv. Mater. **2021**, *33*, 2007811.

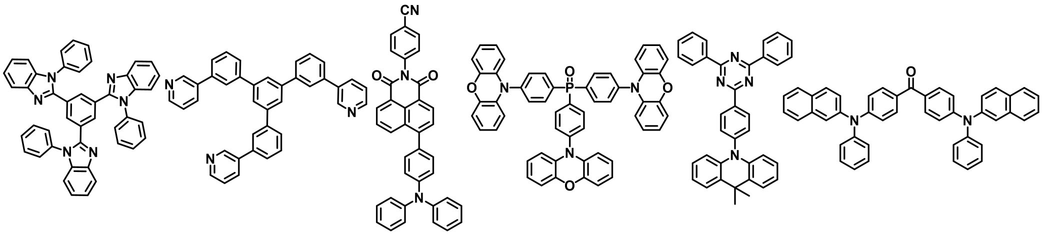

Host 1 | Host 2 | Guest 1 | Guest 2 | Guest 3 | Guest 4

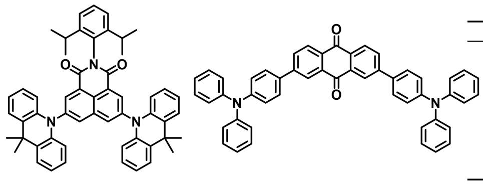

Guest 5 | Guest 6

Entry	λ_{OLPL} / nm	PLQY	Decay time / s	Duration time / h
H1/G1	585	-	~100000	>24
H1/G2	507	-	~4000	1.2
H1/G3	530	-	~5000	2.1
H1/G4	517/552	-	~3500	1.06
H1/G5	640	-	>40000	>12
H1/G6	669	-	~2000	0.86
H2/G1	594	-	~4500	1.6

In N_2 *Nat.Photonics.* **2024**, *18*, 350-356.

Figure S1. Examples of afterglow materials with OLPL property (D-A mechanism: OLPL system based on donor and acceptor pairs; TPI mechanism: OLPL system based on two-photon ionization; other mechanism. Decay time: afterglow duration obtained from emission decay profiles; duration time: afterglow duration obtained by cameras or observed by human eyes).

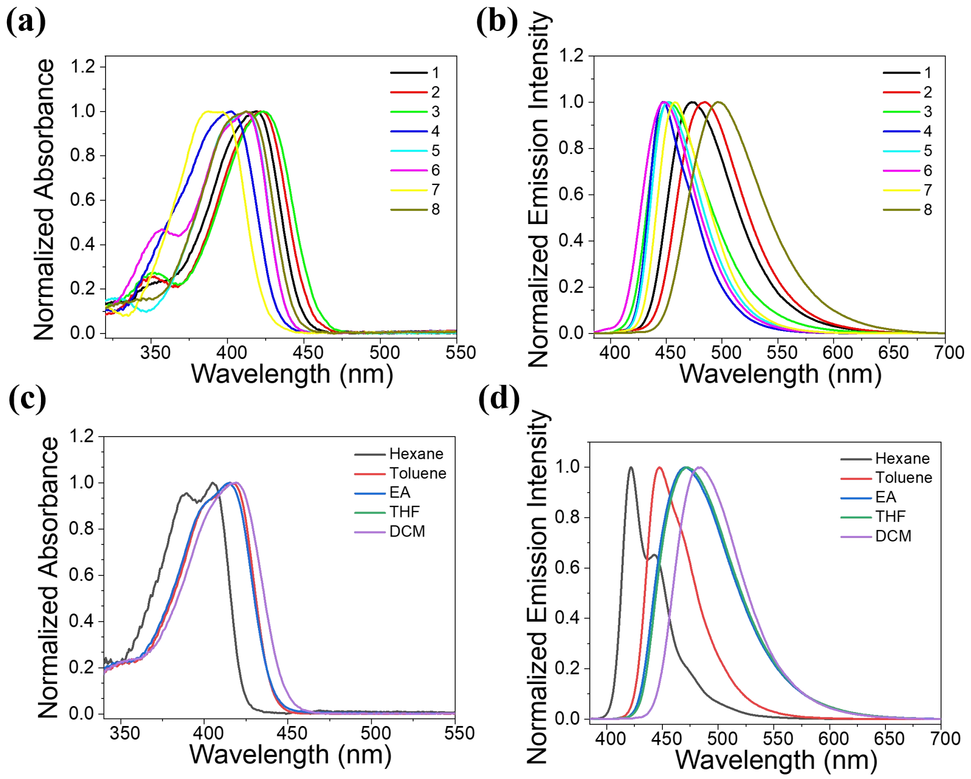

Figure S2. (a) UV-vis absorption spectra and (b) steady-state emission spectra of **1-8** in dichloromethane. UV-vis absorption spectra (c) and steady-state emission spectra (d) of **1** in different solvents. DCM, EA and THF refer to dichloromethane, ethyl acetate and tetrahydrofuran, respectively.

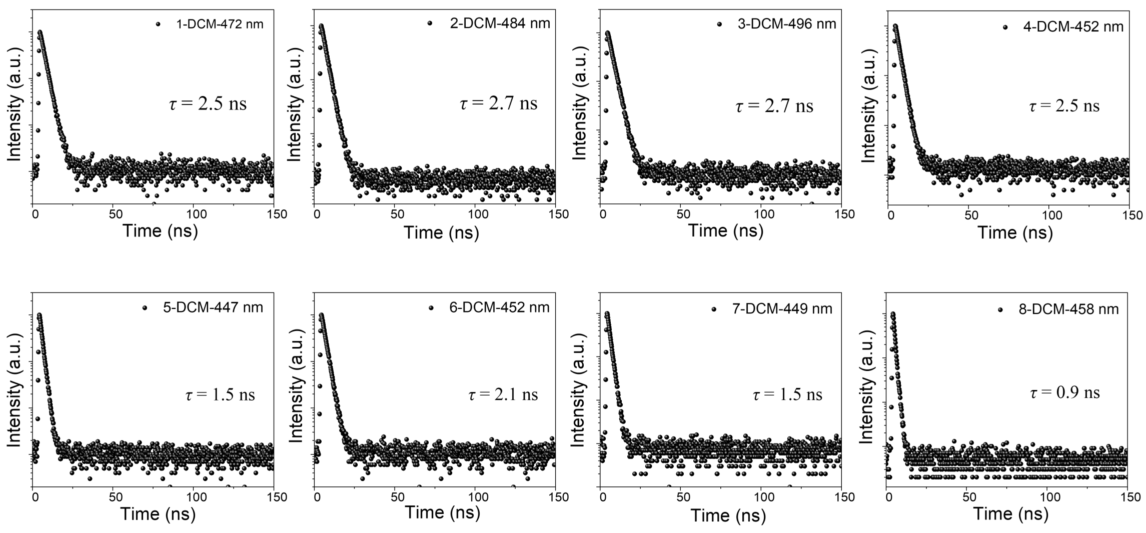

Figure S3. Fluorescence decay profiles of **1-8** in dichloromethane excited at 365 nm.

Table S1. Photophysical property of **1-8** solutions in dichloromethane.

Entry	λ_{abs} / nm	ε × 10^4 / (L mol^{-1} cm^{-1}) at λ_{abs} / $\lambda_{365\ nm}$	λ_F / nm	τ_F / ns	Φ / %
1	419	5.58 / 1.41	472	2.5	47.0
2	421	4.14 / 0.79	484	2.7	47.3
3	423	4.98 / 1.00	496	2.7	44.0
4	412	6.62 / 1.74	447	1.5	85.6
5	403	4.23 / 2.32	452	2.5	30.2
6	412	5.35 / 2.30	452	2.1	68.8
7	397	4.12 / 2.38	449	1.5	72.1
8	412	5.63 / 1.44	458	0.9	53.0

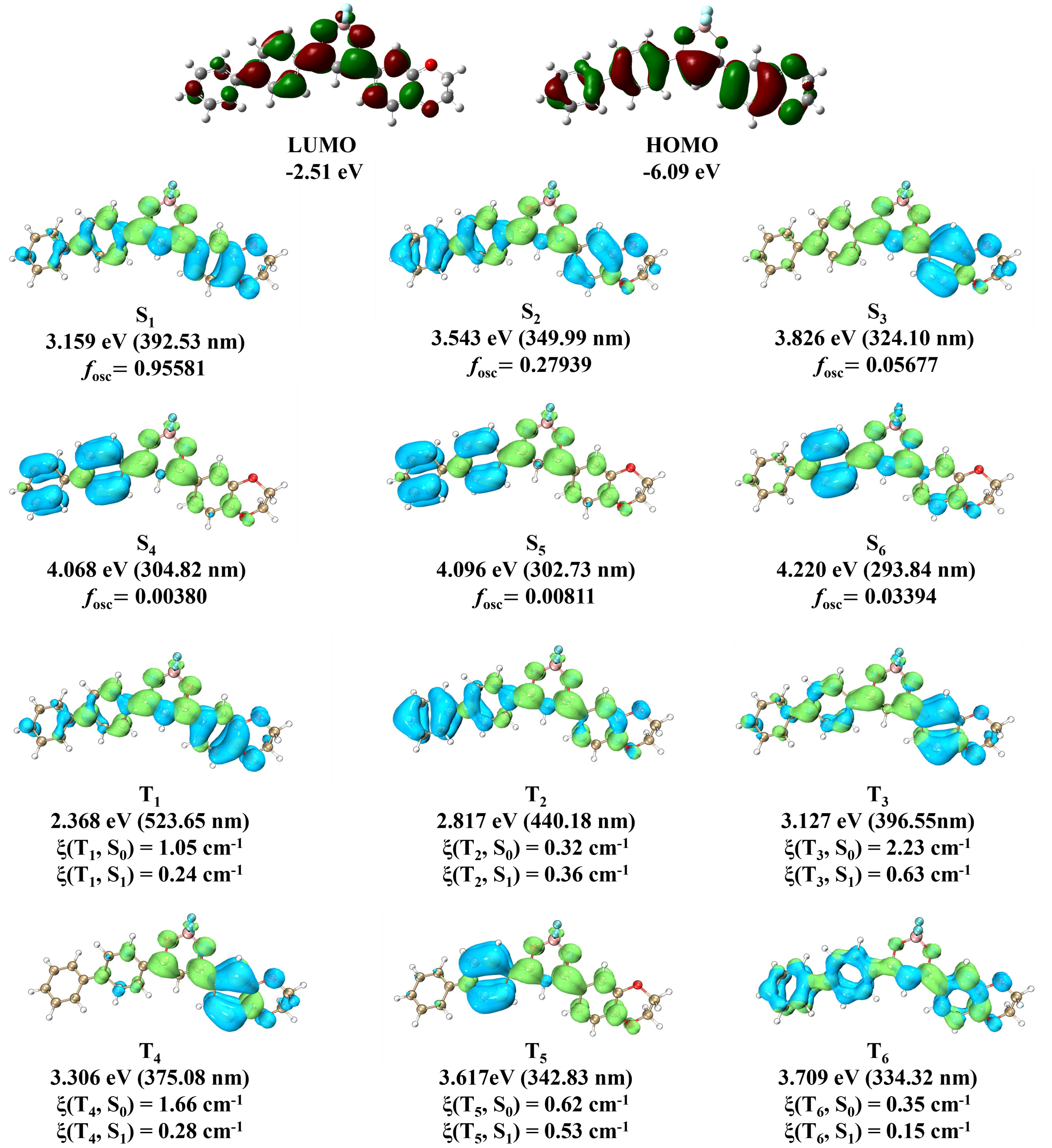

Figure S4. HOMO/LUMO energy levels of **1** calculated at B3LYP/6-31G(d,p) level. Iso-surface maps of electron-hole density difference of **1**'s excited states calculated at B3LYP/def2-TZVP(-f) level, where blue and green iso-surfaces correspond to hole and electron distributions, respectively, and excitation energies, oscillator strengths and spin–orbit coupling matrix element (SOCME) values.

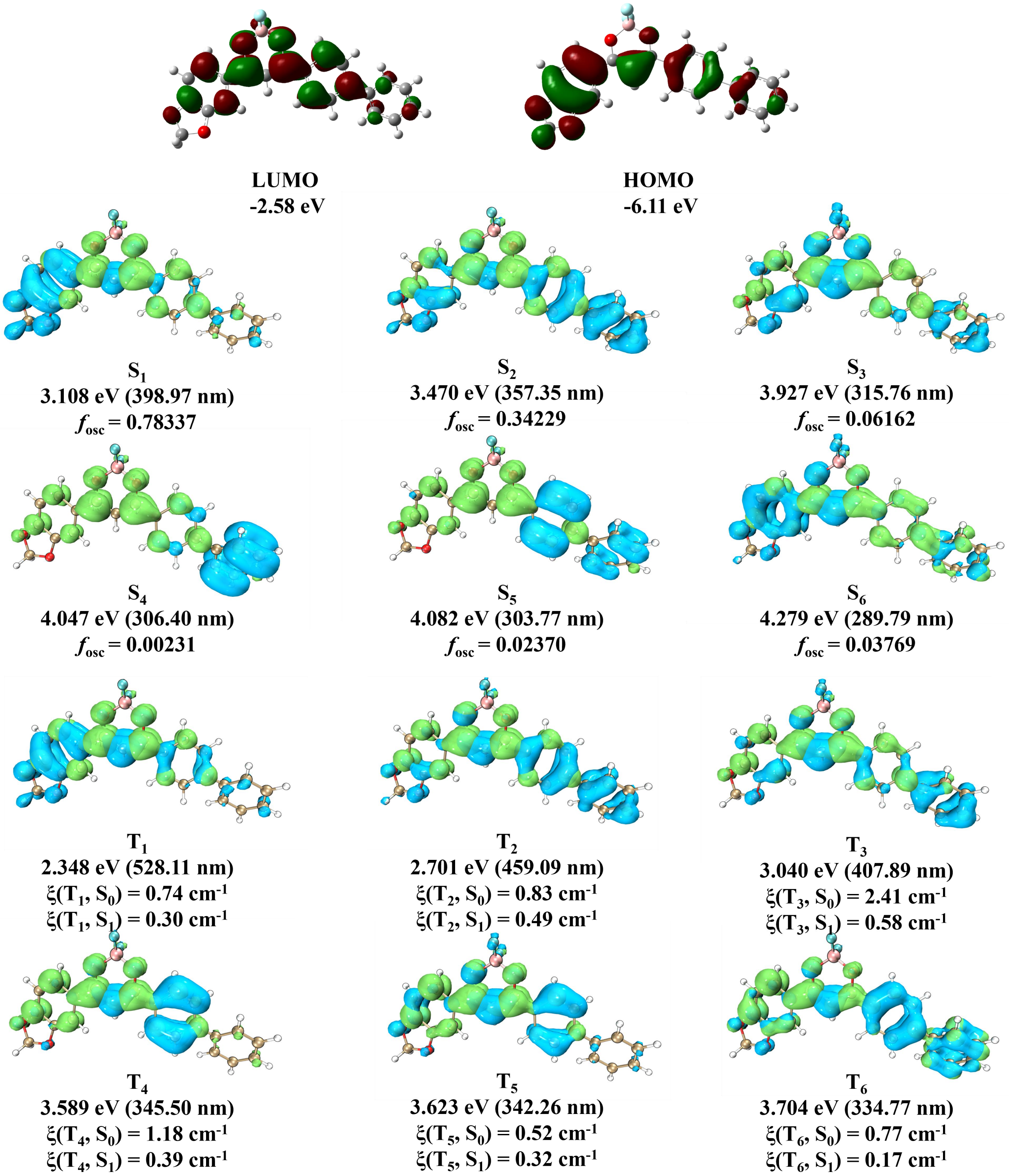

Figure S5. HOMO/LUMO energy levels of **2** calculated at B3LYP/6-31G(d,p) level. Iso-surface maps of electron-hole density difference of **2**'s excited states calculated at B3LYP/def2-TZVP(-f) level, where blue and green iso-surfaces correspond to hole and electron distributions, respectively, and excitation energies, oscillator strengths and spin–orbit coupling matrix element (SOCME) values.

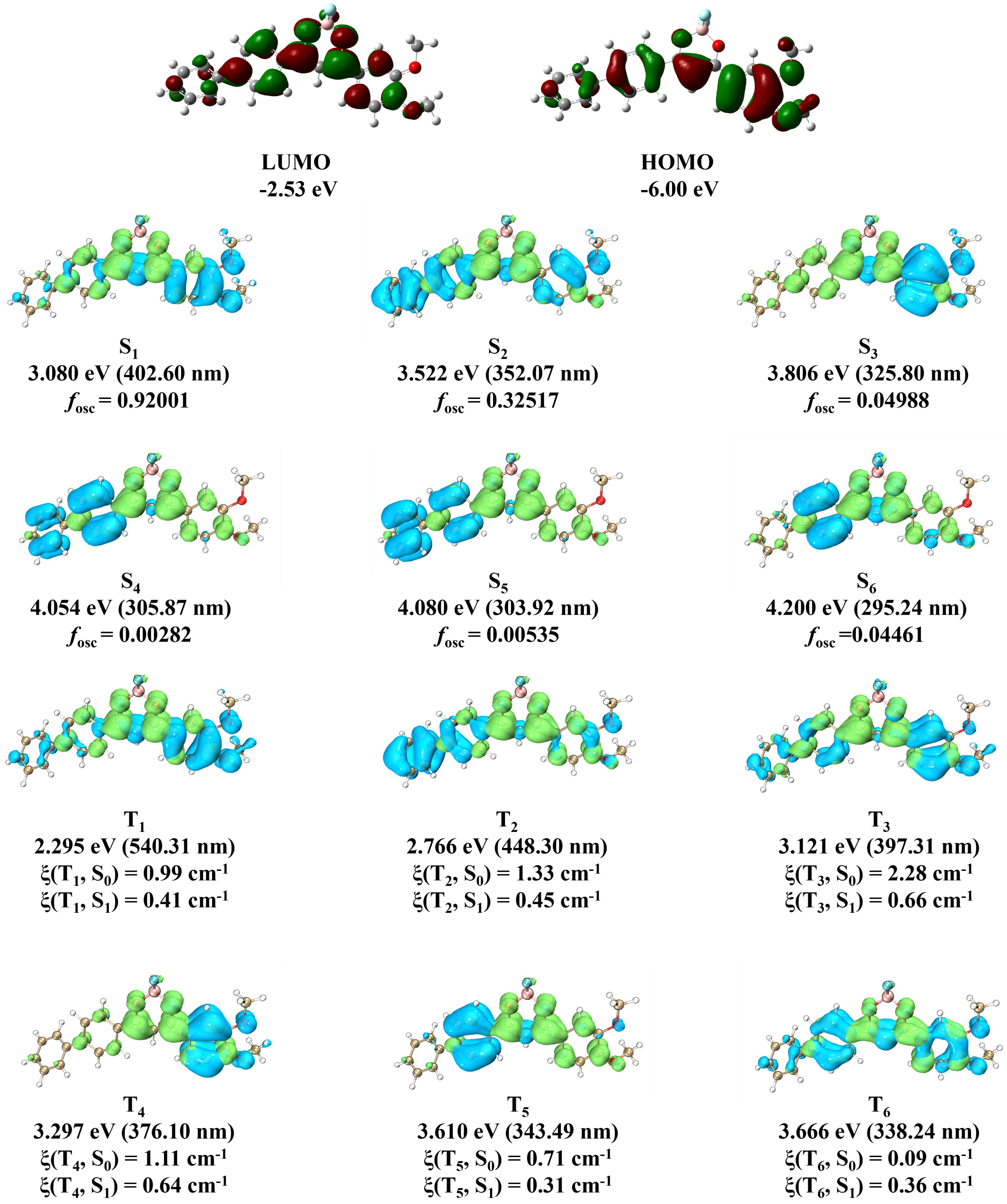

Figure S6. HOMO/LUMO energy levels of **3** calculated at B3LYP/6-31G(d,p) level. Iso-surface maps of electron-hole density difference of **3**'s excited states calculated at B3LYP/def2-TZVP(-f) level, where blue and green iso-surfaces correspond to hole and electron distributions, respectively, and excitation energies, oscillator strengths and spin–orbit coupling matrix element (SOCME) values.

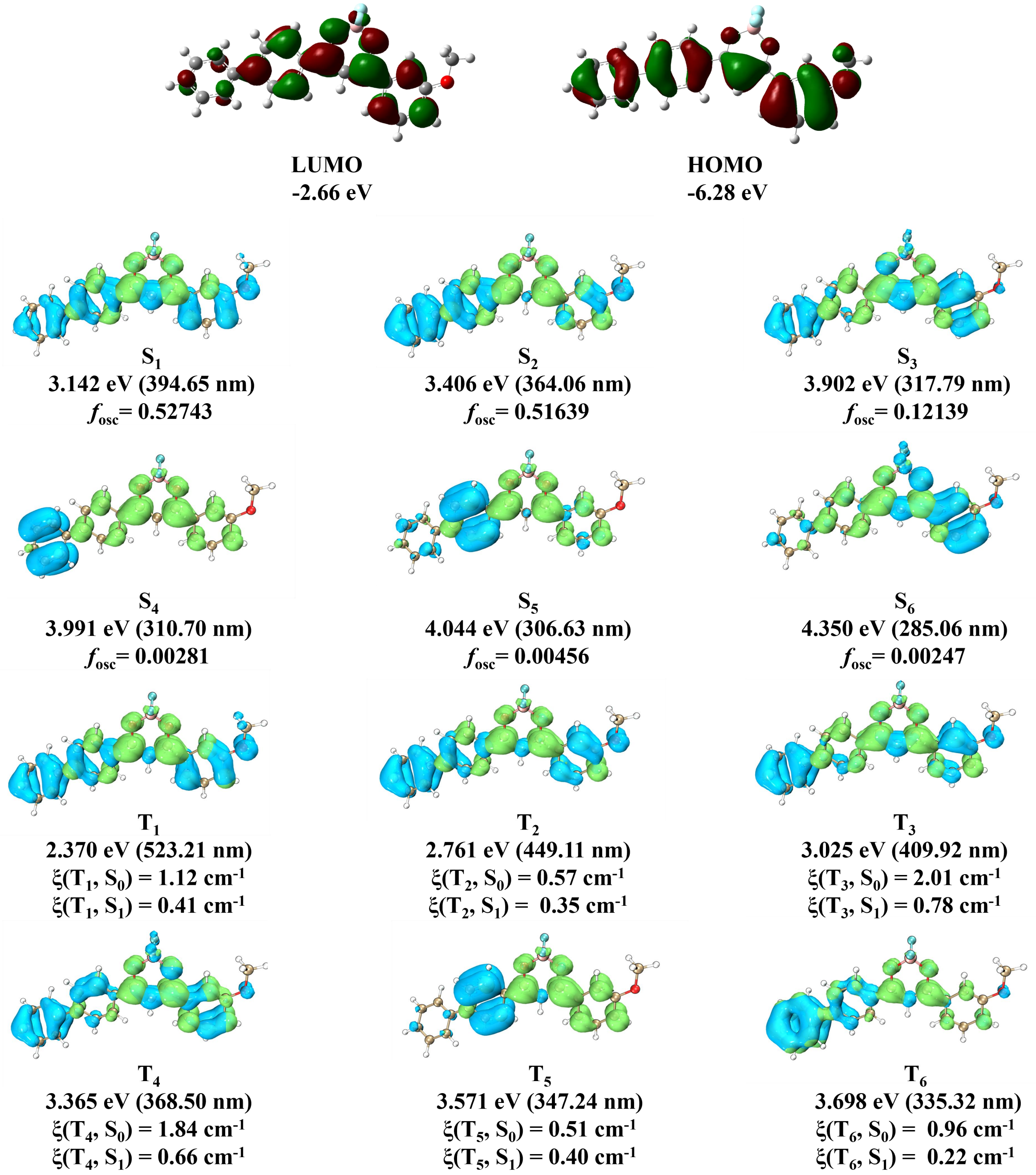

Figure S7. HOMO/LUMO energy levels of **4** calculated at B3LYP/6-31G(d,p) level. Iso-surface maps of electron-hole density difference of **4**'s excited states calculated at B3LYP/def2-TZVP(-f) level, where blue and green iso-surfaces correspond to hole and electron distributions, respectively, and excitation energies, oscillator strengths and spin–orbit coupling matrix element (SOCME) values.

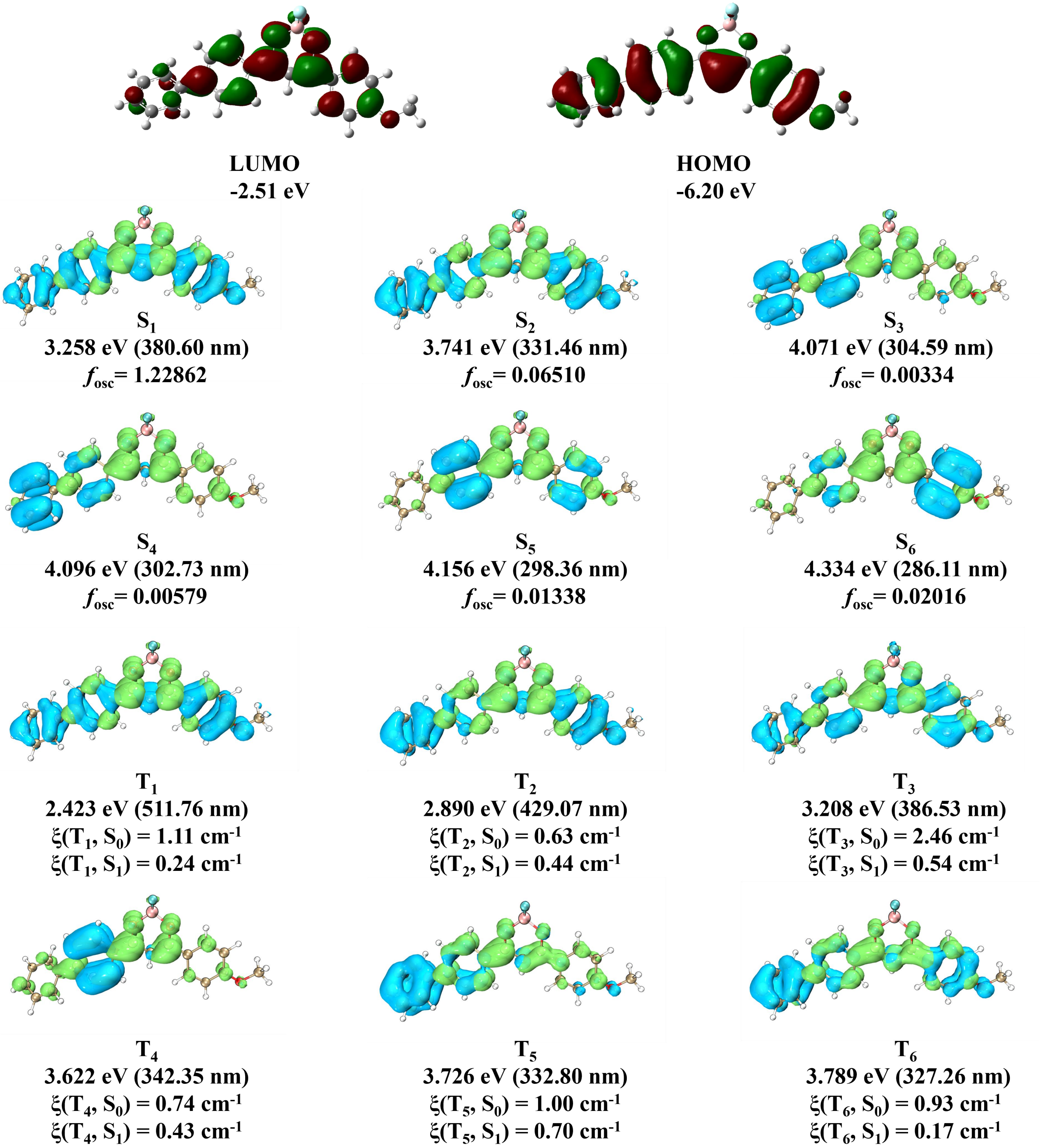

Figure S8. HOMO/LUMO energy levels of **5** calculated at B3LYP/6-31G(d,p) level. Iso-surface maps of electron-hole density difference of **5**'s excited states calculated at B3LYP/def2-TZVP(-f) level, where blue and green iso-surfaces correspond to hole and electron distributions, respectively, and excitation energies, oscillator strengths and spin–orbit coupling matrix element (SOCME) values.

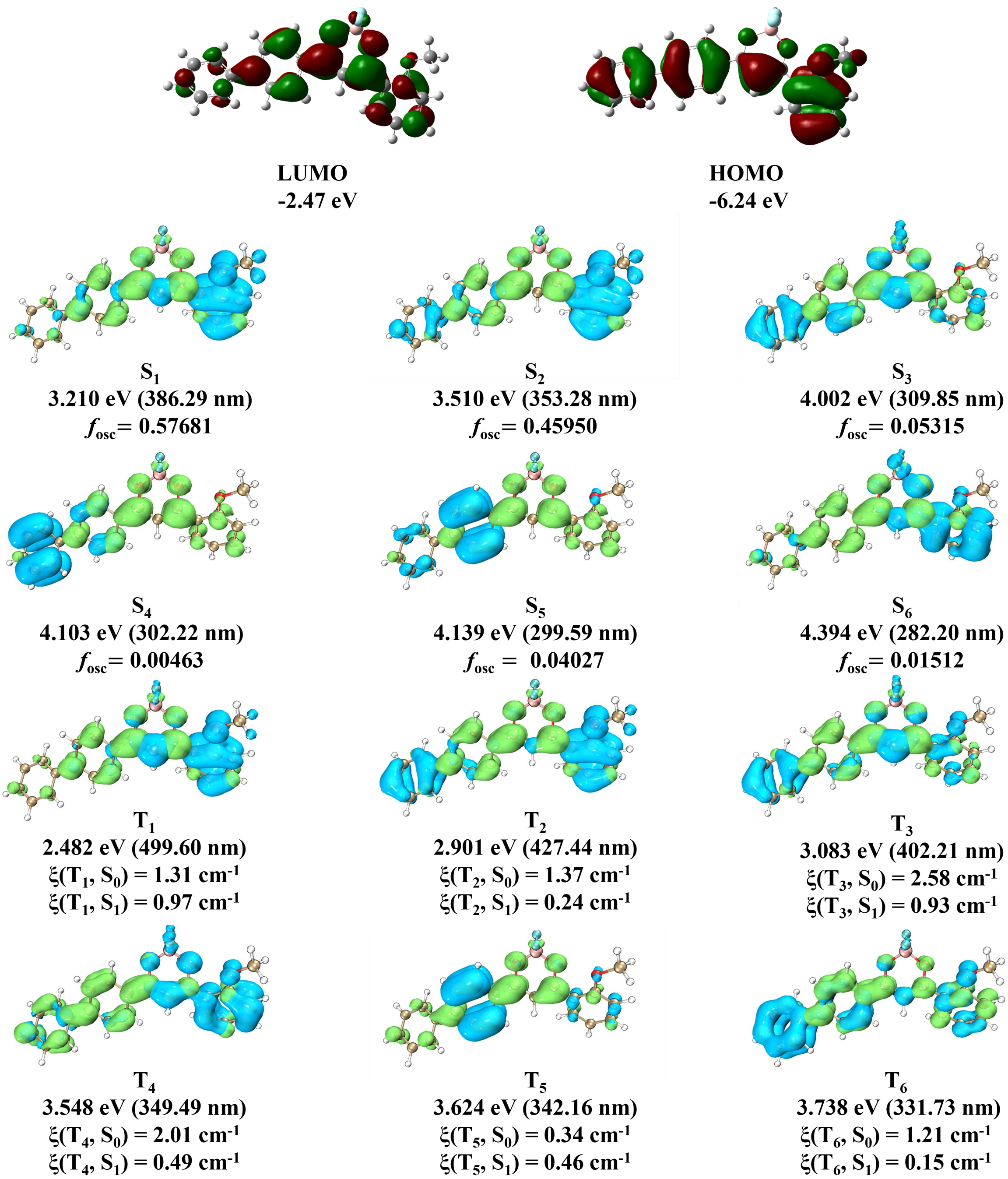

Figure S9. HOMO/LUMO energy levels of **6** calculated at B3LYP/6-31G(d,p) level. Iso-surface maps of electron-hole density difference of **6**'s excited states calculated at B3LYP/def2-TZVP(-f) level, where blue and green iso-surfaces correspond to hole and electron distributions, respectively, and excitation energies, oscillator strengths and spin–orbit coupling matrix element (SOCME) values.

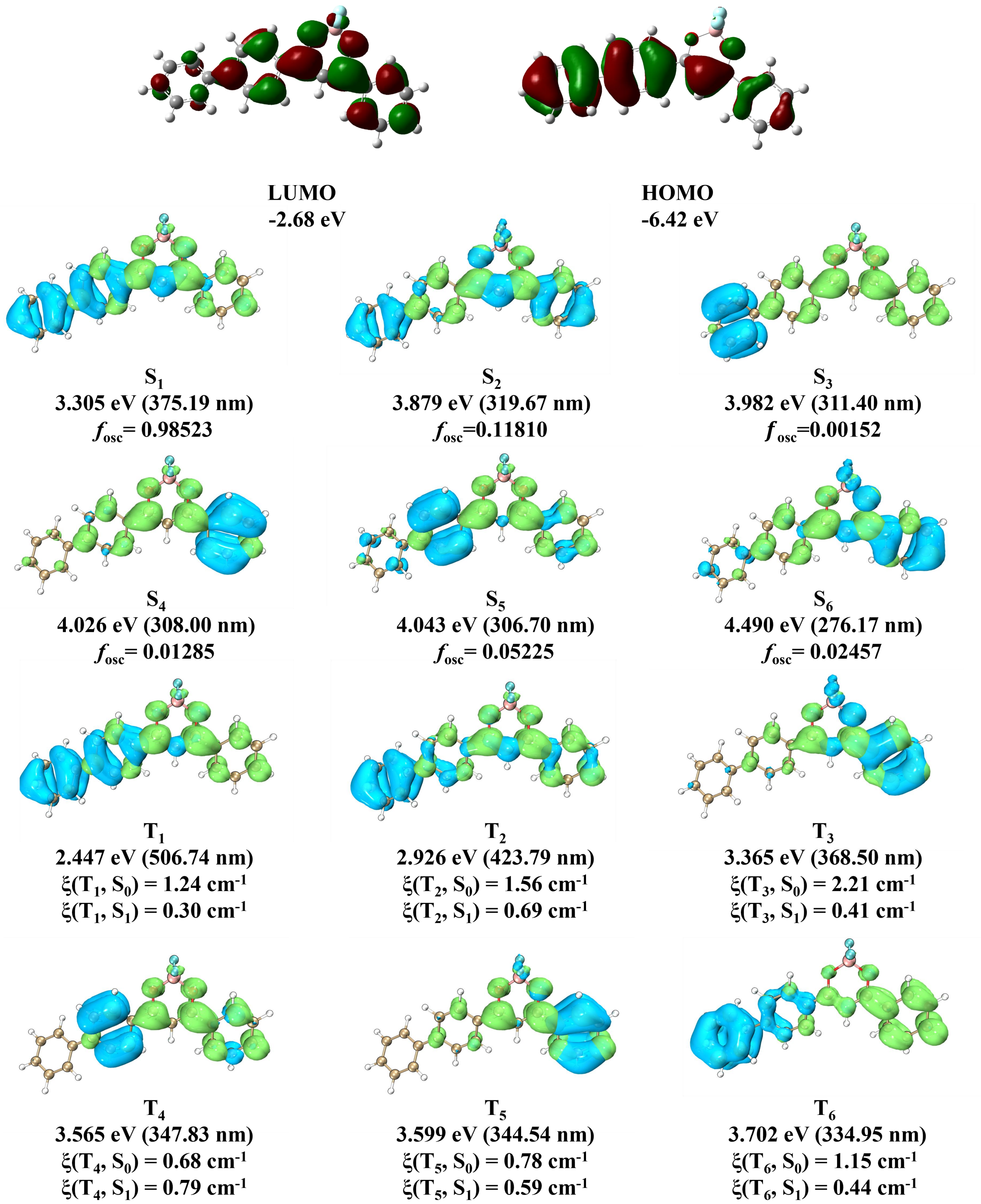

Figure S10. HOMO/LUMO energy levels of **7** calculated at B3LYP/6-31G(d,p) level. Iso-surface maps of electron-hole density difference of **7**'s excited states calculated at B3LYP/def2-TZVP(-f) level, where blue and green iso-surfaces correspond to hole and electron distributions, respectively, and excitation energies, oscillator strengths and spin–orbit coupling matrix element (SOCME) values.

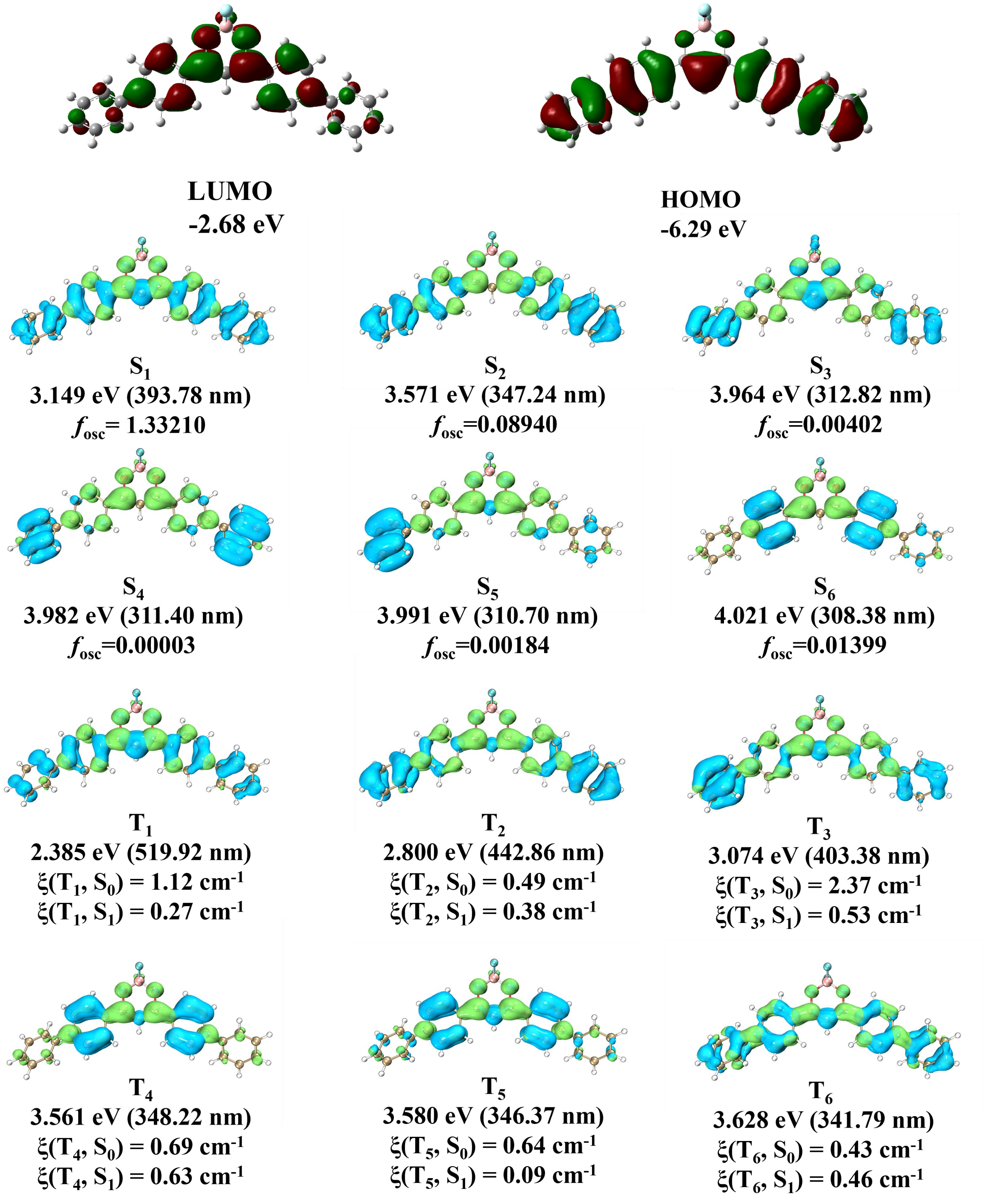

Figure S11. HOMO/LUMO energy levels of **8** calculated at B3LYP/6-31G(d,p) level. Iso-surface maps of electron-hole density difference of **8**'s excited states calculated at B3LYP/def2-TZVP(-f) level, where blue and green iso-surfaces correspond to hole and electron distributions, respectively, and excitation energies, oscillator strengths and spin–orbit coupling matrix element (SOCME) values.

Table S2. HOMO and LUMO of **1-8** calculated by DFT-B3LYP/6-31G(d, p).

Compounds	**1**	**2**	**3**	**4**	**5**	**6**	**7**	**8**
HOMO/ eV	-6.09	-6.11	-6.00	-6.28	-6.20	-6.24	-6.42	-6.29
LUMO/ eV	-2.51	-2.58	-2.53	-2.66	-2.51	-2.47	-2.68	-2.68

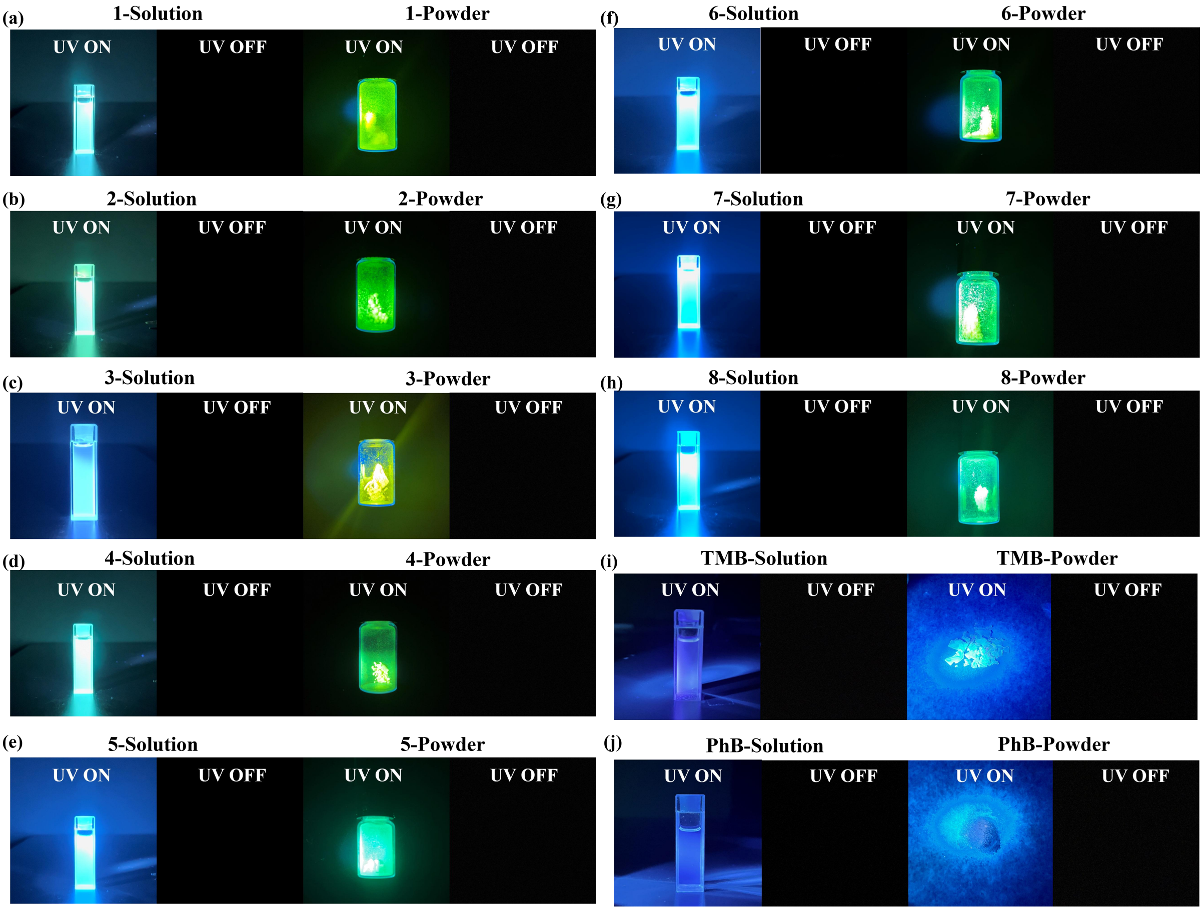

Figure S12. Photographs of compounds **1-8**, TMB and PhB in solid state and in dichloromethane solution under 365 nm UV light and after the removal of the UV light.

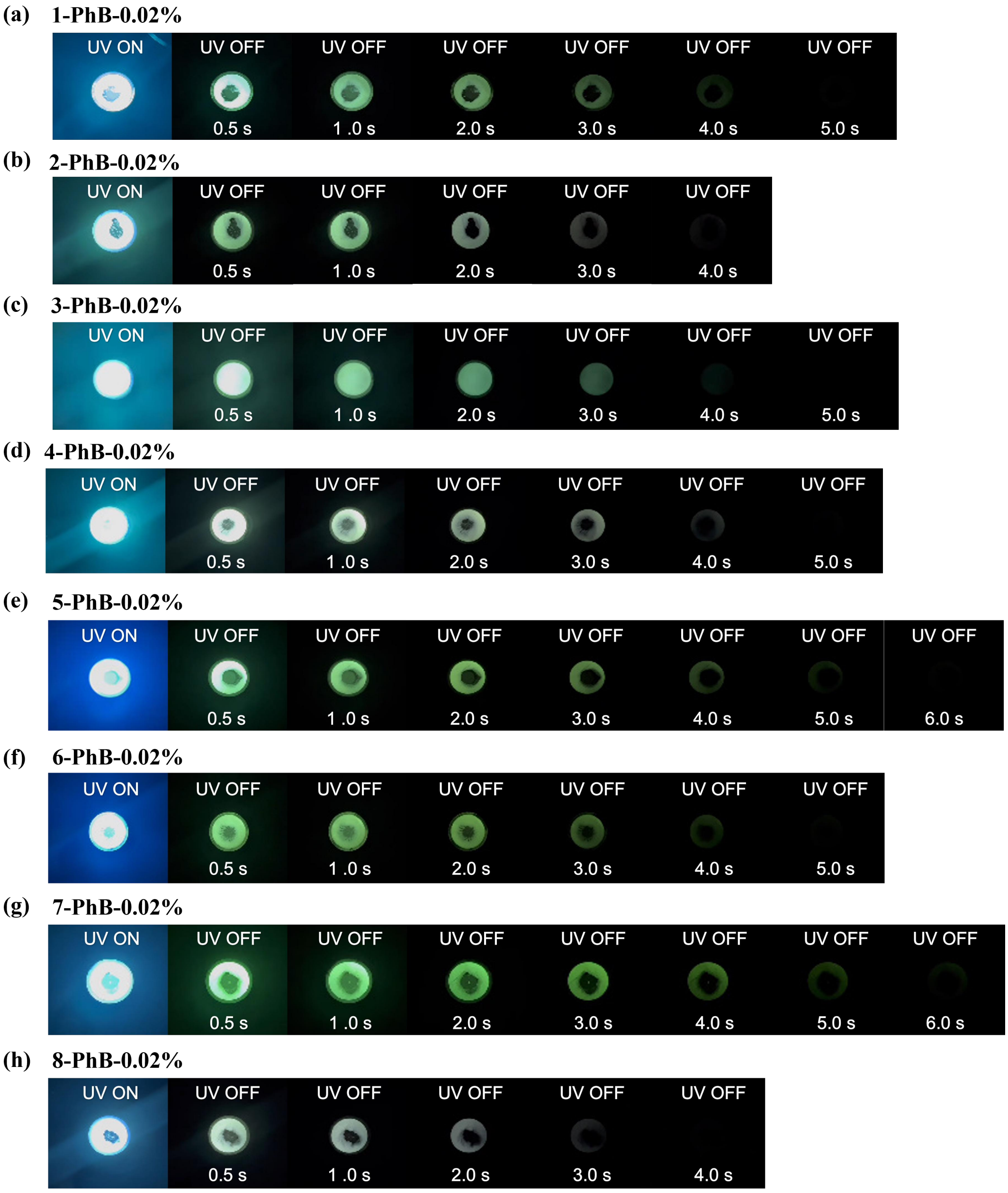

Figure S13. Photographs of **BF_2bdk**-PhB-0.02% afterglow materials under a 365 nm UV light and after the removal of the UV light.

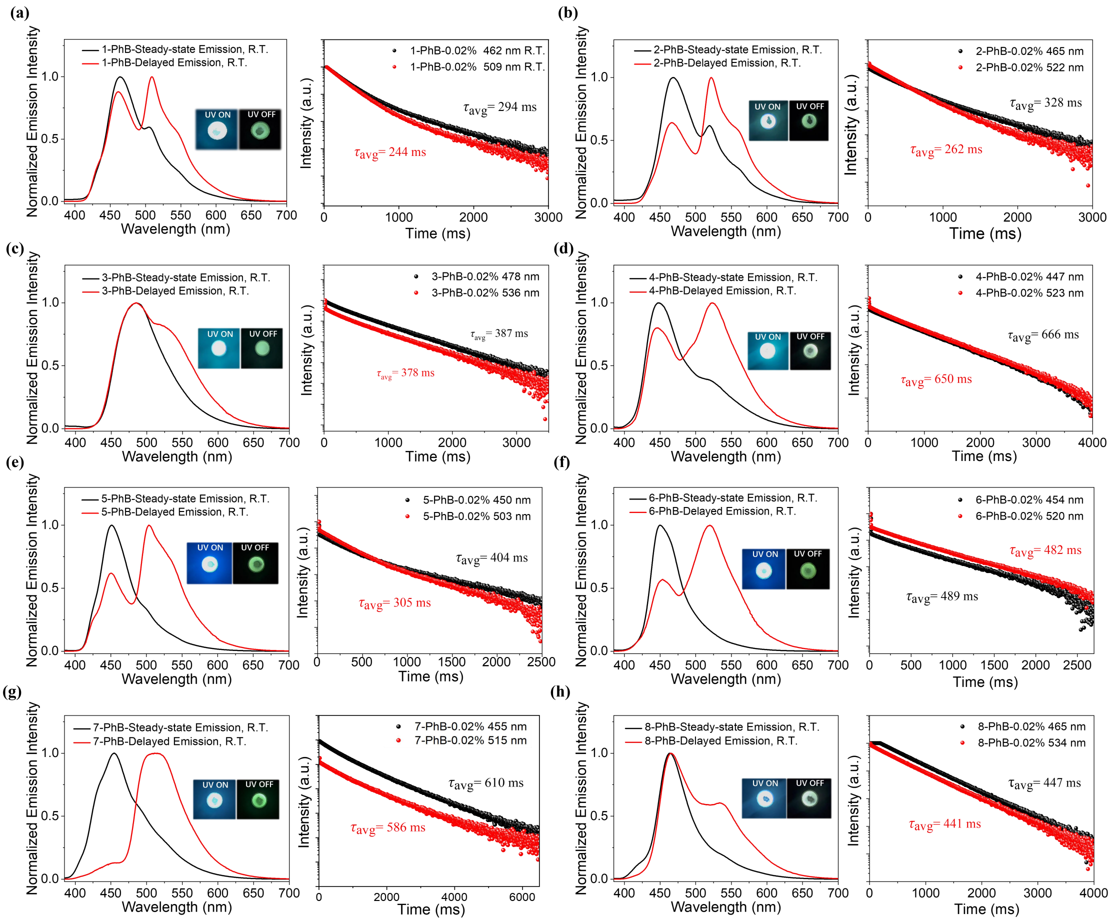

Figure S14. (a-h) Steady-state and delayed emission (1 ms delay) spectra, emission decay curve monitored at different emission wavelength of **BF$_2$bdk**-PhB-0.02% samples excited at 365 nm.

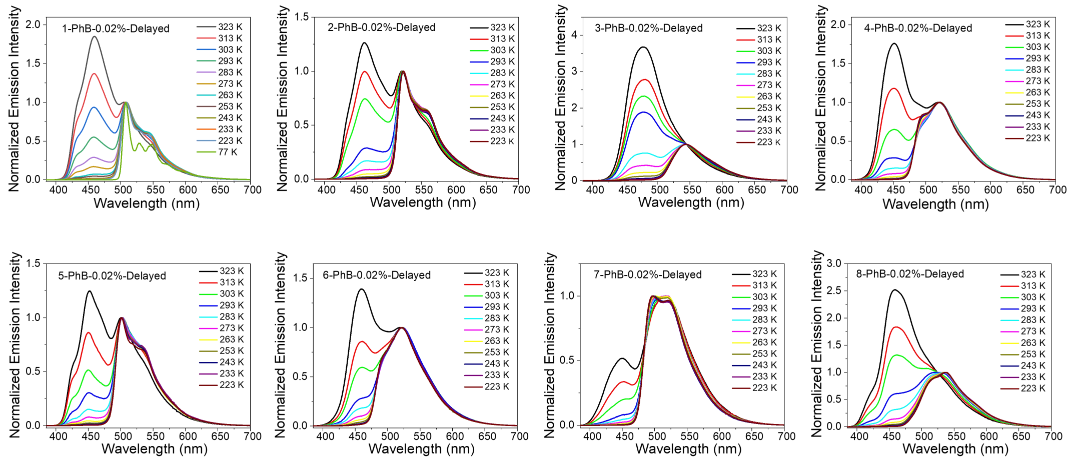

Figure S15. Temperature-dependent delayed emission spectra of **BF$_2$bdk**-PhB-0.02% samples excited at 365 nm.

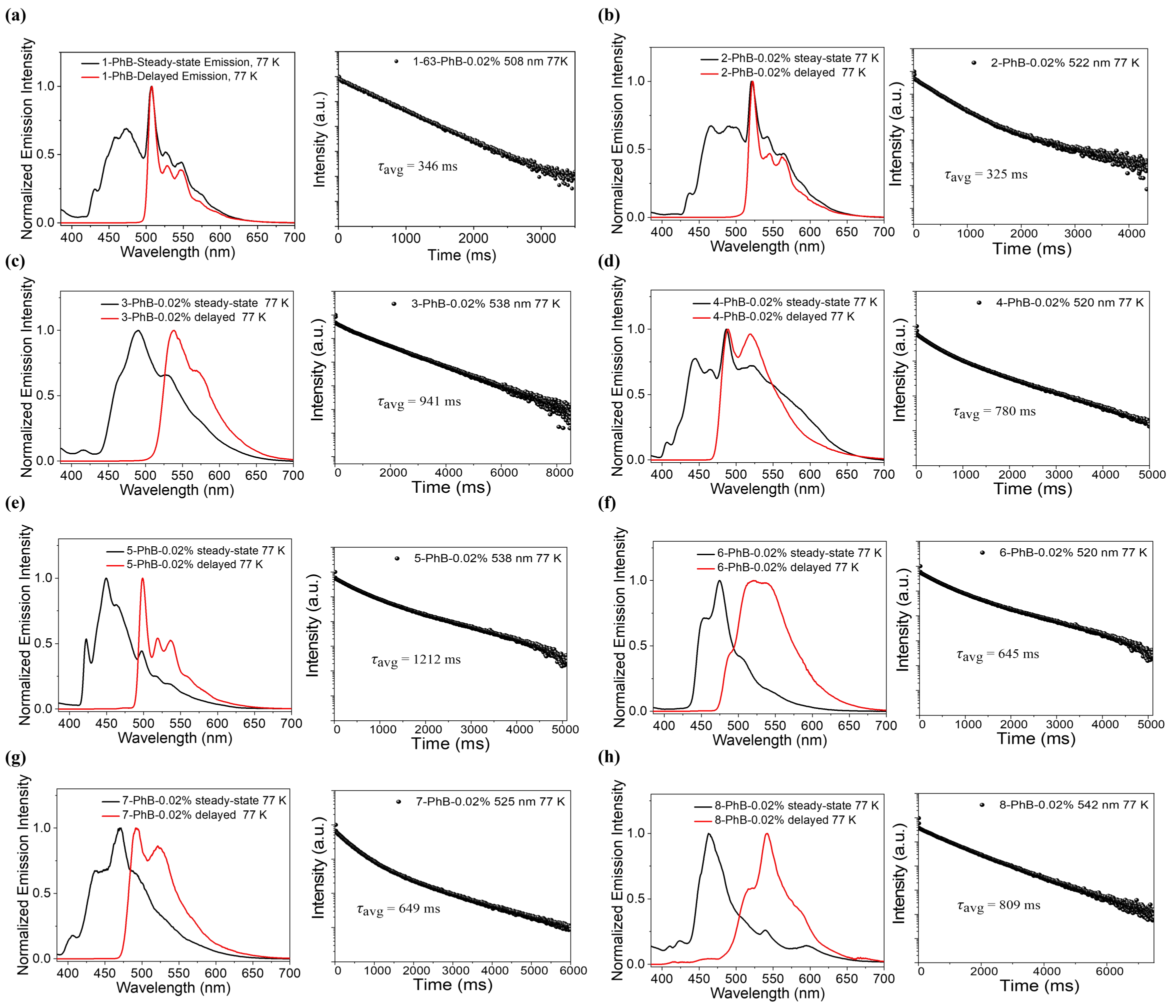

Figure S16. (a-g) Steady-state and delayed emission (1 ms delay) spectra, emission decay curve of **BF_2bdk**-PhB-0.02% samples at 77 K excited at 365 nm.

Table S3. Photophysical results of **BF_2bdk**-PhB-0.02% samples

Entry	$S_{1,\,exp}$[a] / eV	$T_{1,\,exp}$[b] / eV	$T_{1,\,cal}$[c] / eV	$T_{2,\,cal}$[c] / eV	$T_{2,cal}$-$S_{1,\,exp}$ / eV	$\xi(S_1$-$T_1)$[c] / cm^{-1}	$\xi(S_1$-$T_2)$[c] / cm^{-1}	$\xi(S_1$-$T_3)$[c] / cm^{-1}
1	2.71	2.44	2.368	2.817	0.11	0.24	0.36	0.63
2	2.61	2.38	2.348	2.701	0.09	0.30	0.49	0.58
3	2.60	2.31	2.295	2.766	0.17	0.41	0.45	0.66
4	2.77	2.39	2.37	2.761	-0.01	0.41	0.35	0.78
5	2.75	2.48	2.423	2.890	0.14	0.24	0.44	0.54
6	2.76	2.38	2.482	2.901	0.14	0.97	0.24	0.93
7	2.73	2.52	2.447	2.926	0.20	0.30	0.69	0.41
8	2.67	2.39	2.385	2.800	0.13	0.27	0.38	0.53

[a] Estimated from fluorescence maxima of **BF_2bdk**-PhB-0.02% samples. [b] Estimated from phosphorescence maxima of **BF_2bdk**-PhB-0.02% samples at 77 K. [c] Calculated by TD-B3LYP/def2-TZVP(-f) based on optimized S_0 geometry.

1-TMB-PhB-0.2% Bulk solid **1-TMB-PhB-0.2% Film**

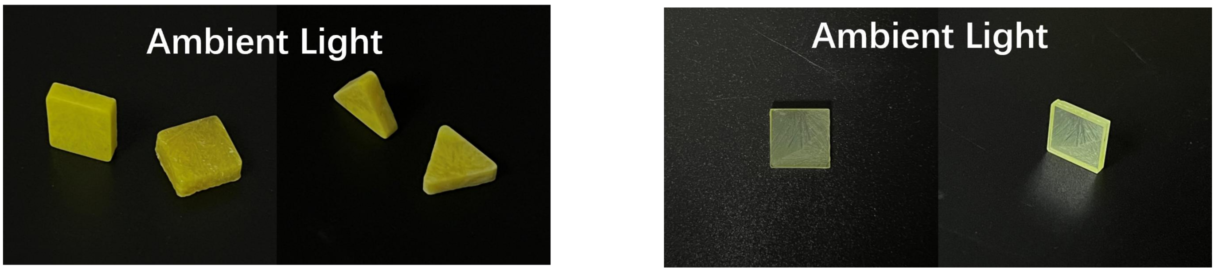

Figure S17. Shape of 1-TMB-PhB-0.2% bulk solid (left) and 1-TMB-PhB-0.2% film (10 mm × 10 mm, thickness: 20 μm, right) sandwiched between two quartz plates.

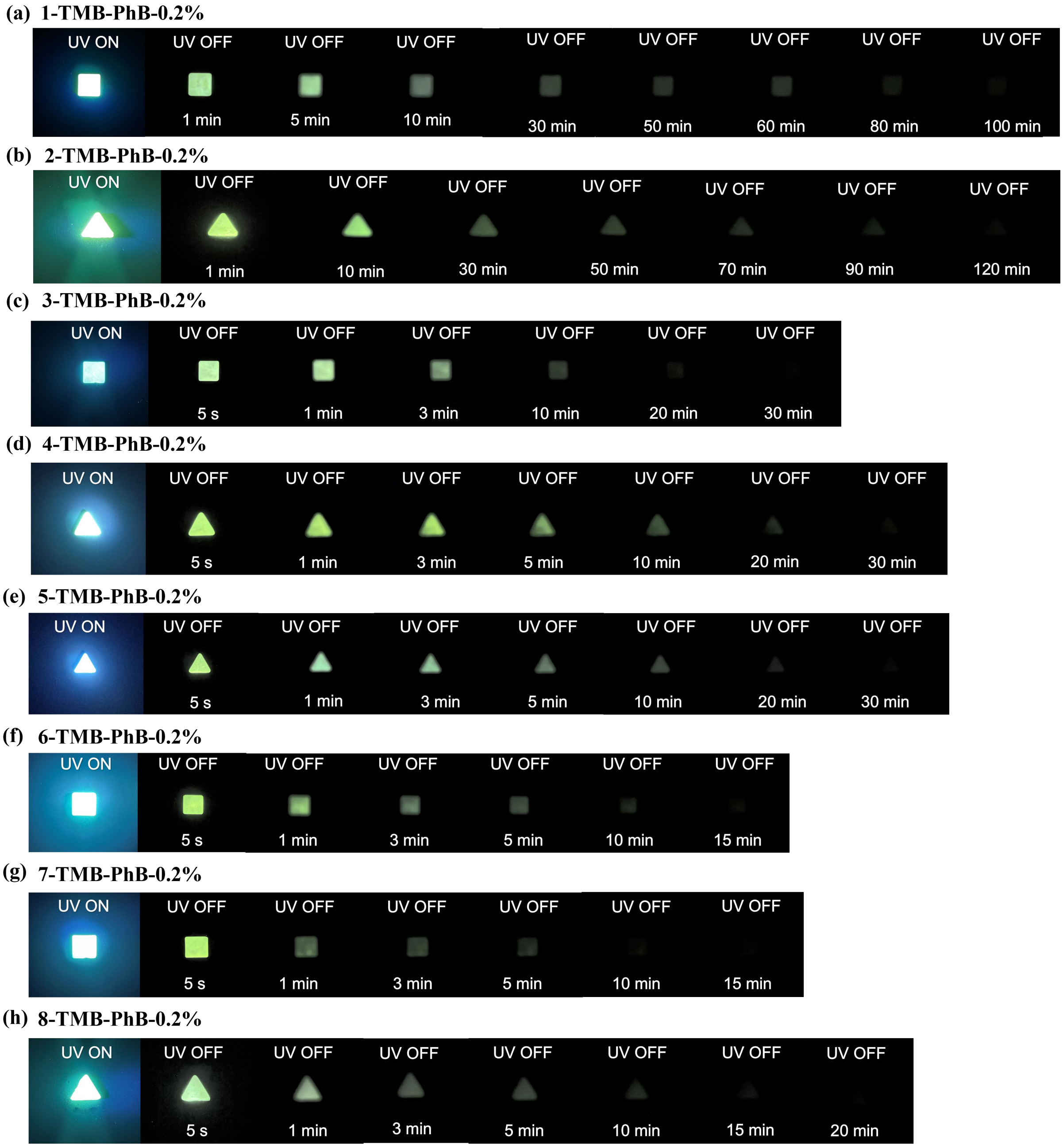

Figure S18. Photographs of **BF_2bdk**-TMB-PhB-0.2% afterglow materials under a 365 nm UV light and after the removal of the UV light.

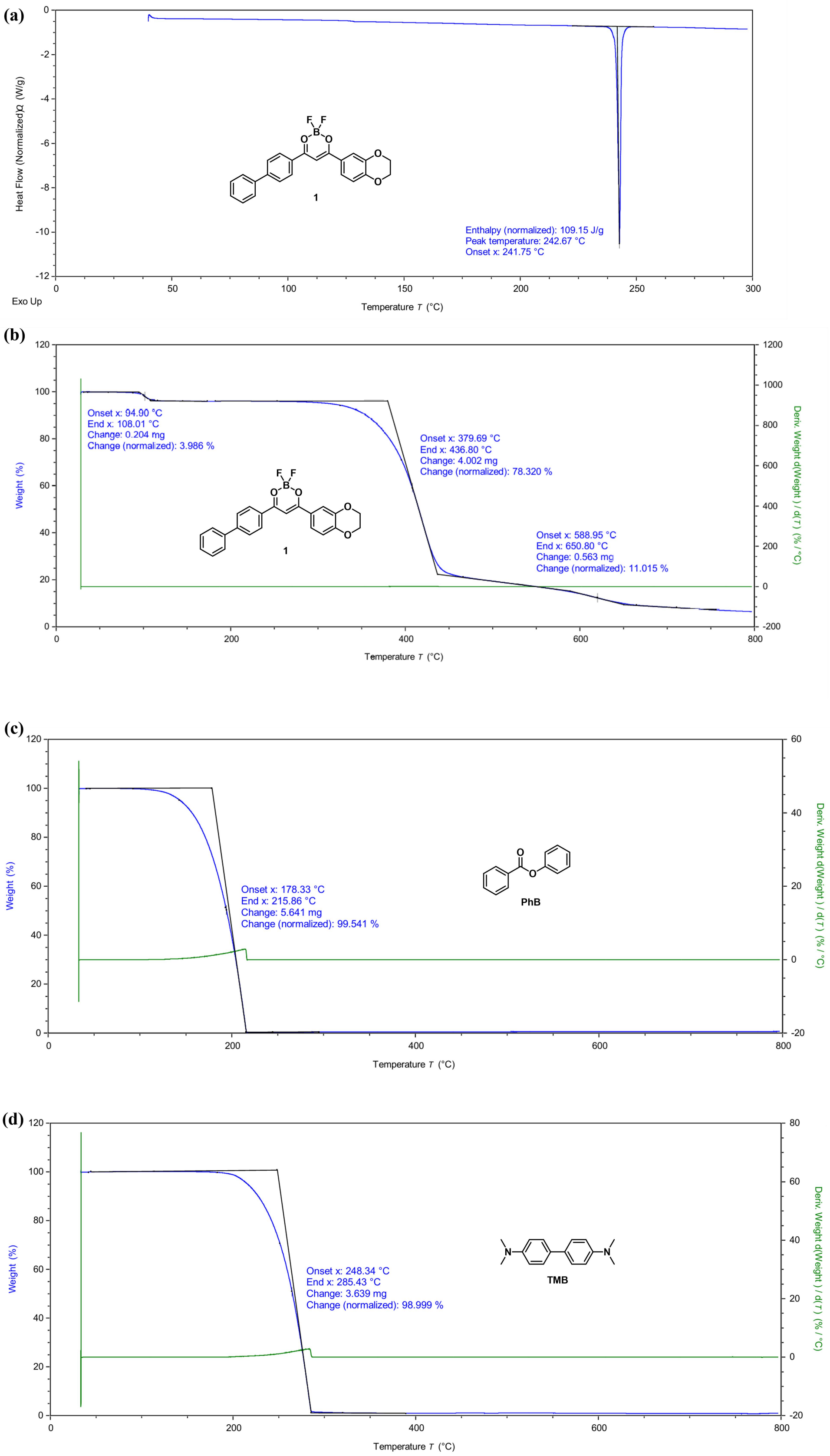

Figure S19. (a) DSC curve of compound 1. (b)TGA curve of 1. (c) TGA curve of TMB (d)TGA curve of PhB.

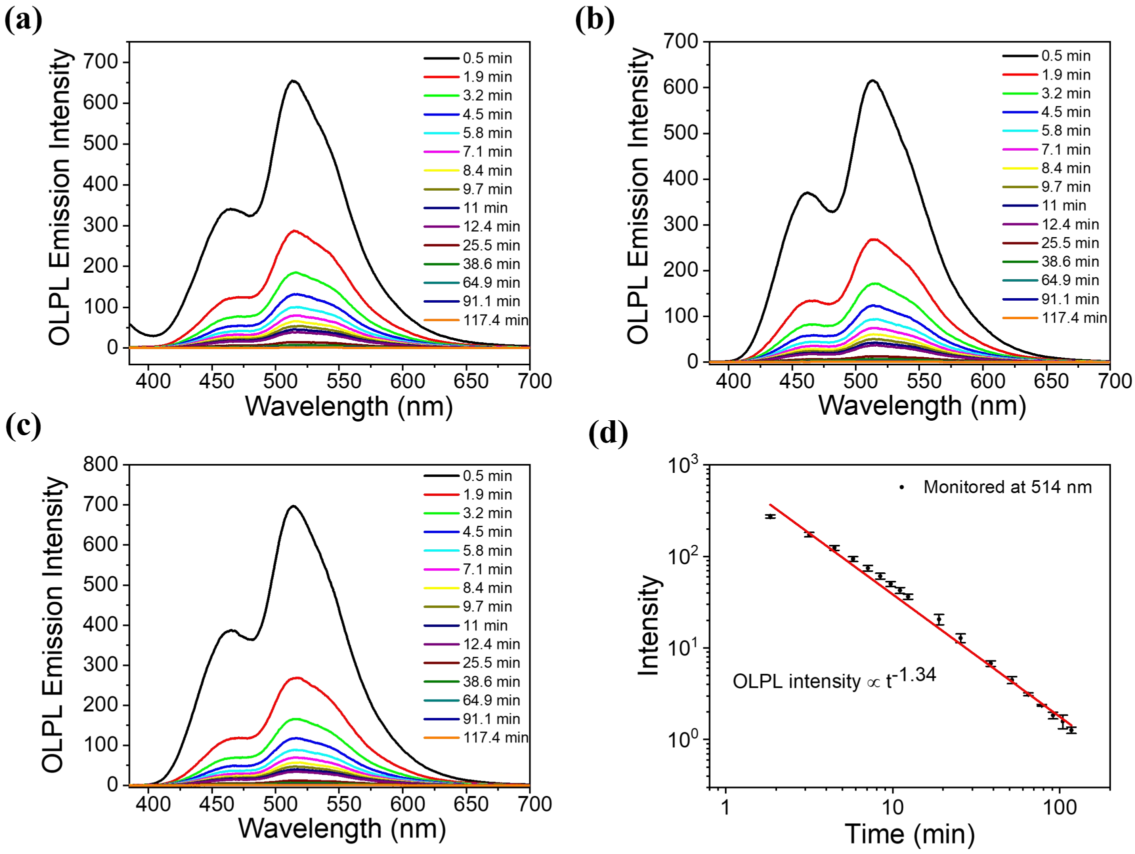

Figure S20. (a-c) Delayed emission spectra of three parallel prepared **1**-TMB-PhB-0.2% materials excited at 365 nm as a function of delay time. (d) Average logarithmic plot of the OLPL emission intensity (monitored at 514 nm) of three parallel prepared **1**-TMB-PhB-0.2% materials at different delayed times (error bars included).

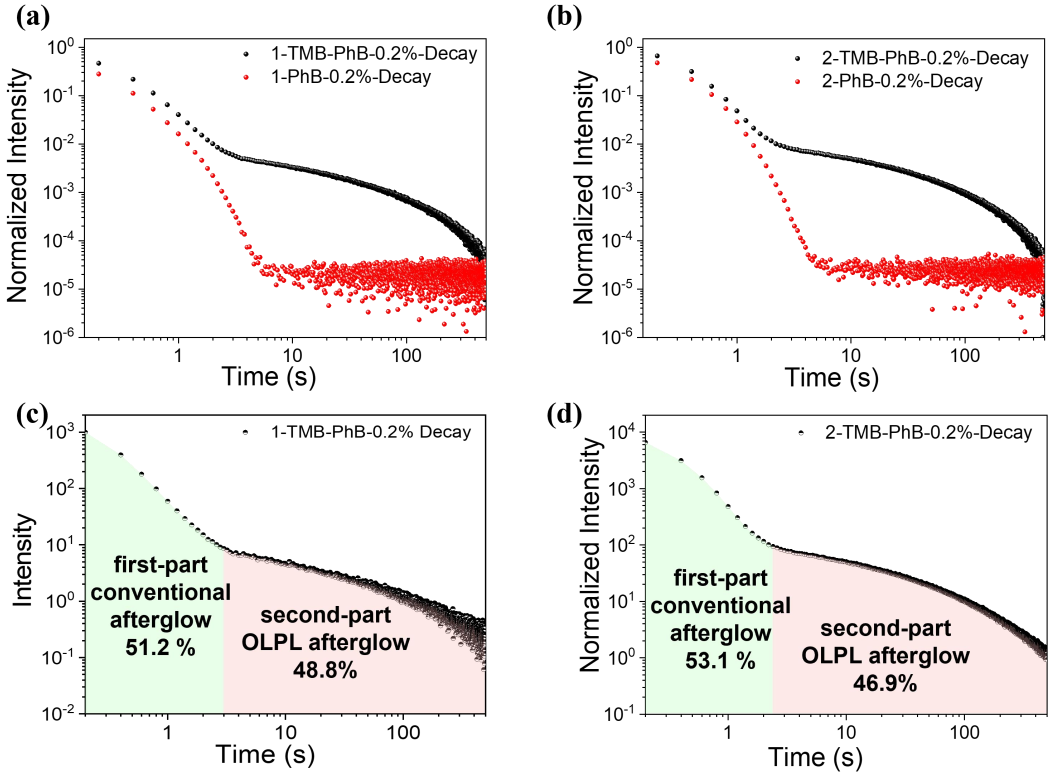

Figure S21. (a) Room-temperature emission decay profiles of **1**-PhB-0.2% and **1**-TMB-PhB-0.2% materials monitored at 514 nm and excited at 365 nm. (b) Room-temperature emission decay profiles of **2**-PhB-0.2% and **2**-TMB-PhB-0.2% materials monitored at 525 nm and excited at 365 nm. (c) Room-temperature afterglow decay profiles (1 ms delay, monitored at 514 nm, excited at 365 nm) of **1**-TMB-PhB-0.2% materials. (d) Room-temperature afterglow decay profiles (1 ms delay, monitored at 525 nm, excited at 365 nm) of **2**-TMB-PhB-0.2% materials.

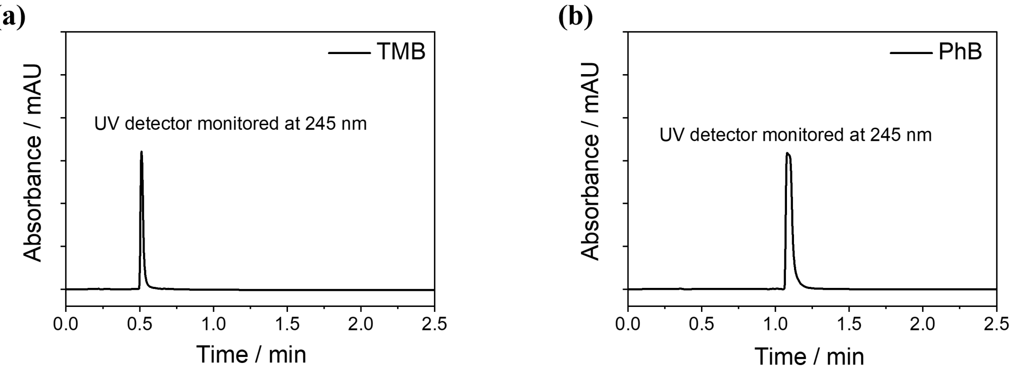

Figure S22. HPLC profiles of (a) PhB and (b) TMB monitored at 245 nm.

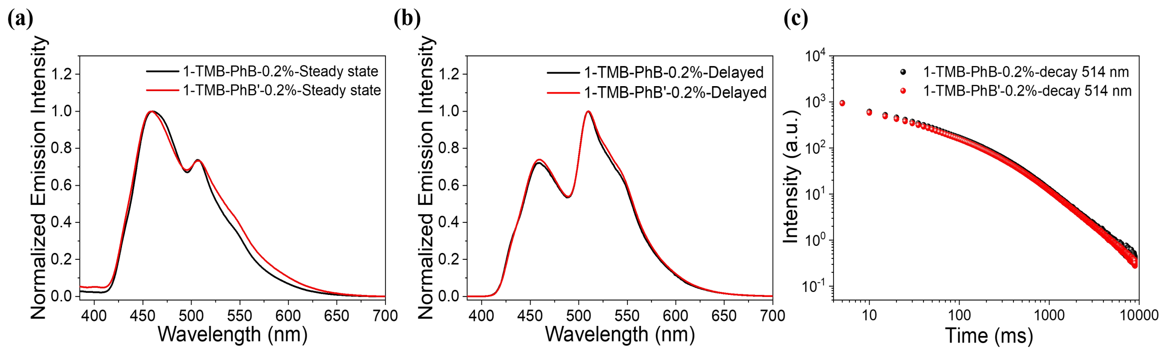

Figure S23. (a) Room-temperature steady-state and (b) delayed emission spectra (1 ms delay) and (c) emission decay profile of **1**-TMB-PhB-0.2% and **1**-TMB-PhB' samples (PhB' refers to the PhB after recrystallization).

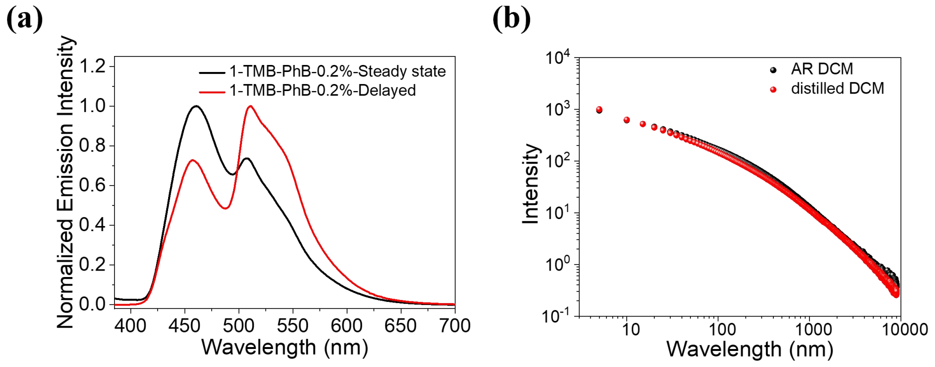

Figure S24. (a) Room-temperature steady-state and delayed emission spectra (1 ms delay) of **1**-TMB-PhB-0.2% sample (prepared using distilled DCM). (b) Emission decay profile of **1**-TMB-PhB-0.2% samples (prepared using commercial AR grade DCM and distilled DCM).

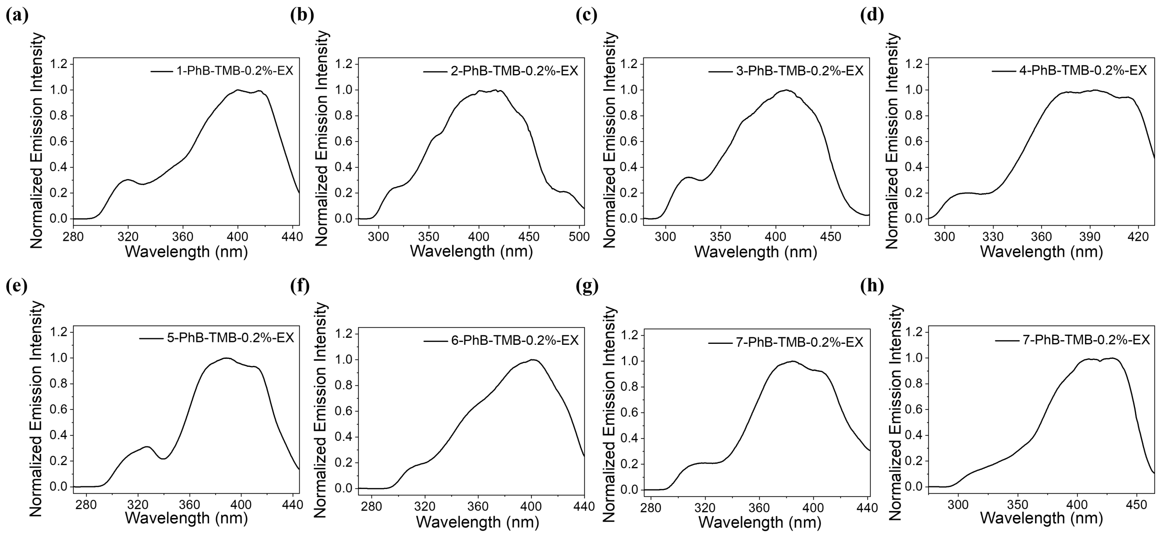

Figure S25. Excitation spectra of **BF$_2$bdk**-TMB-PhB-0.2% samples.

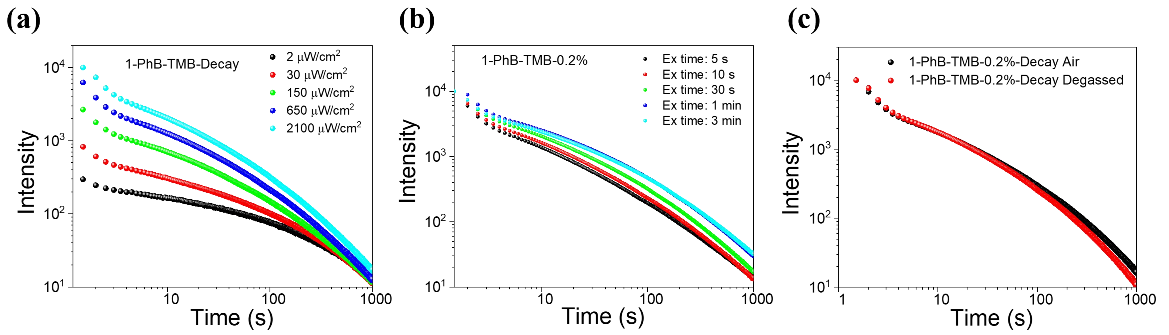

Figure S26. (a) Room-temperature emission decay profile (mointoired at 514 nm) of **1**-TMB-PhB-0.2% excited by 365 nm of different excitation powers for 1 min. (b) Room-temperature emission decay profile (mointoired at 514 nm) of **1**-TMB-PhB-0.2% excited by 365 nm of equipment for different excitation time (excitation power: 2.1 mW/cm^2). (c) Room-temperature emission decay profile (mointoired at 514 nm) of **1**-TMB-PhB-0.2% excited by 365 nm under air and degassed condition.

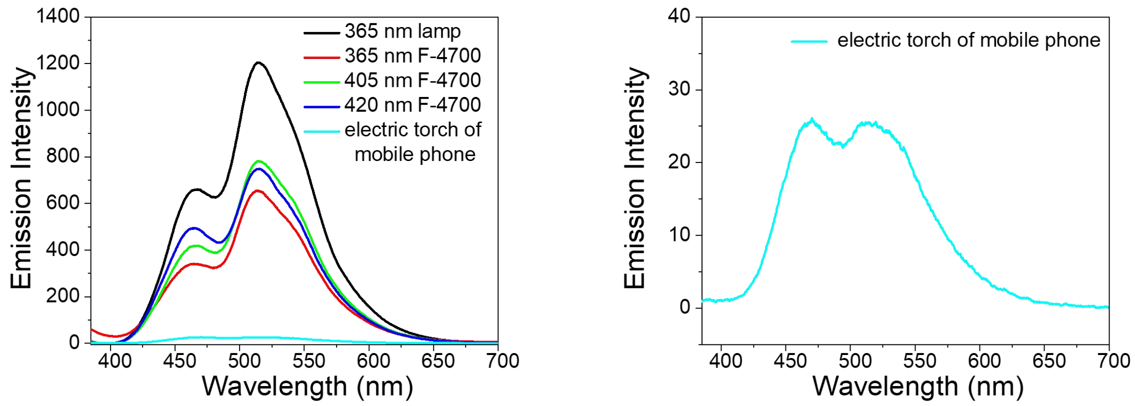

Figure S27. Room-temperature OLPL emission spectra (left) of **1**-TMB-PhB-0.2% samples at delay time of around 0.5 min excited at 365 nm lamp (a handheld UV lamp, 5W), electric torch of mobile phone, 365 nm, 405 nm and 420 nm (excitation wavelengths of F-4700, EX/EM slits at 20/20 nm).

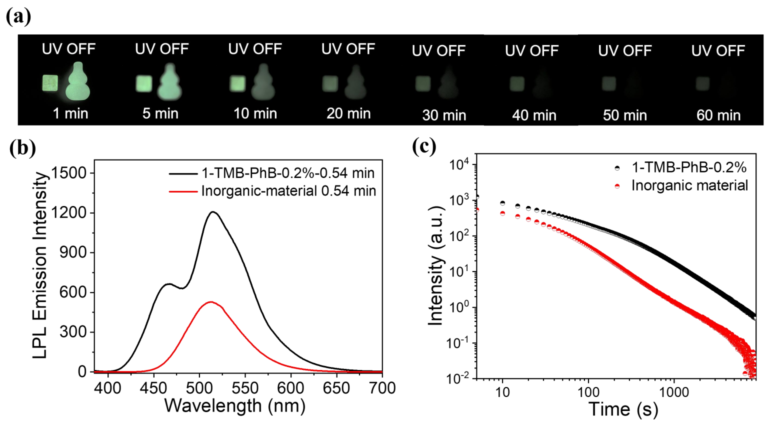

Figure S28. (a) Photographs of (left) **1**-TMB-PhB materials after removal of 365 nm UV lamp and (right) $Sr_2Al_{14}O_{25}/Eu^{2+}$, Dy^{3+} materials after ceasing room light. (b) Room-temperature LPL emission spectra of **1**-TMB-PhB materials and inorganic $Sr_2Al_{14}O_{25}/Eu^{2+}$, Dy^{3+} materials at delay time of 0.54 min. (c) Room-temperature emission decay profile of **1**-TMB-PhB materials (monitored at 514 nm) excited by 365 nm UV lamp and inorganic $Sr_2Al_{14}O_{25}/Eu^{2+}$, Dy^{3+} materials (monitored at 518 nm) excited by room light.

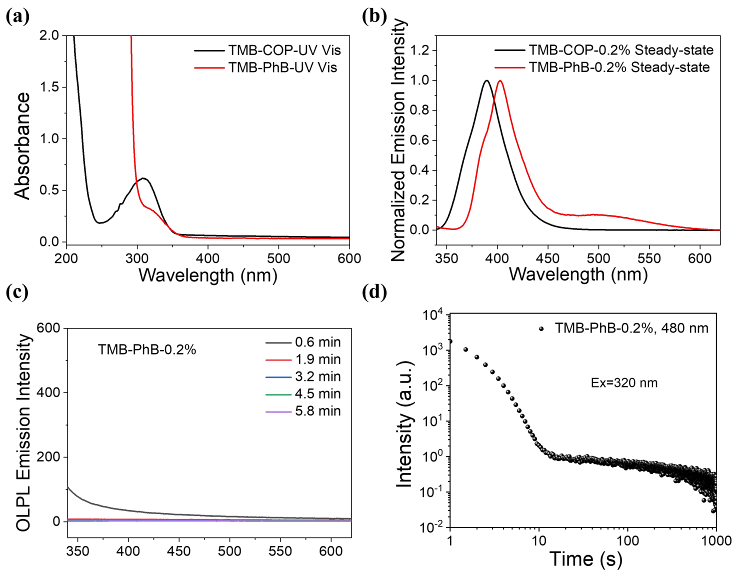

Figure S29. (a) UV-vis absorption spectra and (b) room-temperature steady-state emission spectra of TMB-COP-0.2% and TMB-PhB-0.2% samples excited at 320 nm. (c) Room-temperature delayed emission spectra of TMB-PhB-0.2% samples at different delay times. (d) Room-temperature emission decay profiles of TMB-PhB-0.2% samples excited at 320 nm.

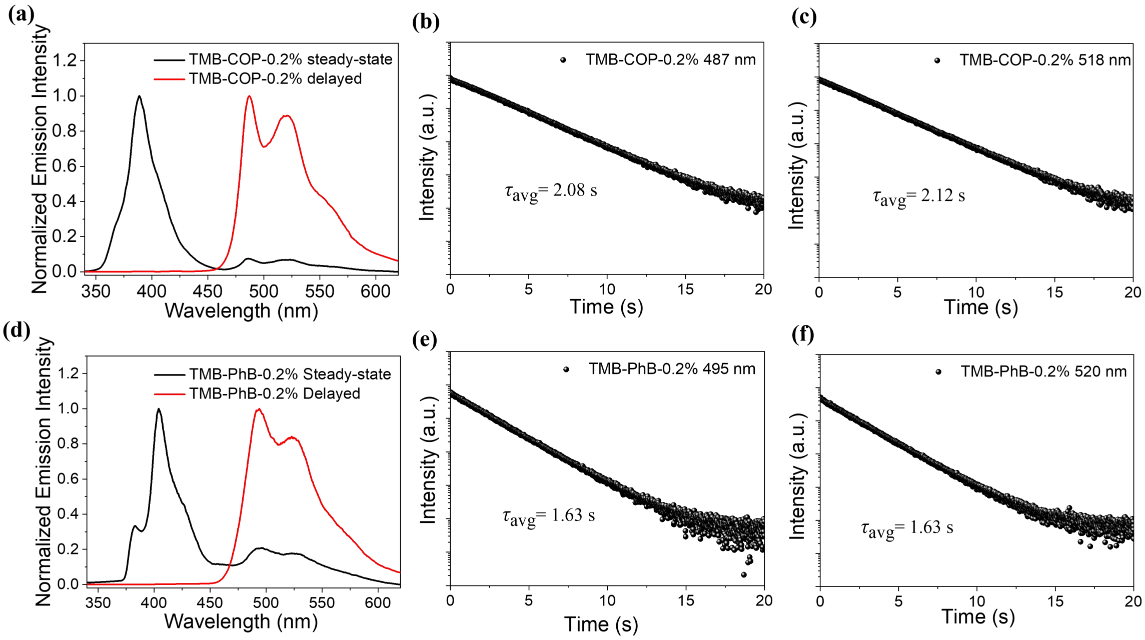

Figure S30. (a) Steady-state and delayed emission spectra (1 ms delay) and (b, c) emission decay profile of TMB-PhB-0.2% at 77 K excited at 320 nm. (d) Steady-state and delayed emission spectra (1 ms delay) and (e, f) emission decay profile of TMB-COP-0.2% at 77 K excited at 320 nm.

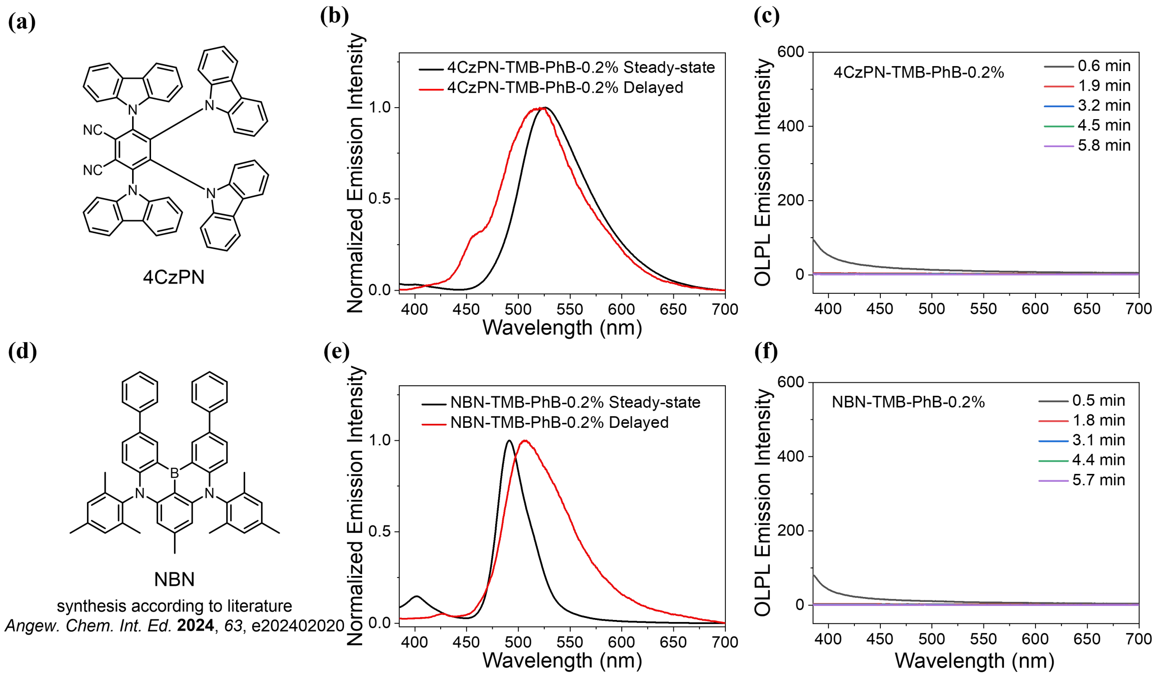

Figure S31. (a) Chemical structures of 4CzPN TADF emitter. (b) Steady-state and delayed emission spectra (1 ms delay) of 4CzPN-TMB-PhB-0.2% samples excited at 365 nm. (c) Delayed emission spectra (excited at 365 nm) of 4CzPN-TMB-PhB-0.2% samples recorded at different delay times. (d) Chemical structures of NBN TADF emitter. (e) Steady-state and delayed emission spectra (1 ms delay) of NBN-TMB-PhB-0.2% samples excited at 365 nm. (f) Delayed emission spectra (excited at 365 nm) of NBN-TMB-PhB-0.2% samples recorded at different delay times. It can be found that 4CzPN-TMB-PhB-0.2% and NBN-TMB-PhB-0.2% samples does not produce any OLPL afterglow.

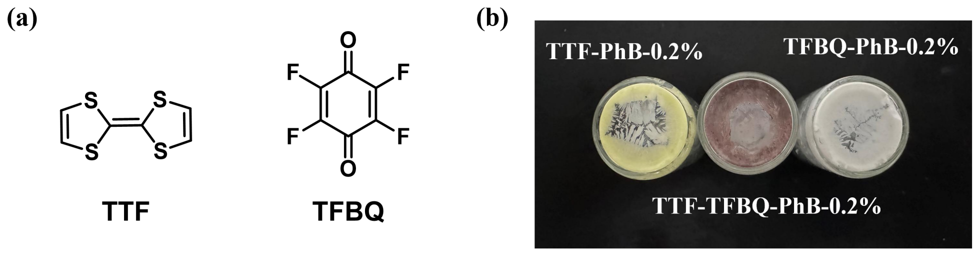

Figure S32. (a) Molecular structure of TTF and TFBQ. (b) Photographs of melt-cast samples of **TTF**-PhB-0.2%, **TFBQ**-PhB-0.2% and **TTF-TFBQ**-PhB-0.2% under room light.

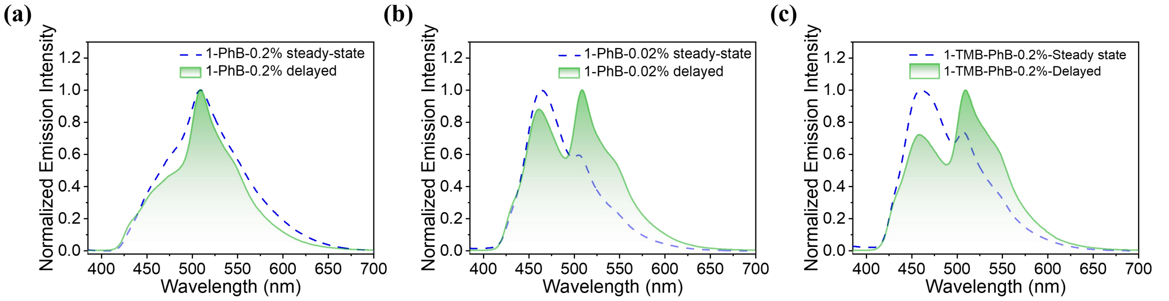

Figure S33. Room-temperature steady-state and delayed emission spectra (1 ms delay) of (a) **1**-PhB-0.2%, (b) **1**-PhB-0.02% and (c) **1**-TMB-PhB-0.2% samples.

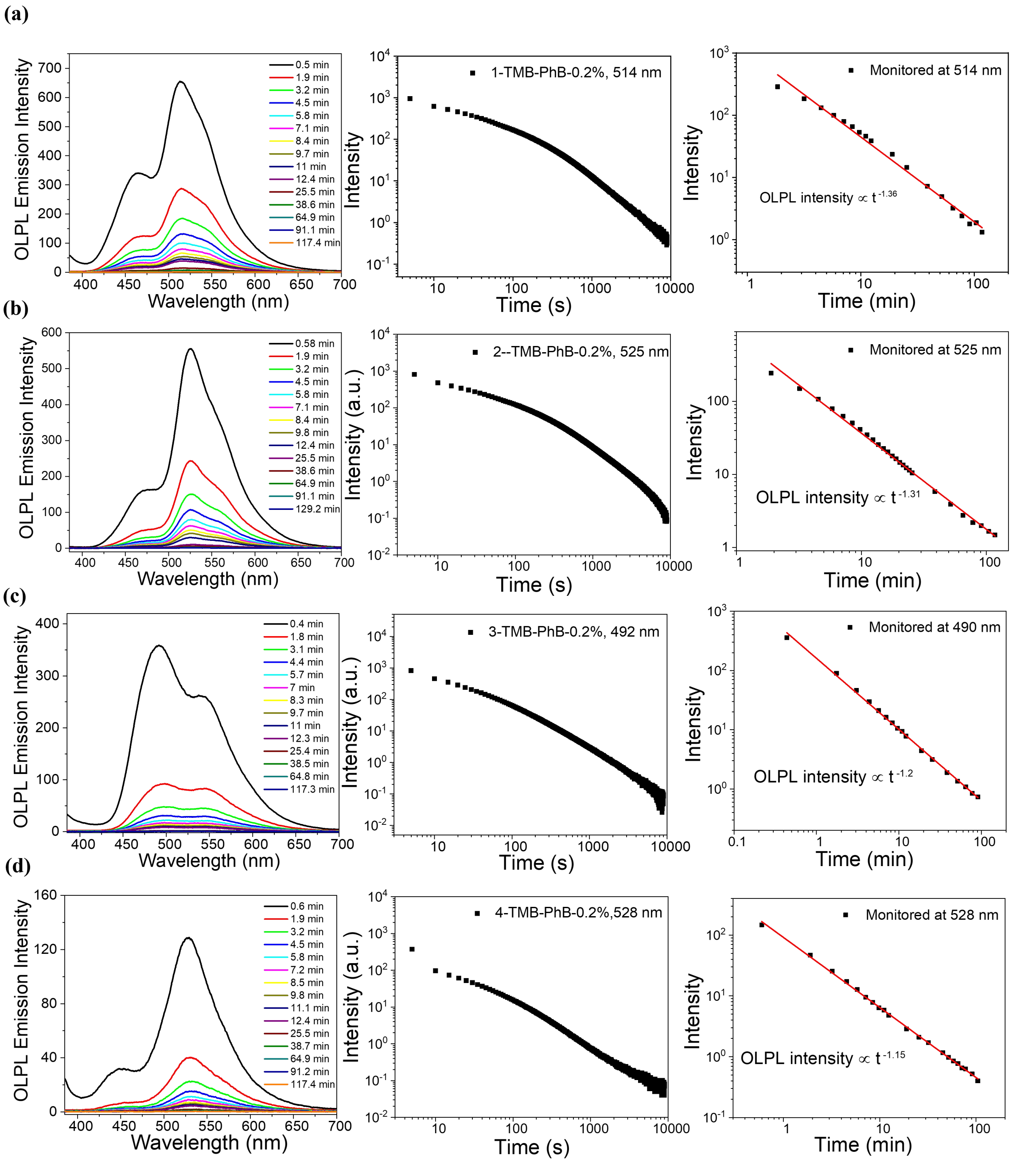

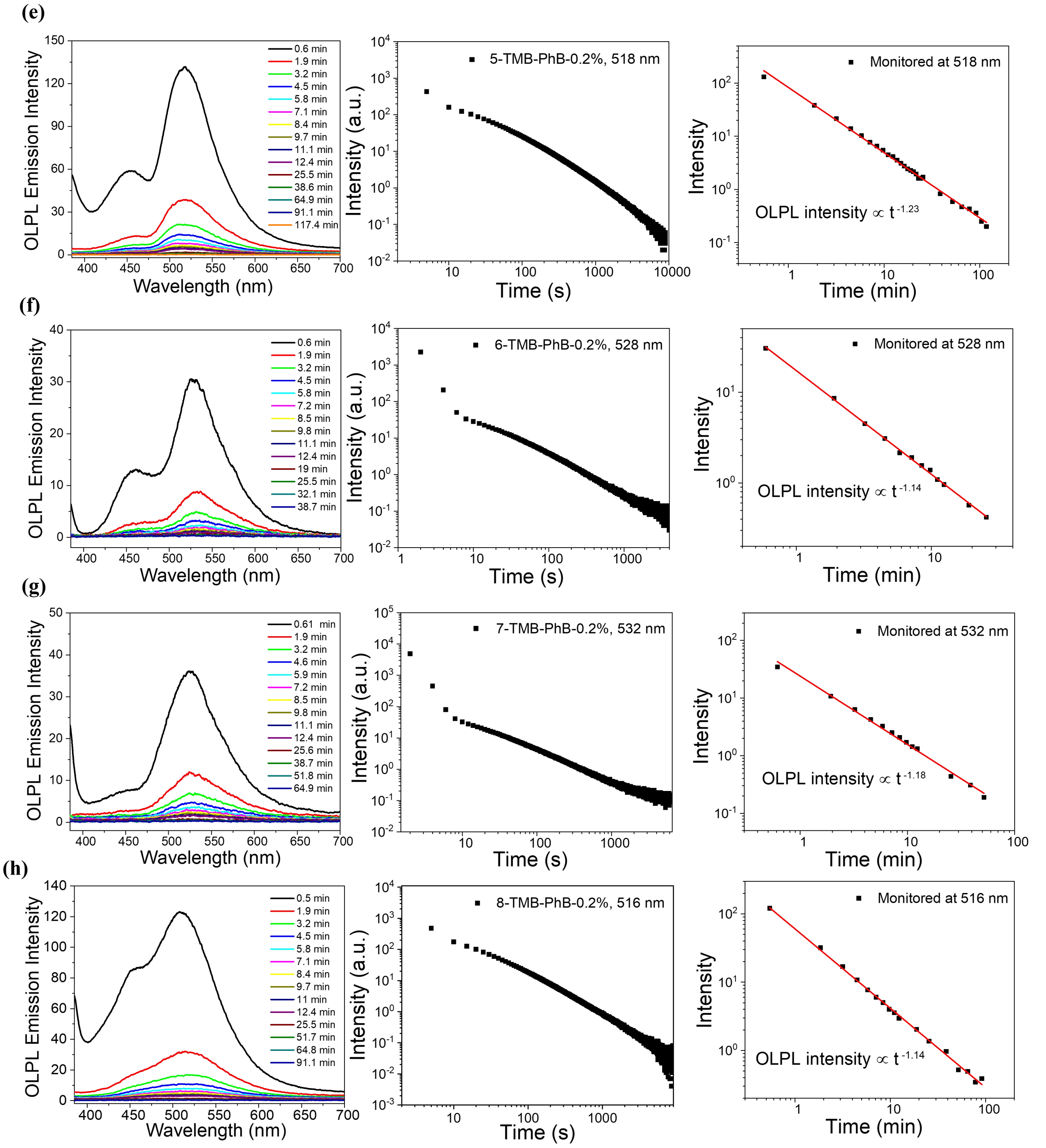

Figure S34. (a-g) The OLPL spectra (left), delayed emission decay profiles (middle) and a logarithmic plot of the OLPL emission decay profile (right) of the **BF$_2$bdk**-TMB-PhB-0.2% materials excited at 365 nm as a function of delay time.

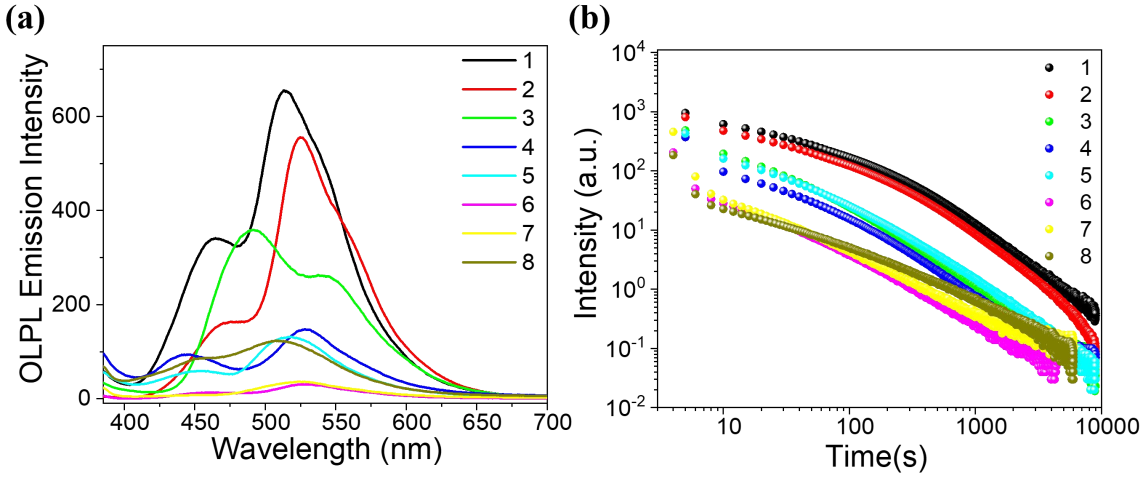

Figure S35. (a) Room-temperature OLPL emission spectra of **BF_2bdk**-TMB-PhB-0.2% samples at delay time of around 0.5 min. (b) Room-temperature emission decay profile of **BF_2bdk**-TMB-PhB-0.2% samples.

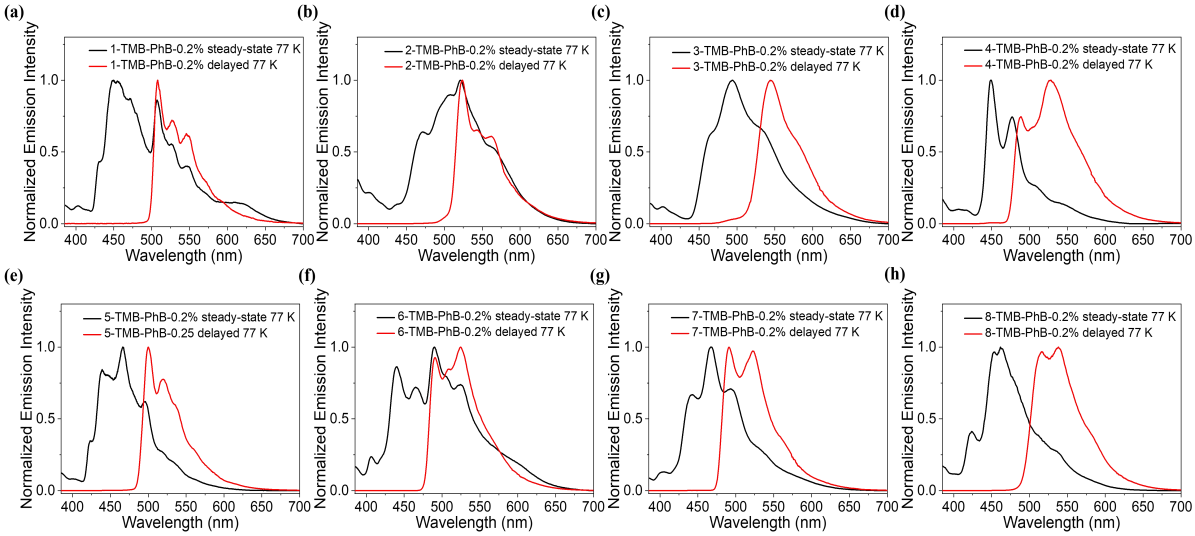

Figure S36. Steady-state and delayed emission spectra of **BF_2bdk**-TMB-PhB-0.2% samples at 77 K excited at 365 nm.

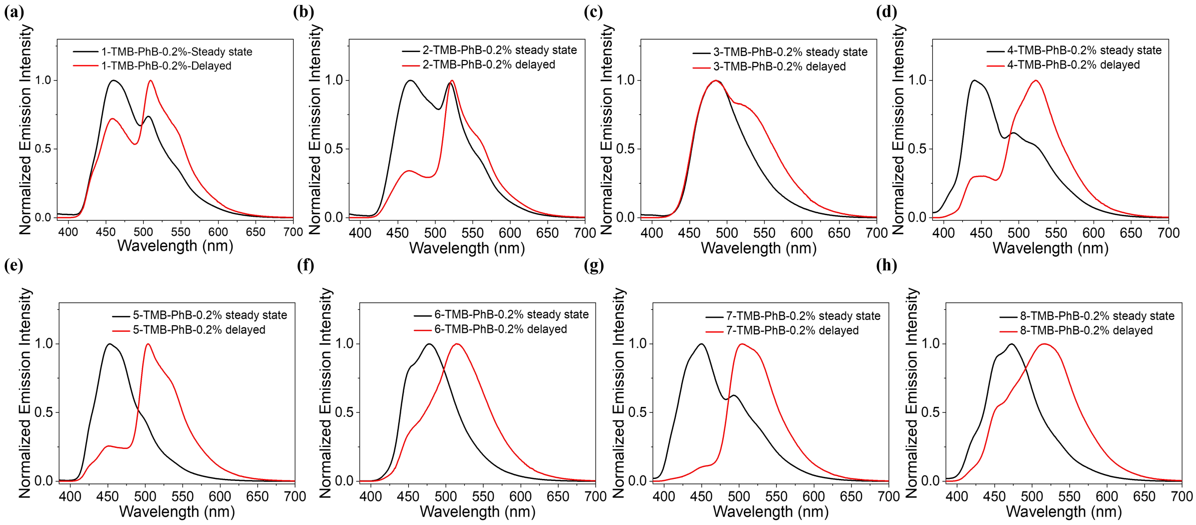

Figure S37. (a-h) Room-temperature steady-state and delayed emission spectra (1 ms delay) of **BF$_2$bdk**-TMB-PhB-0.2% samples excited at 365 nm.

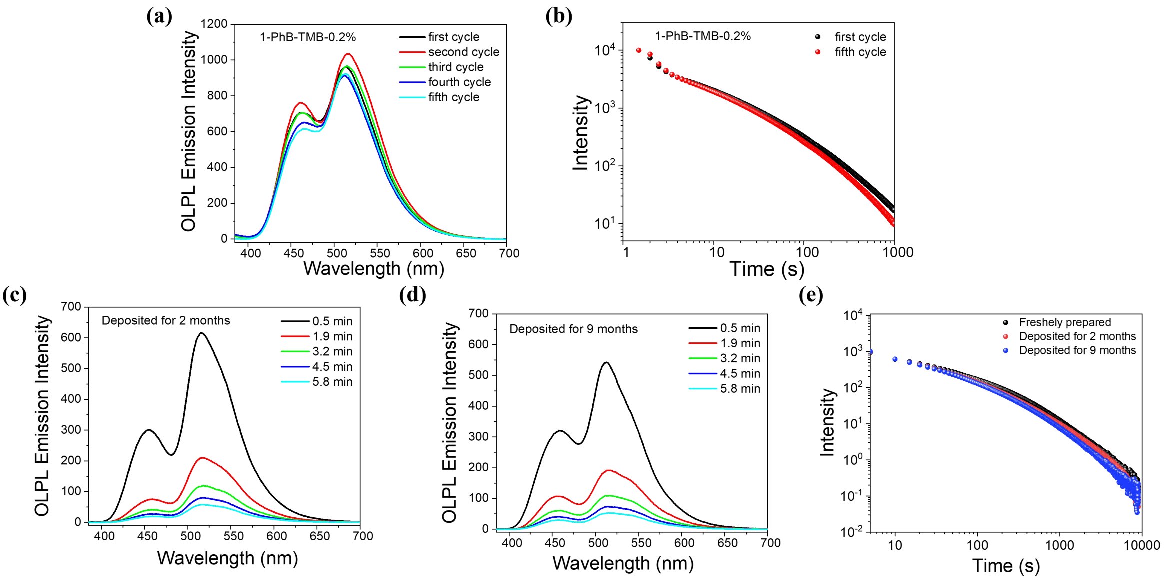

Figure S38. (a) Room-temperature OLPL emission spectra of **1**-TMB-PhB-0.2% samples at delay time of around 0.5 min excited by 365 nm throughout the five cyclic processes. (b) Room-temperature emission decay profile of **1**-TMB-PhB-0.2% samples of first cycle and fifth cycle excited by 365 nm monitored at 514 nm. (c,d) Room-temperature OLPL emission spectra of **1**-TMB-PhB-0.2% samples deposited for 2 months and 9 months excited by 365 nm. (e) Room-temperature emission decay profile of freshly prepared, deposited for 2 months and deposited for 9 months **1**-TMB-PhB-0.2% samples excited by 365 nm monitored at 514 nm.

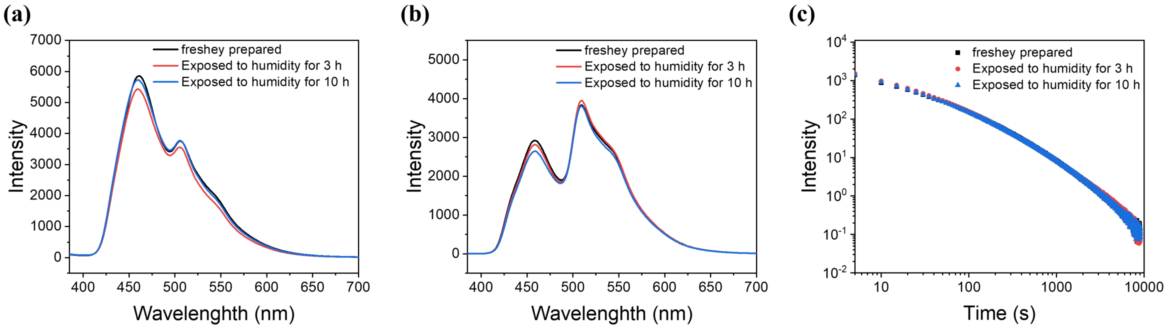

Figure S39. (a) Room-temperature steady-state, (b) delayed emission spectra (1 ms delay) and (c) emission decay profile (monitored at 514 nm) of the same 1-TMB-PhB-0.2% sample under three conditions: freshly prepared, exposed to humidity for 3 hours, and exposed to humidity for 10 hours.

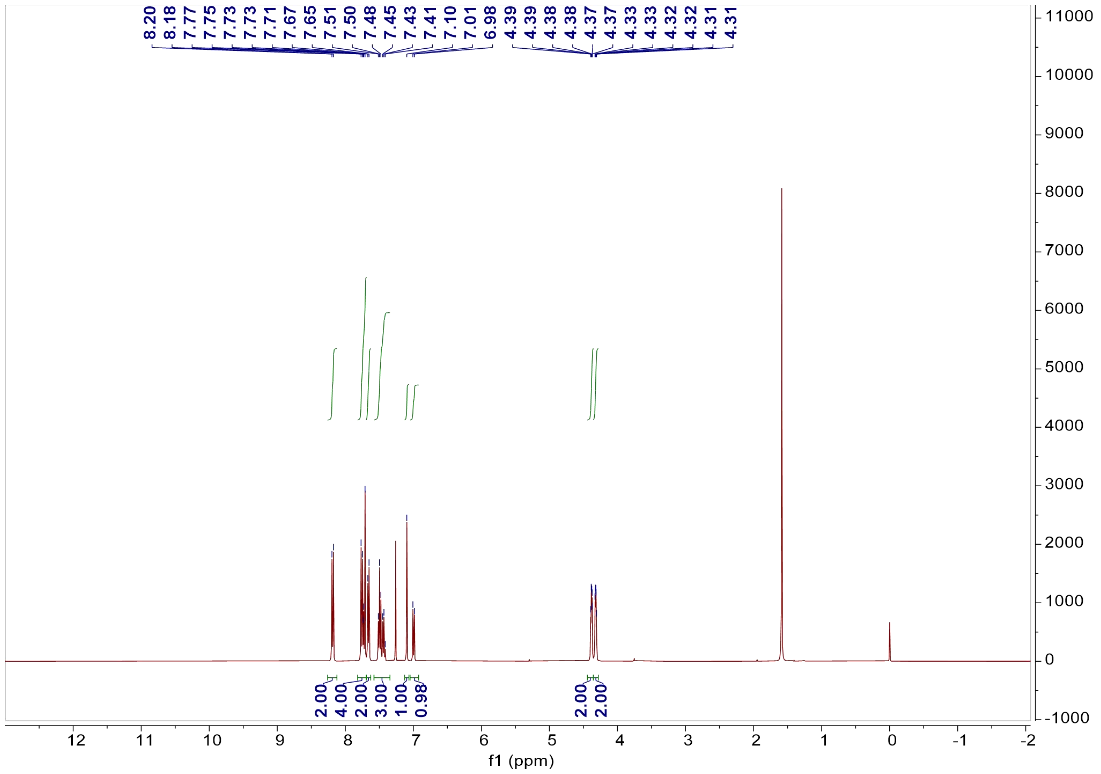

Figure S40. ^{1}H NMR spectrum of compound **1**.

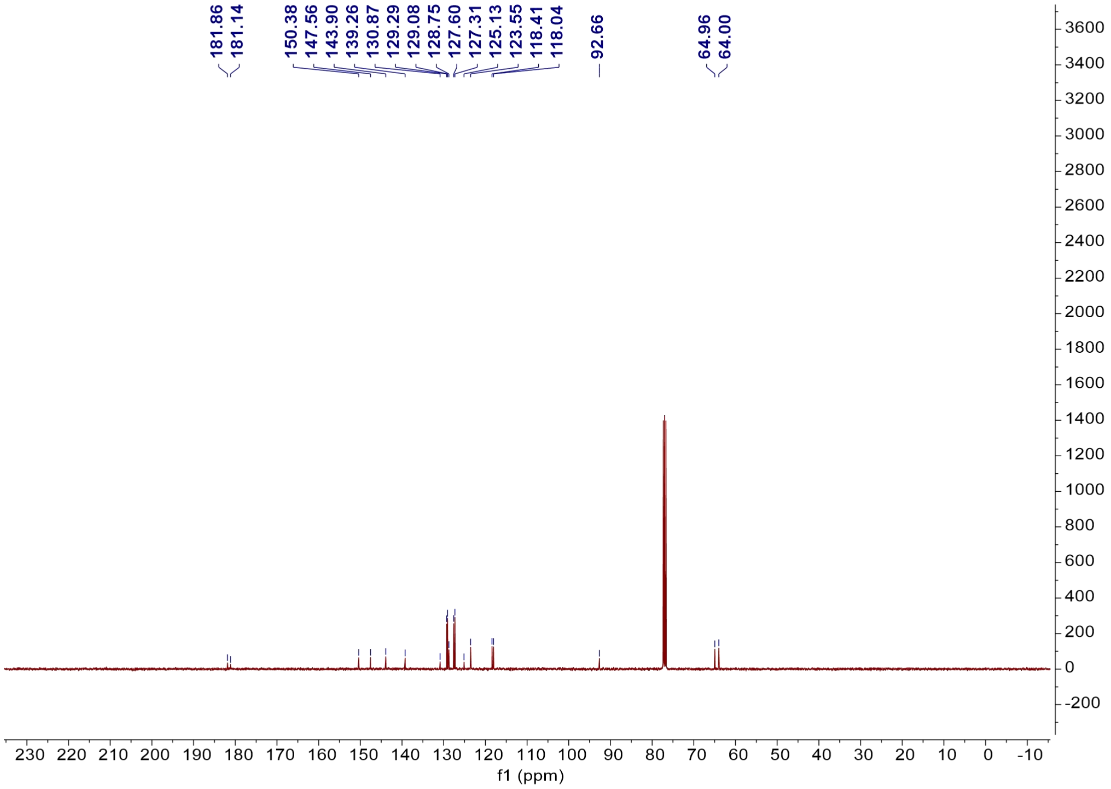

Figure S41. ^{13}C NMR spectrum of compound **1**.

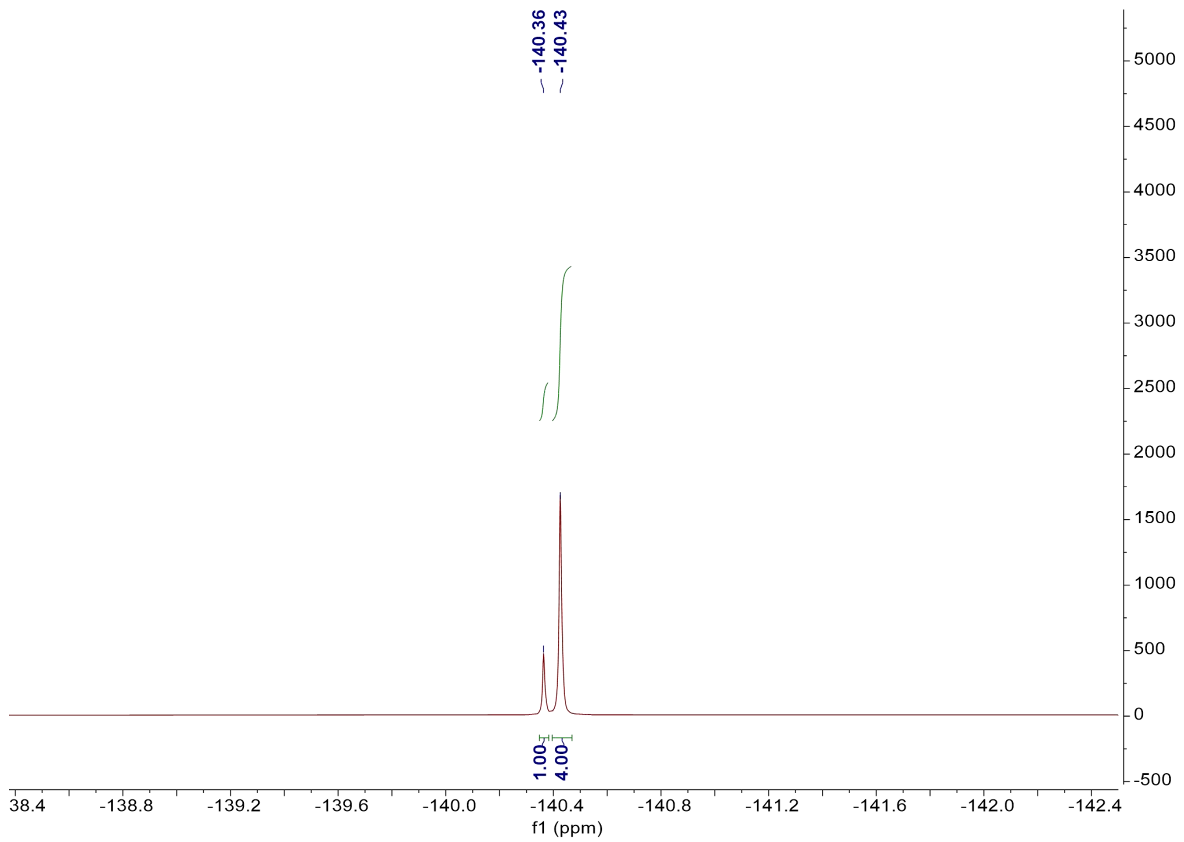

Figure S42. ^{19}F NMR spectrum of compound **1.**

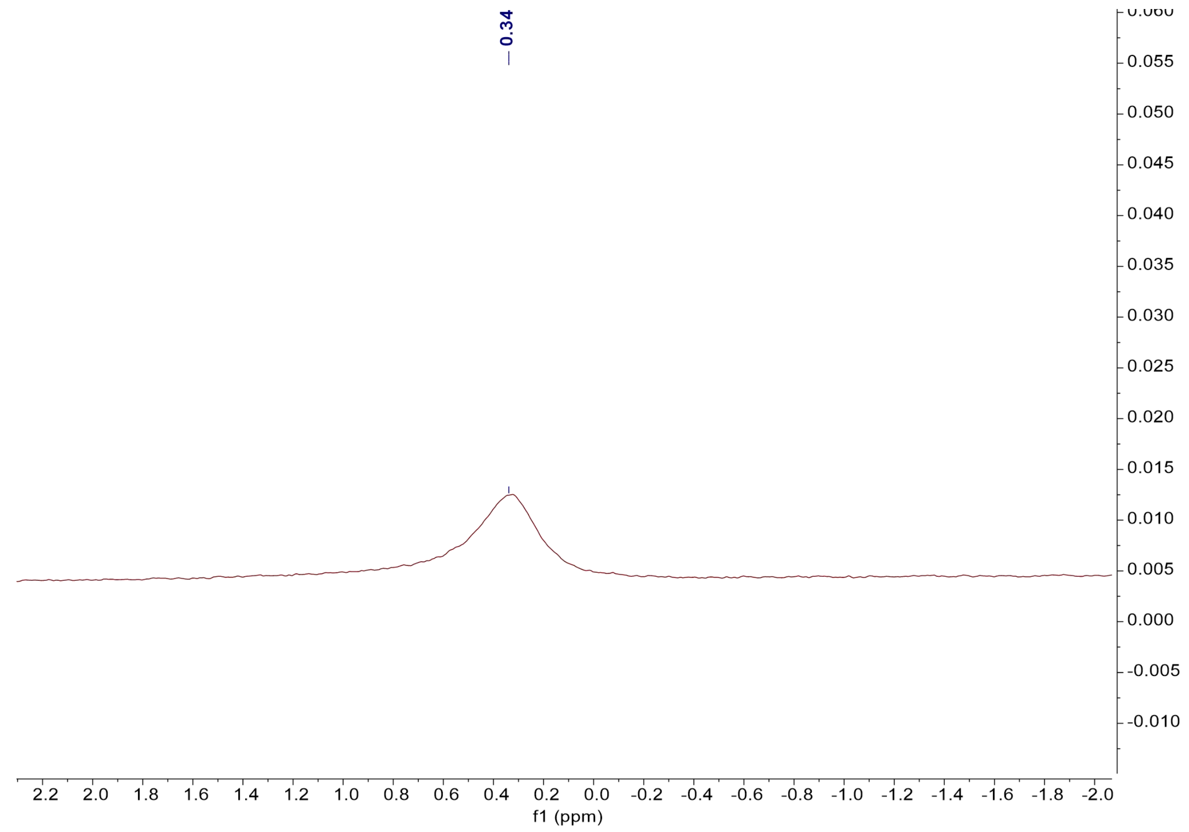

Figure S43. ^{11}B NMR spectrum of compound **1.**

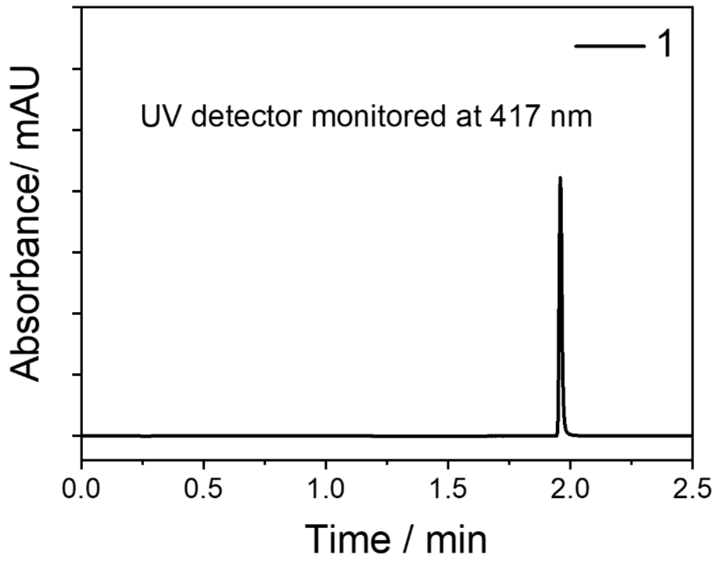

Figure S44. HPLC spectrum of compound 1 UV absorption monitored at 417 nm.

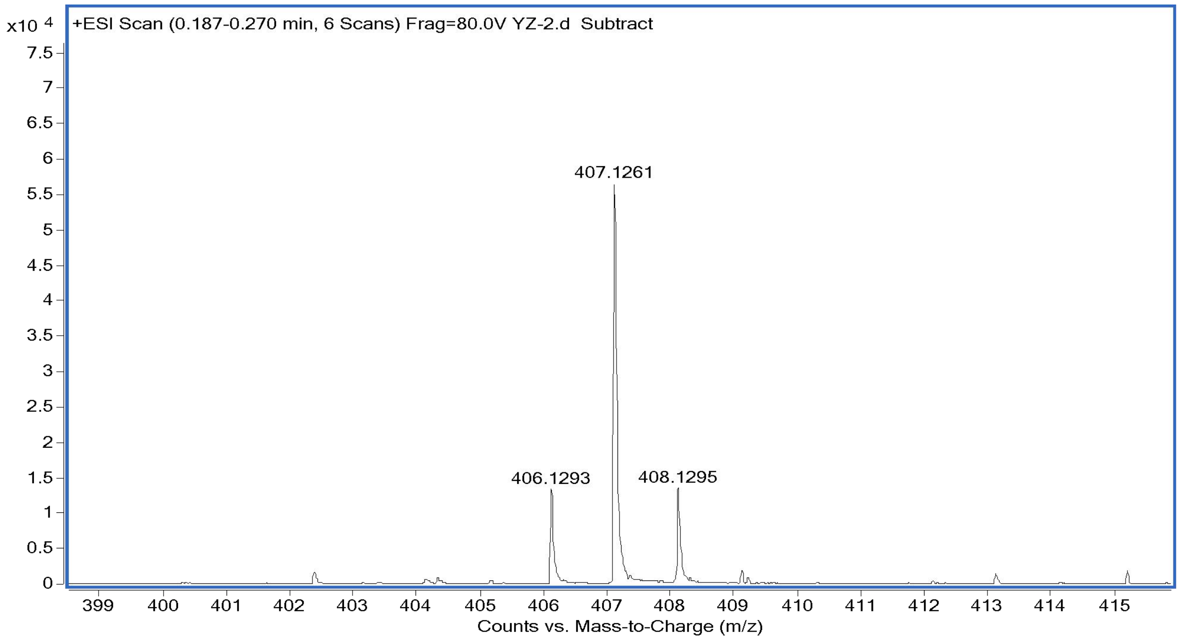

Figure S45. HRMS spectrum of compound **1**.

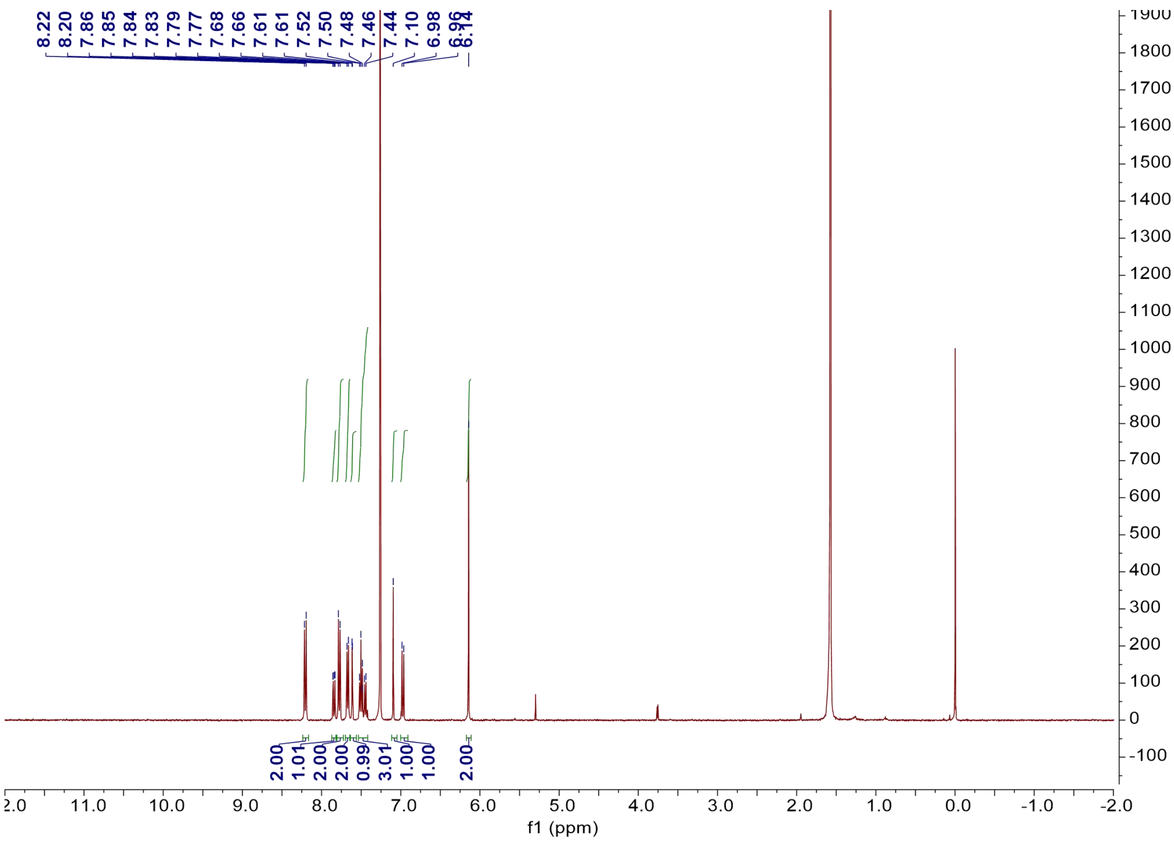

Figure S46. ^{1}H NMR spectrum of compound **2**.

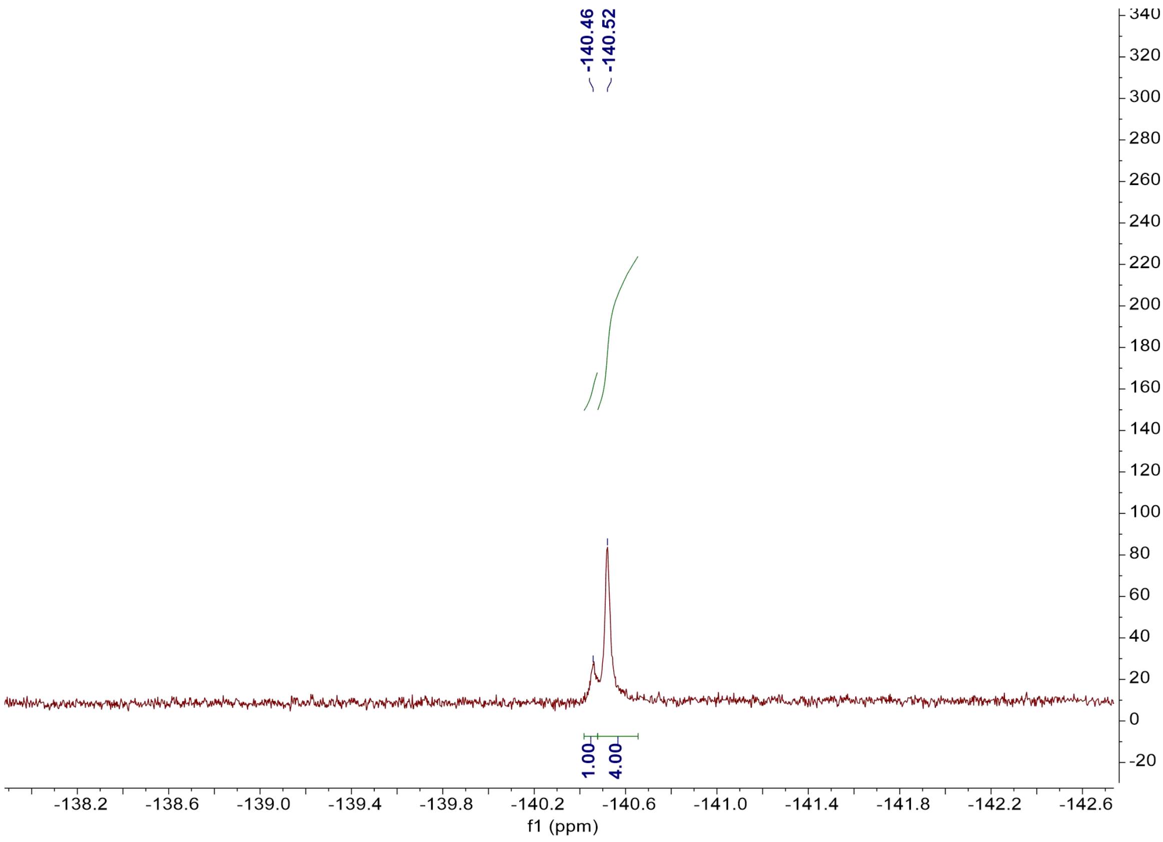

Figure S47. ^{19}F NMR spectrum of compound

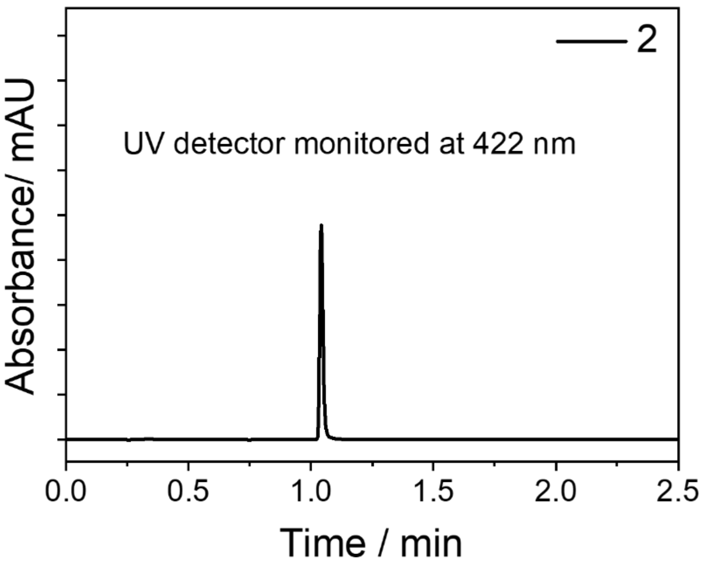

2.

Figure S48. HPLC spectrum of compound **2** UV absorption monitored at 422 nm.

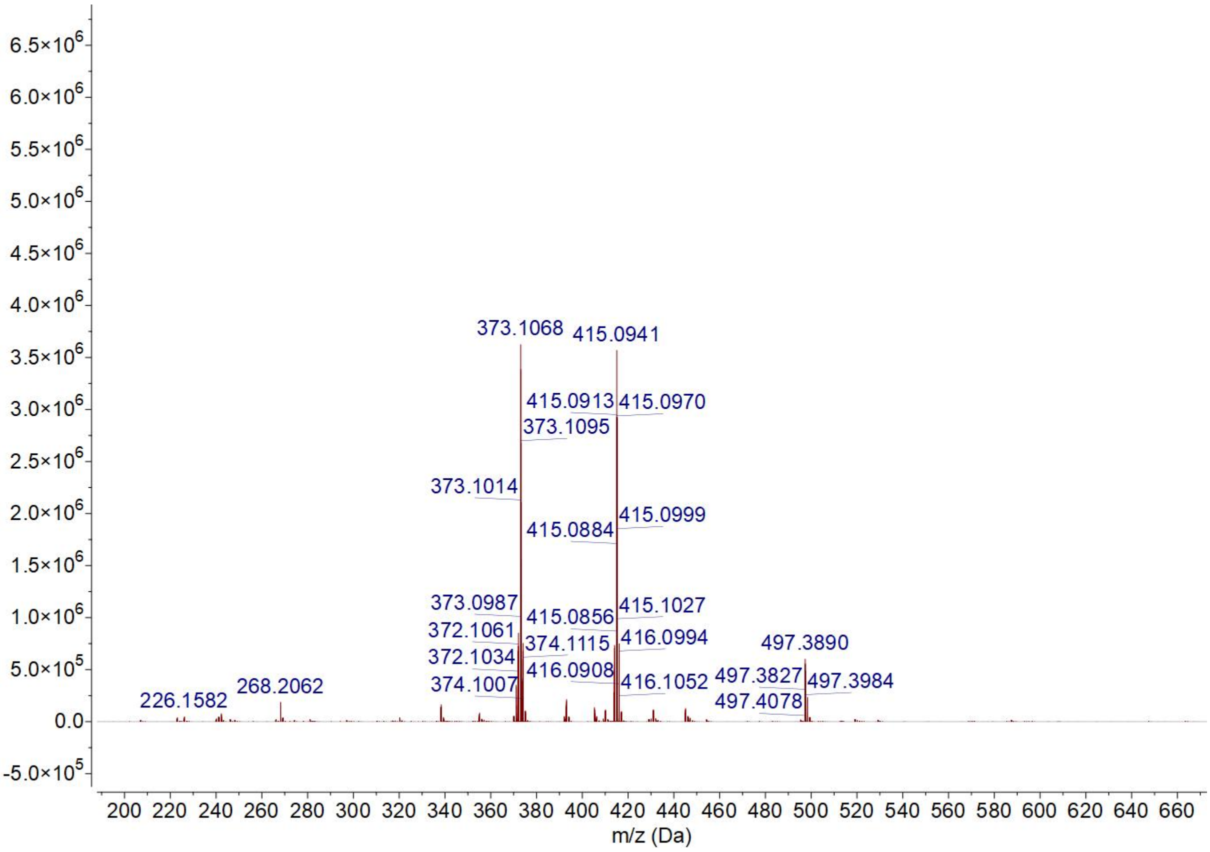

Figure S49. HRMS spectrum of compound **2**.

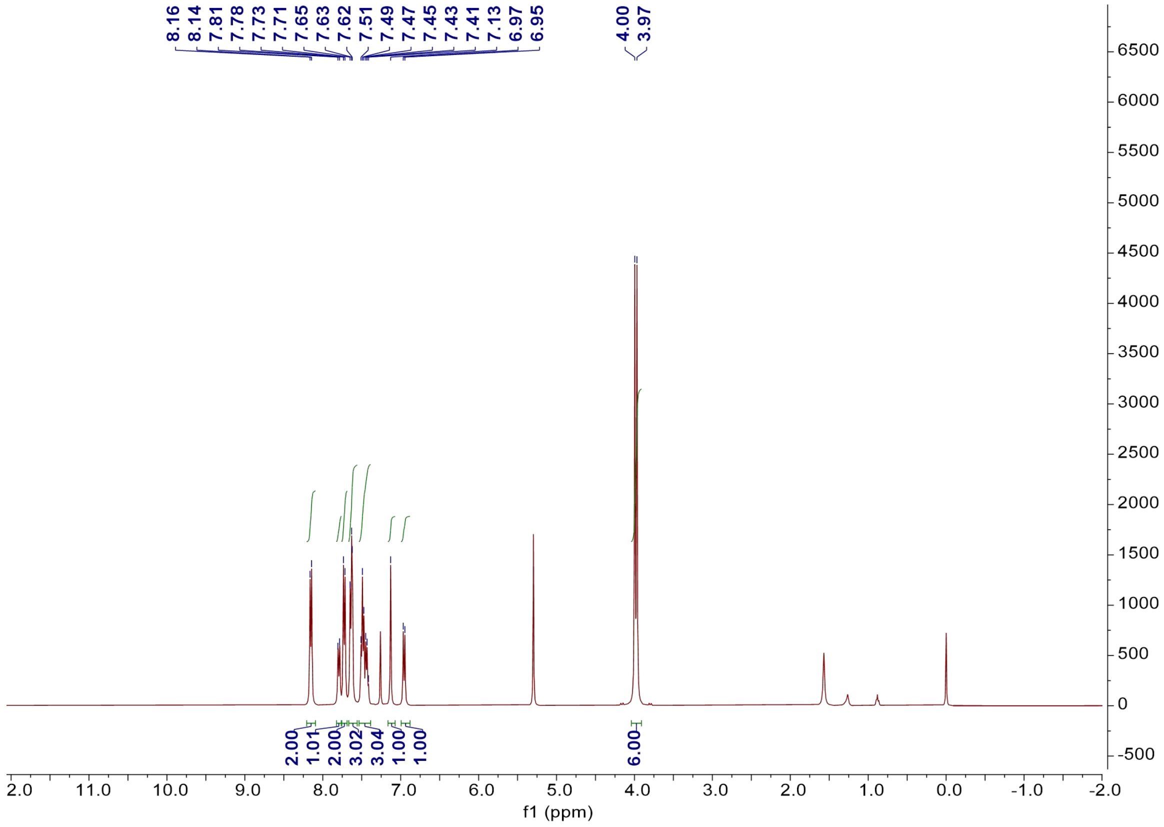

Figure S50. ^{1}H NMR spectrum of compound **3**.

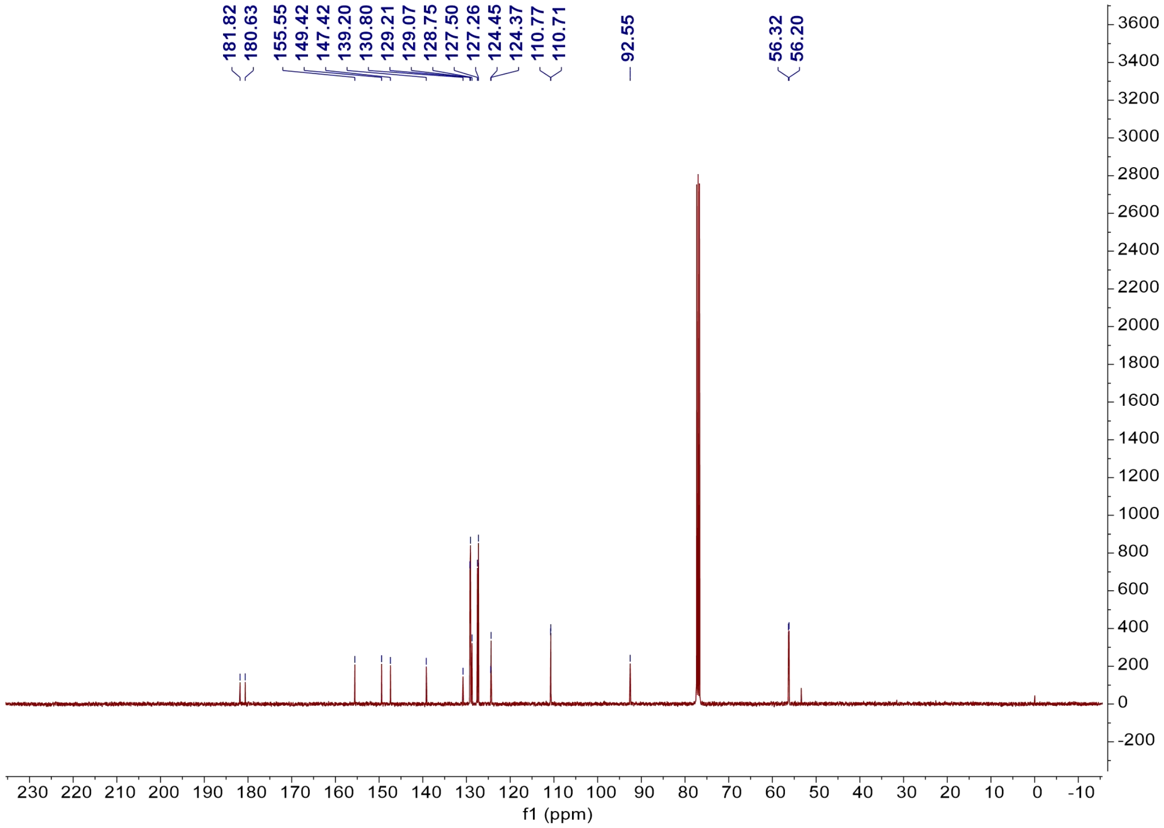

Figure S51. ^{13}C NMR spectrum of compound **3**.

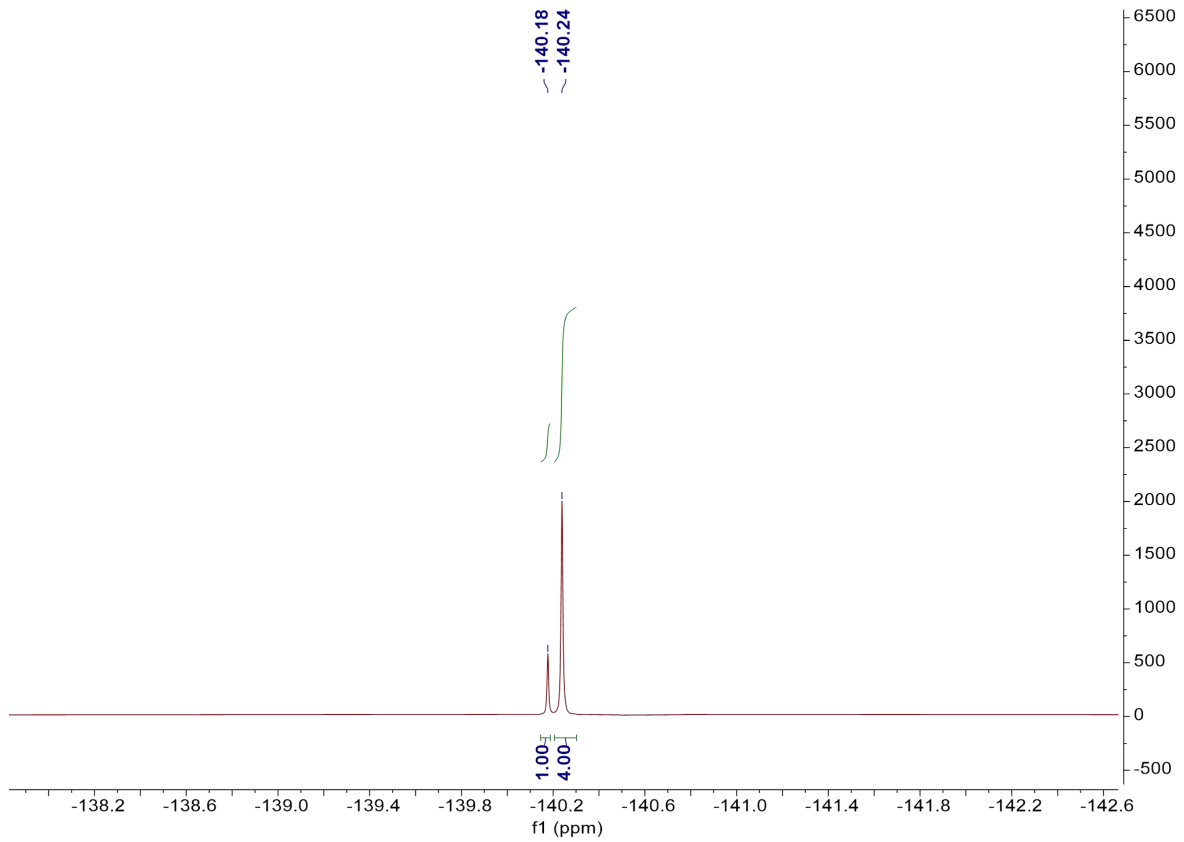

Figure S52. ^{19}F NMR spectrum of compound **3**.

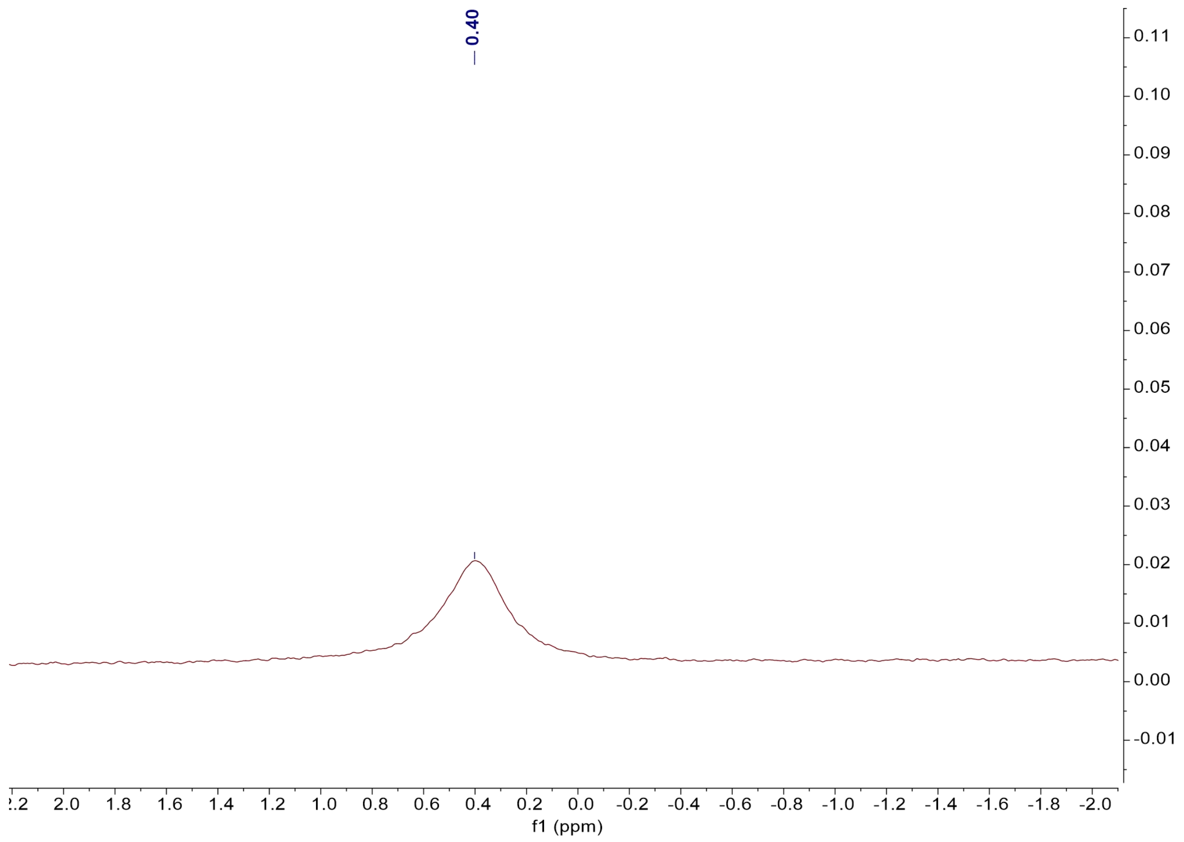

Figure S53. ^{11}B NMR spectrum of compound **3**.

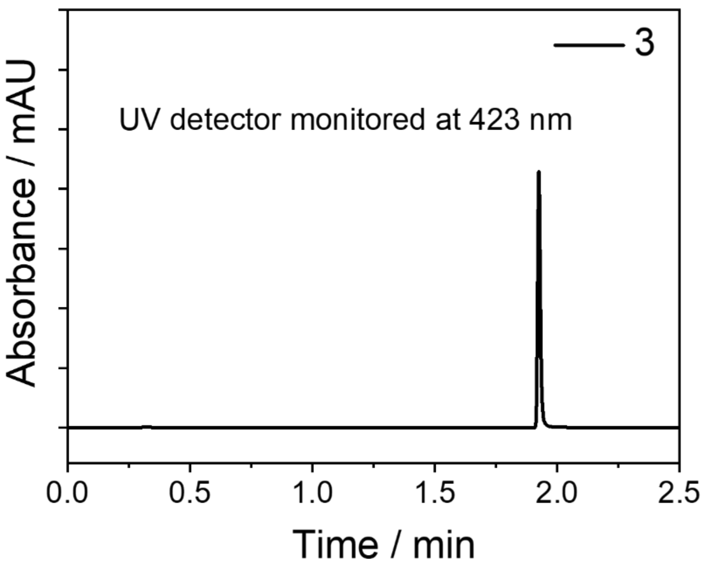

Figure S54. HPLC spectrum of compound **3** UV absorption monitored at 423 nm.

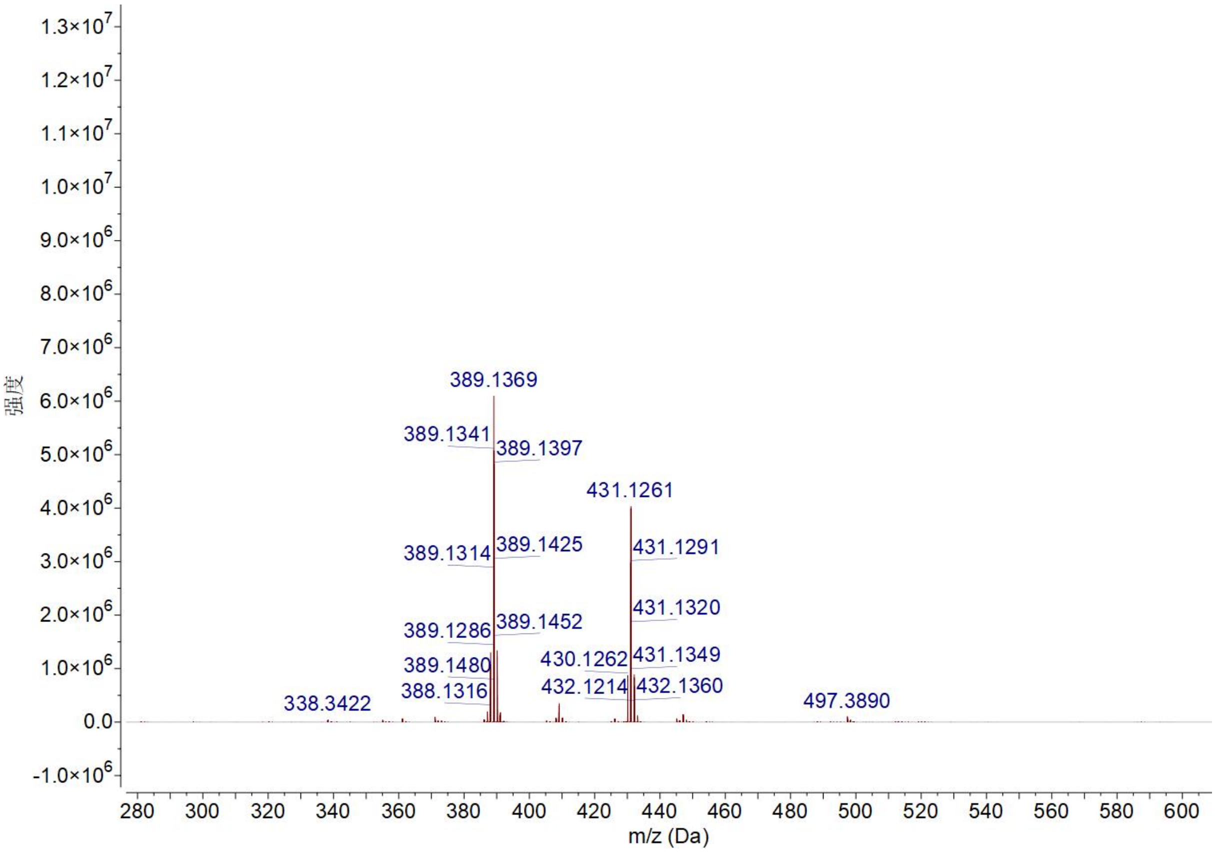

Figure S55. HRMS spectrum of compound **3**.

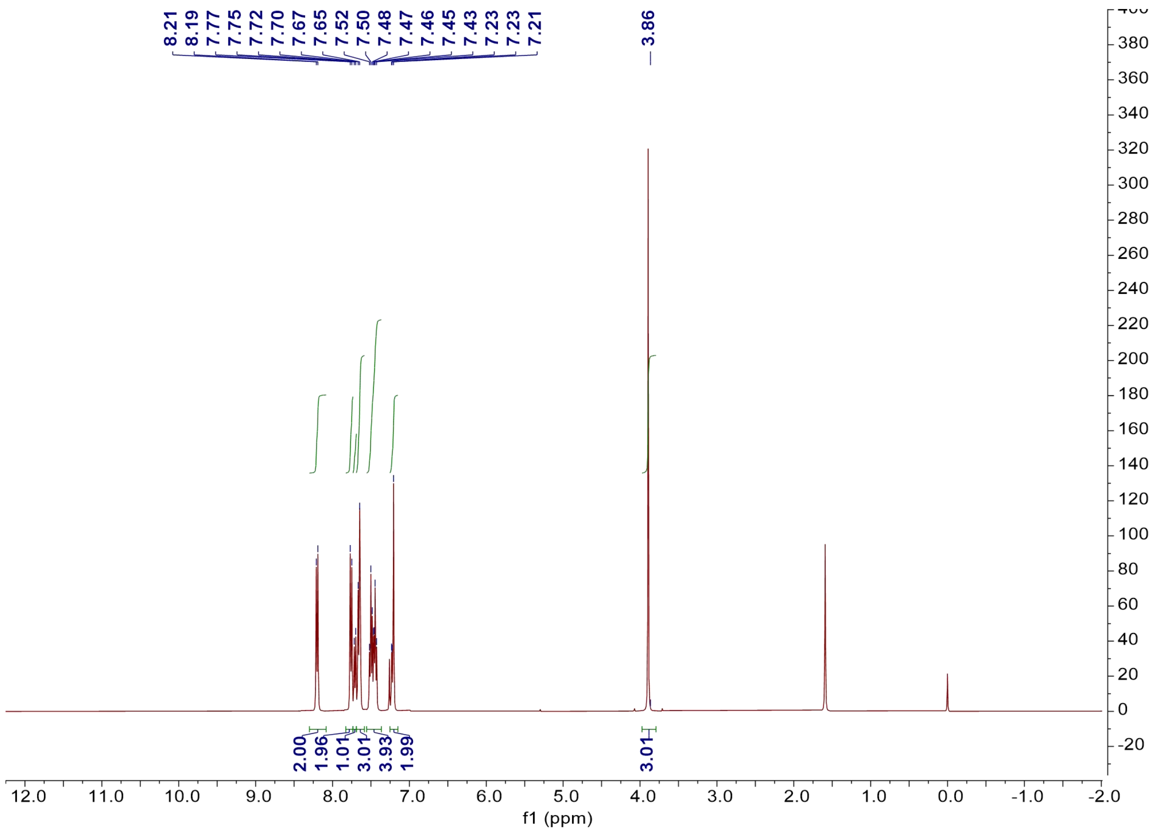

Figure S56. ^{1}H NMR spectrum of compound **4**.

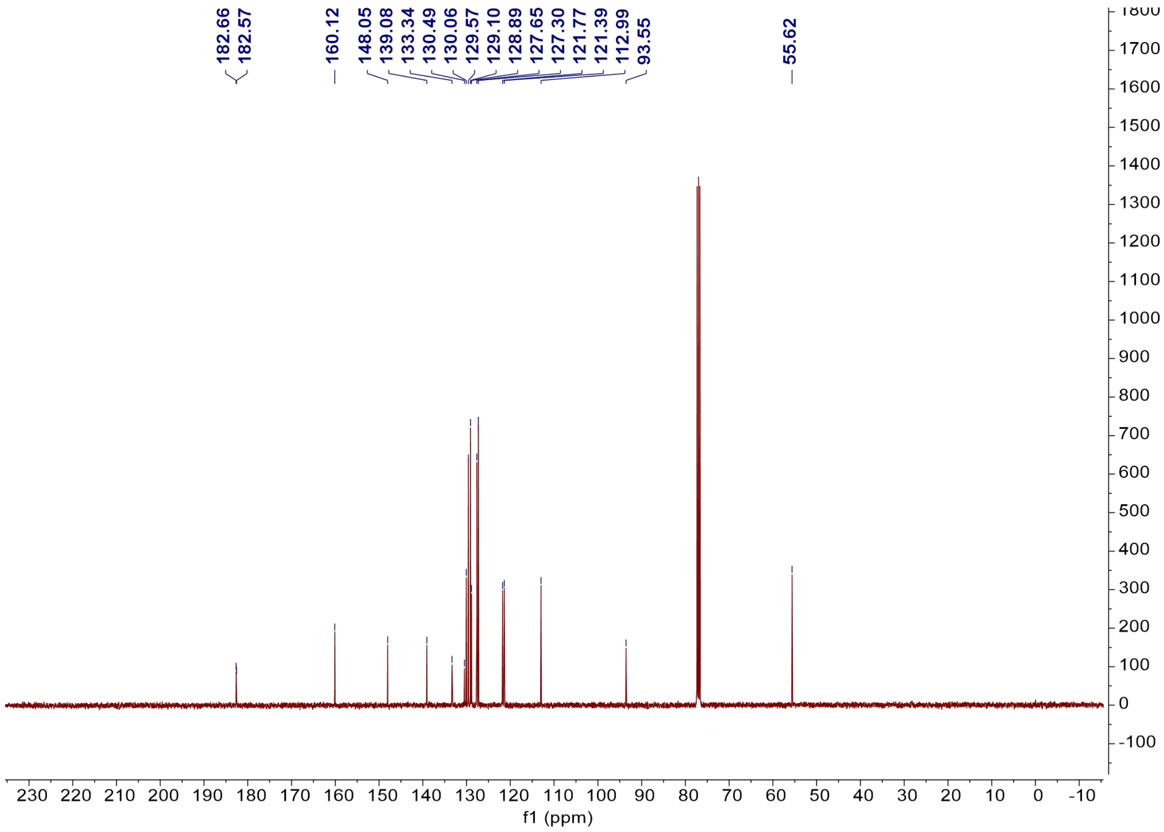

Figure S57. ^{13}C NMR spectrum of compound **4**.

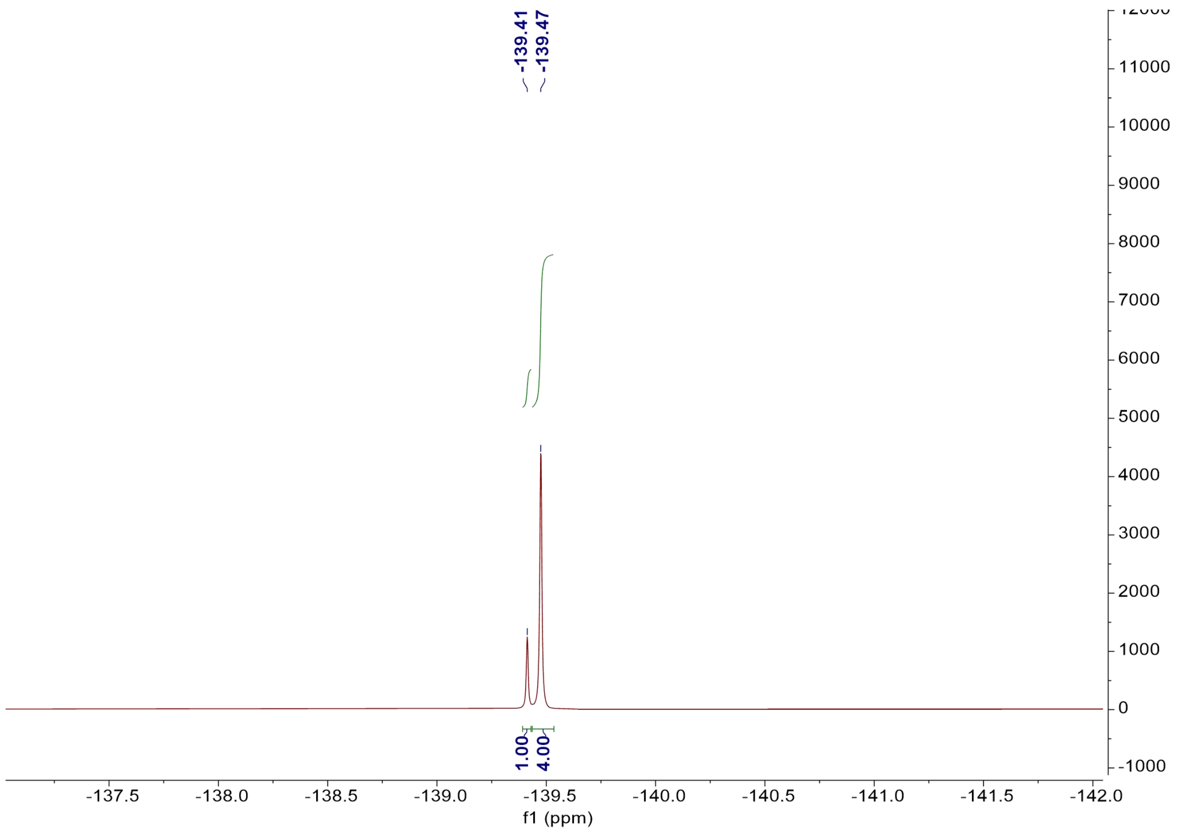

Figure S58. ^{19}F NMR spectrum of compound **4**.

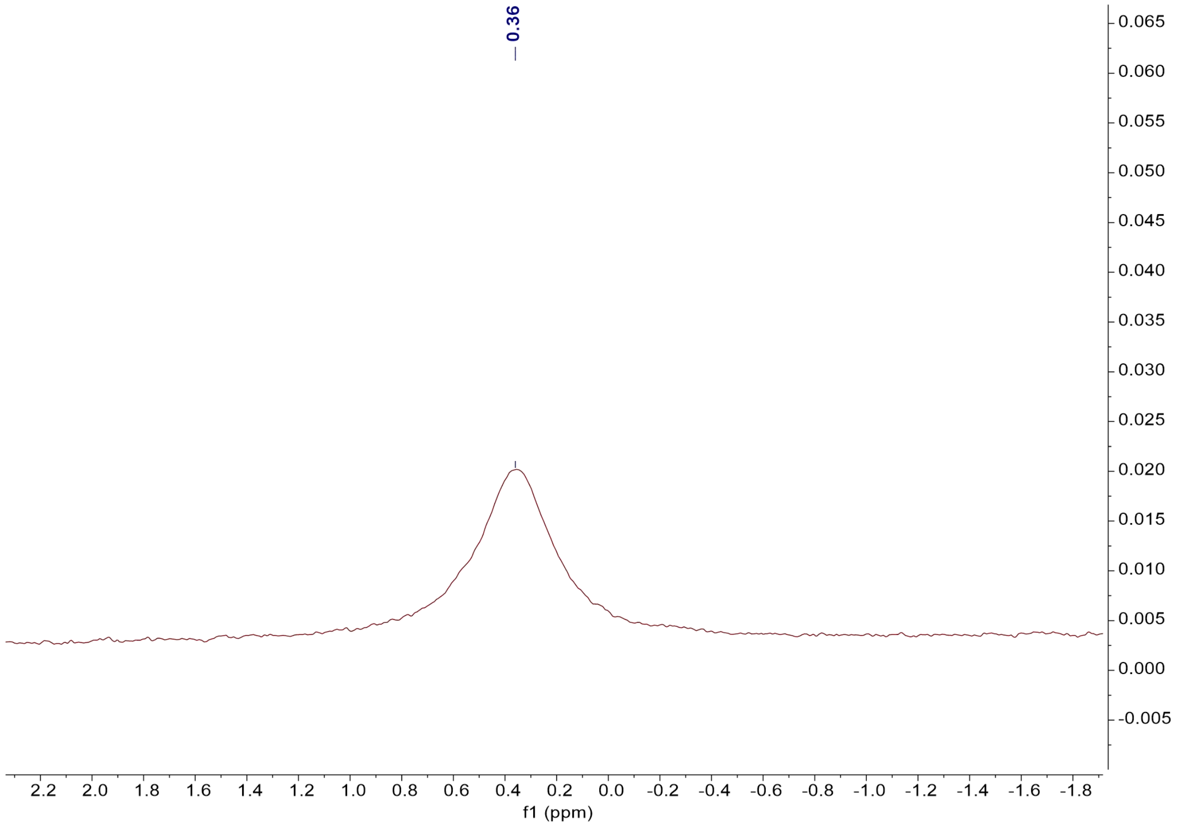

Figure S59. ^{11}B NMR spectrum of compound **4**.

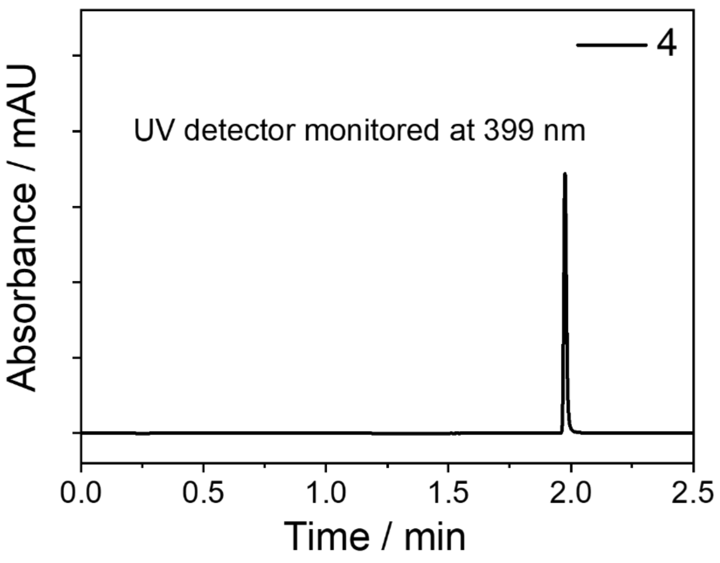

Figure S60. HPLC spectrum of compound **4** UV absorption monitored at 399 nm.

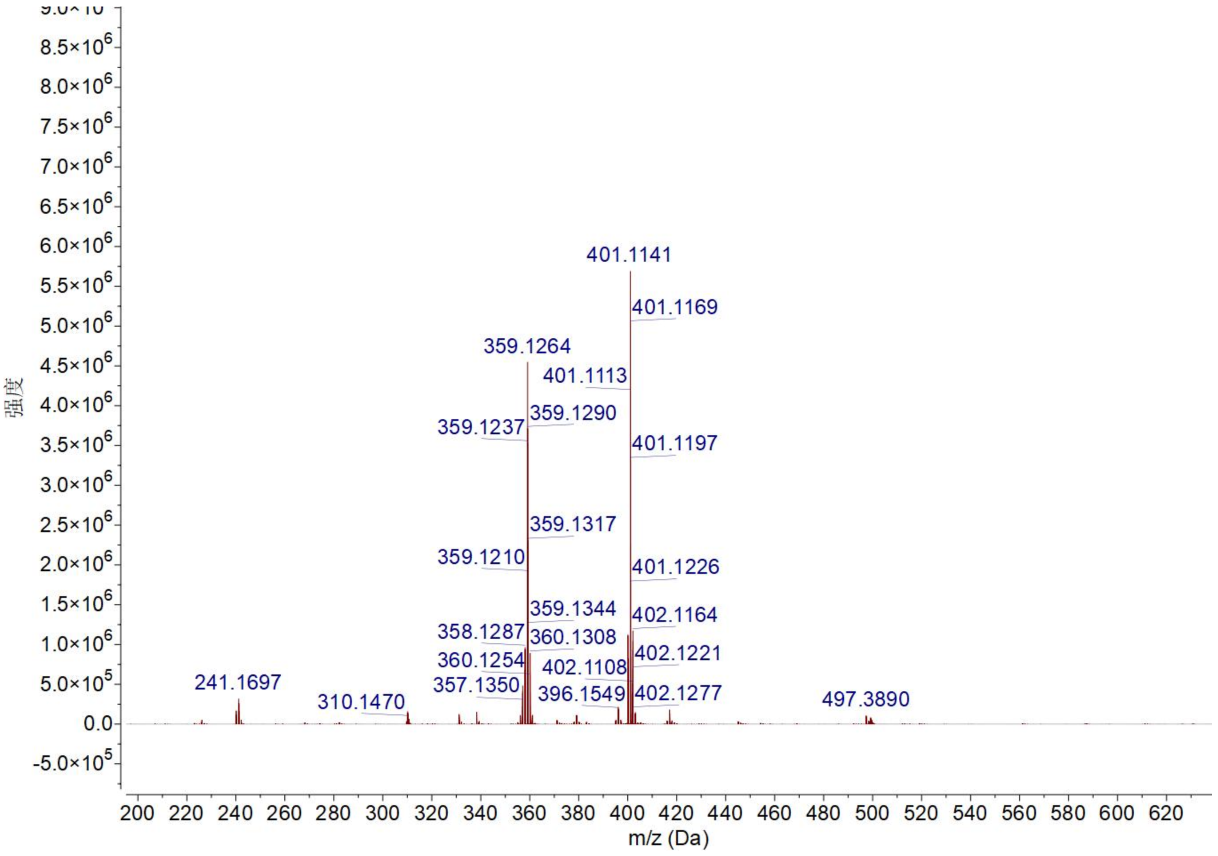

Figure S61. HRMS spectrum of compound **4**.

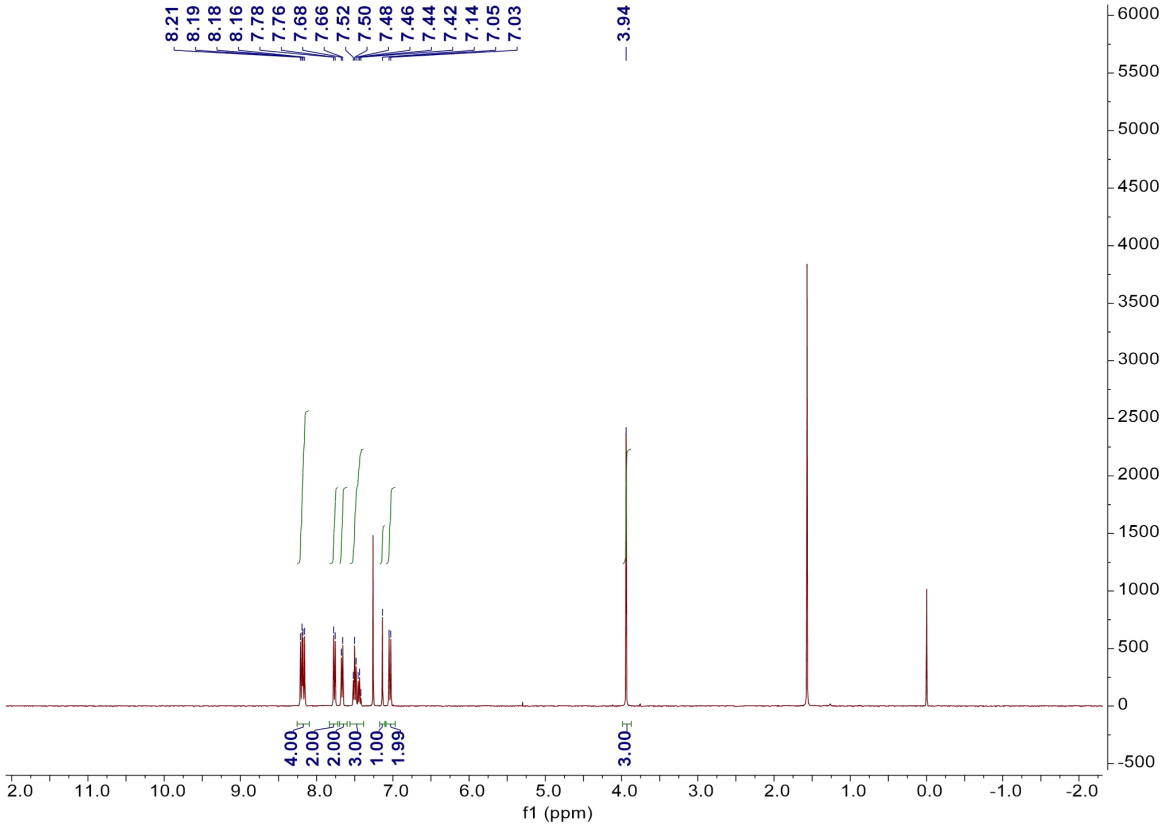

Figure S62. ^{19}F NMR spectrum of compound **5**.

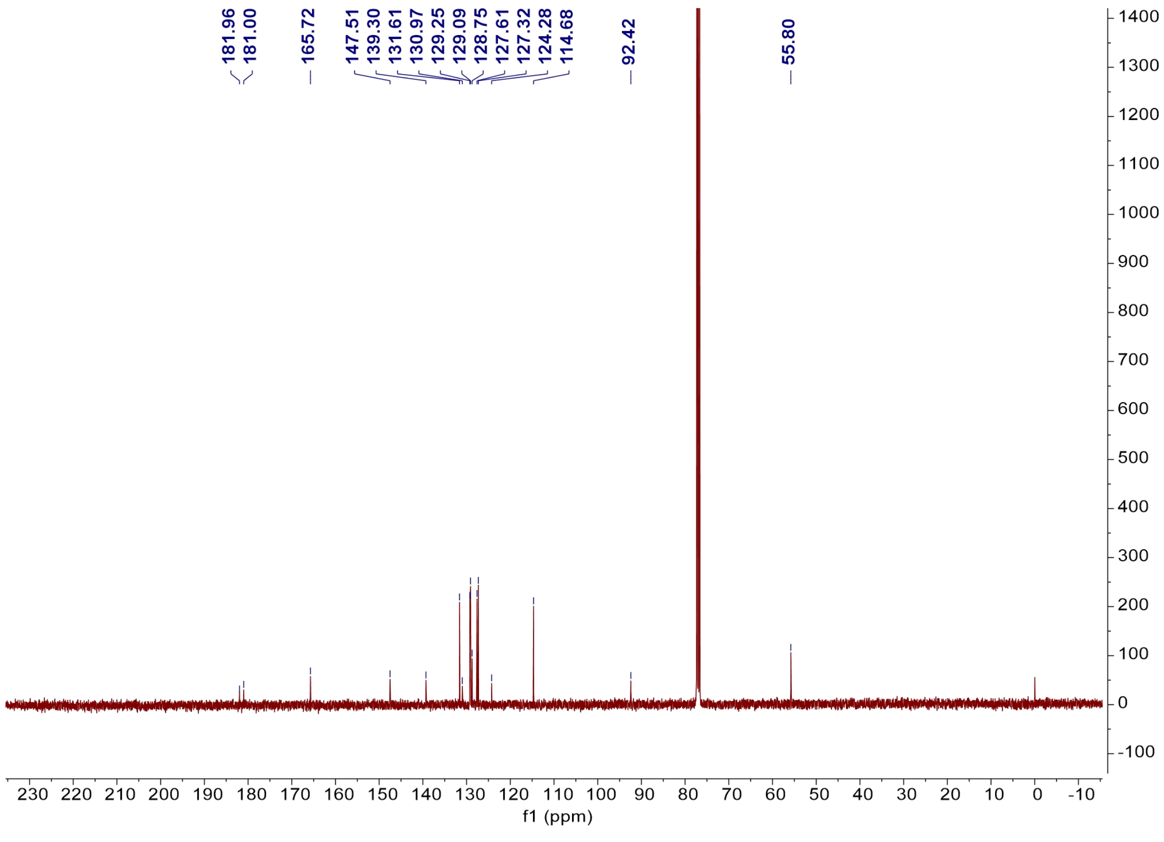

Figure S63. ^{13}C NMR spectrum of compound **5**.

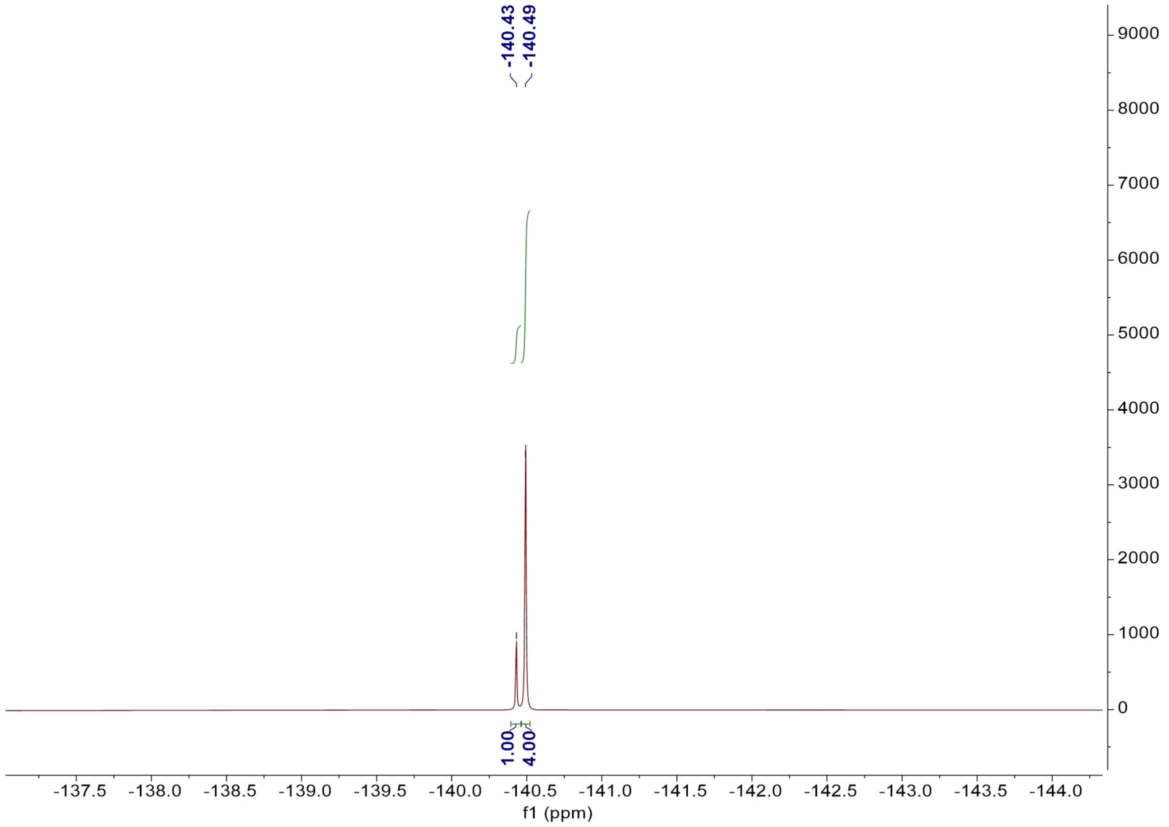

Figure S64. ^{19}F NMR spectrum of compound **5**.

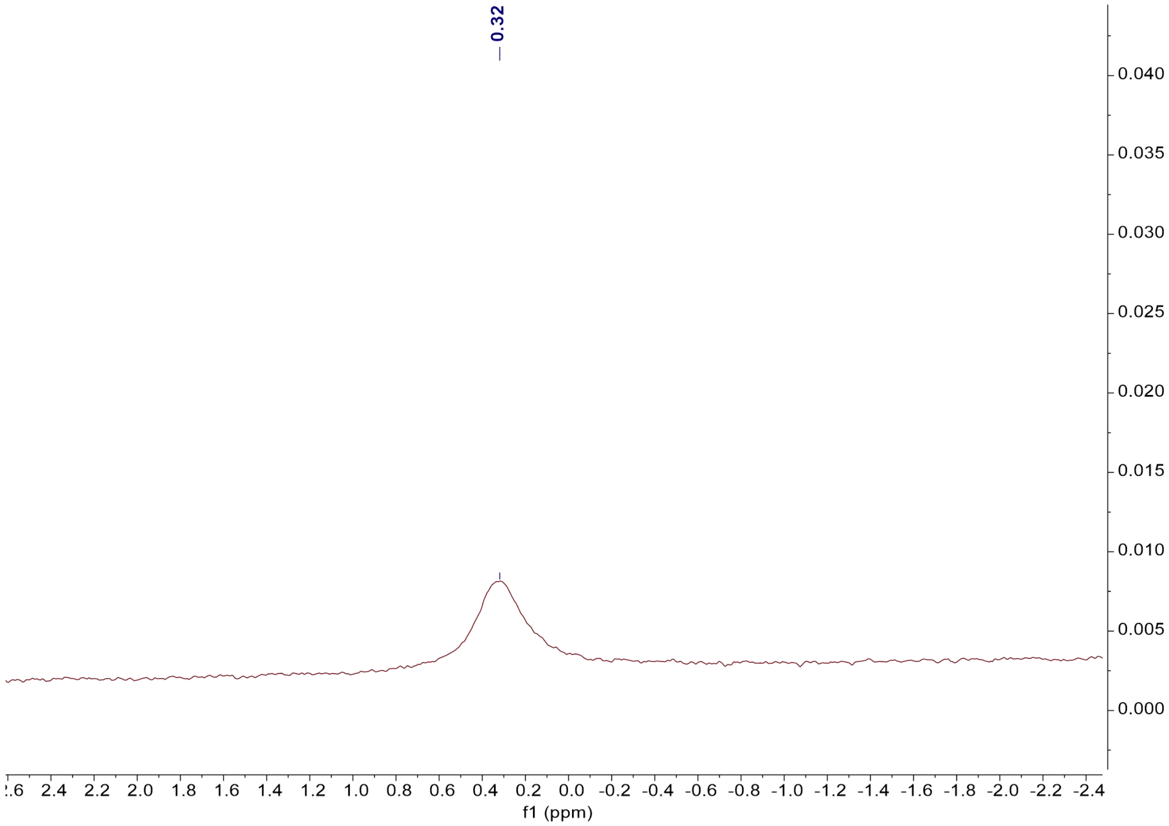

Figure S65. ^{11}B NMR spectrum of compound **5**.

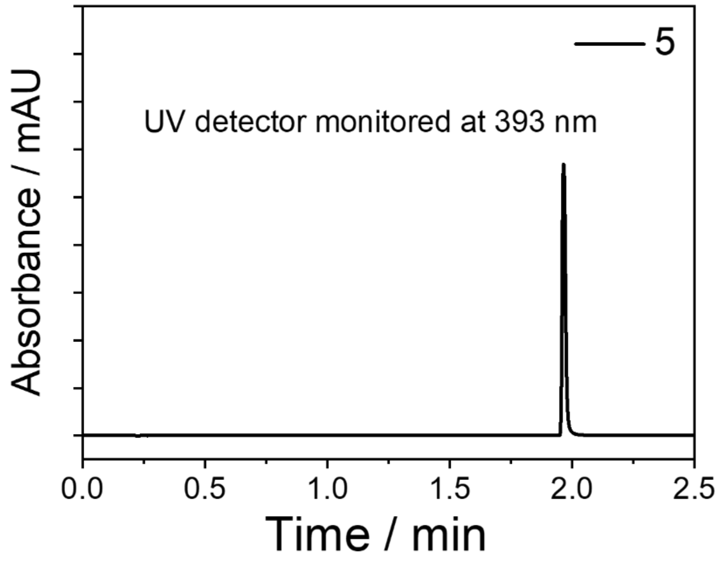

Figure S66. HPLC spectrum of compound **5** UV absorption monitored at 393 nm.

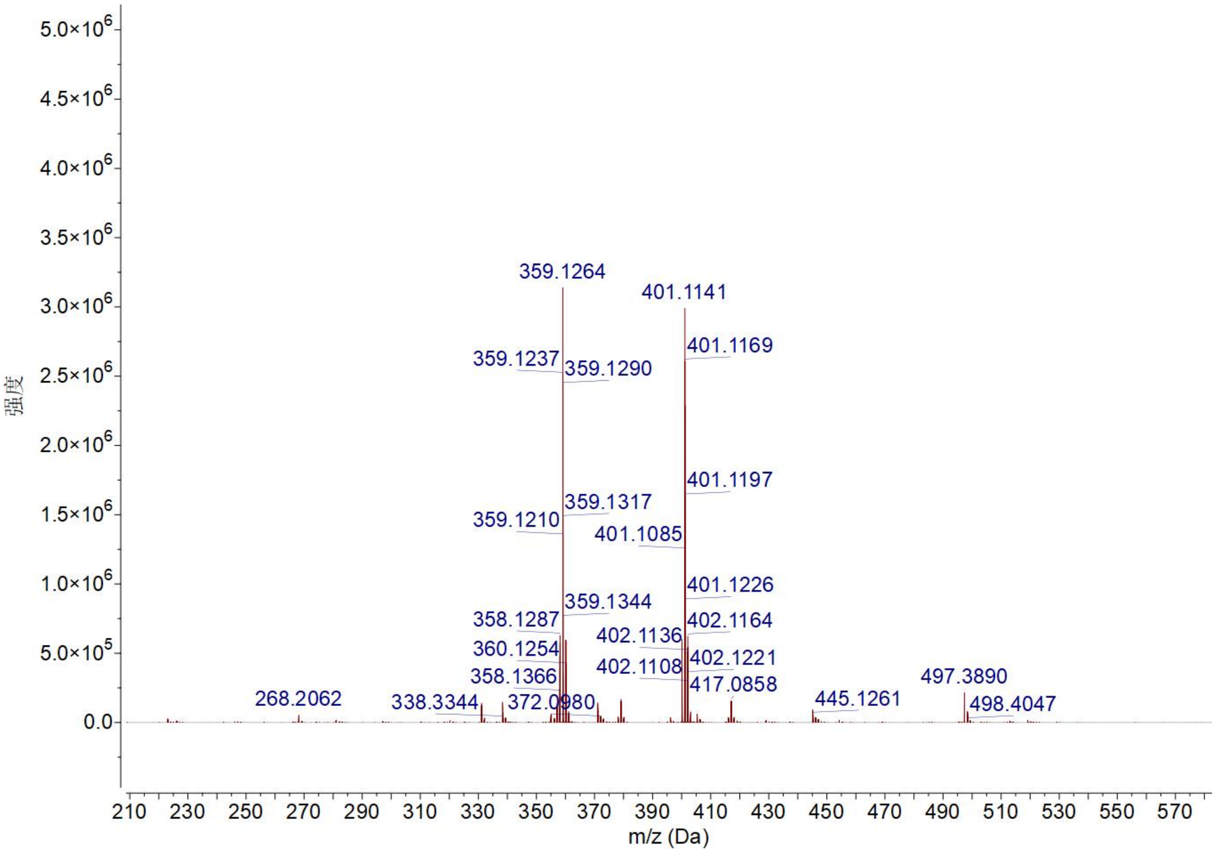

Figure S67. HRMS spectrum of compound **5**.

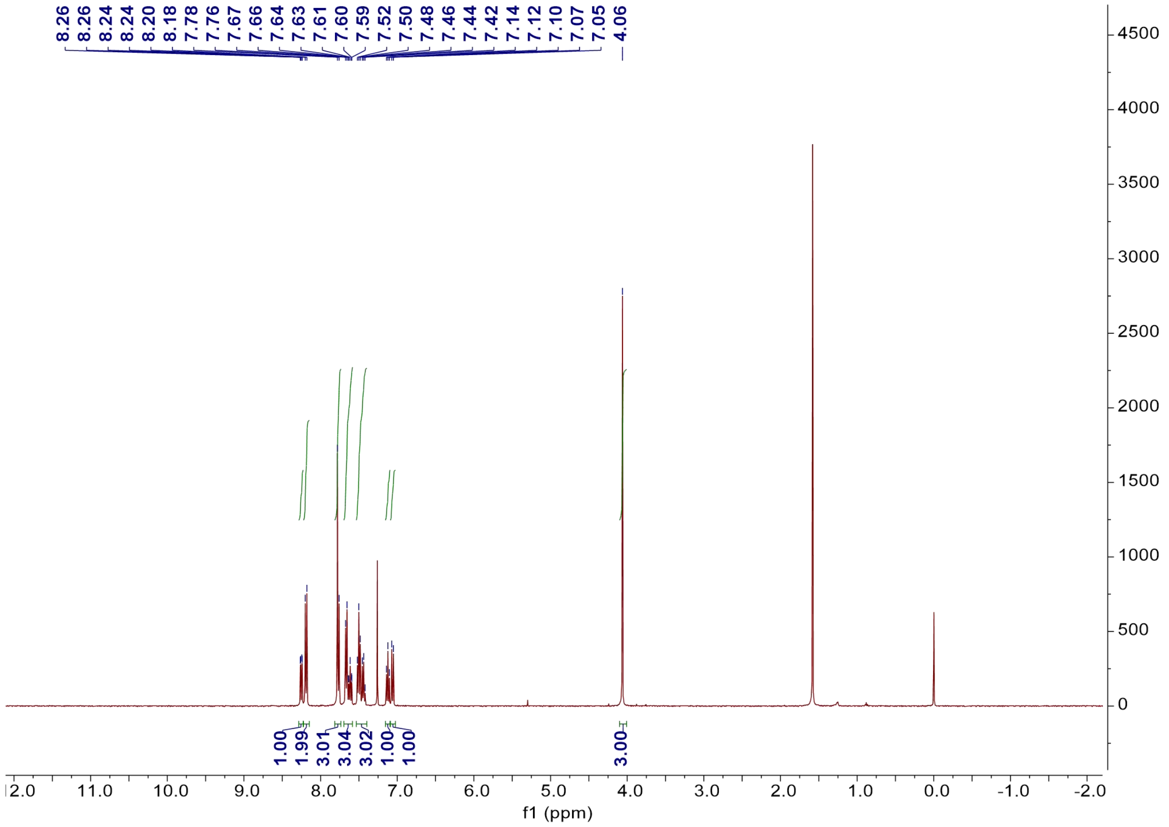

Figure S68. ^{1}H NMR spectrum of compound **6.**

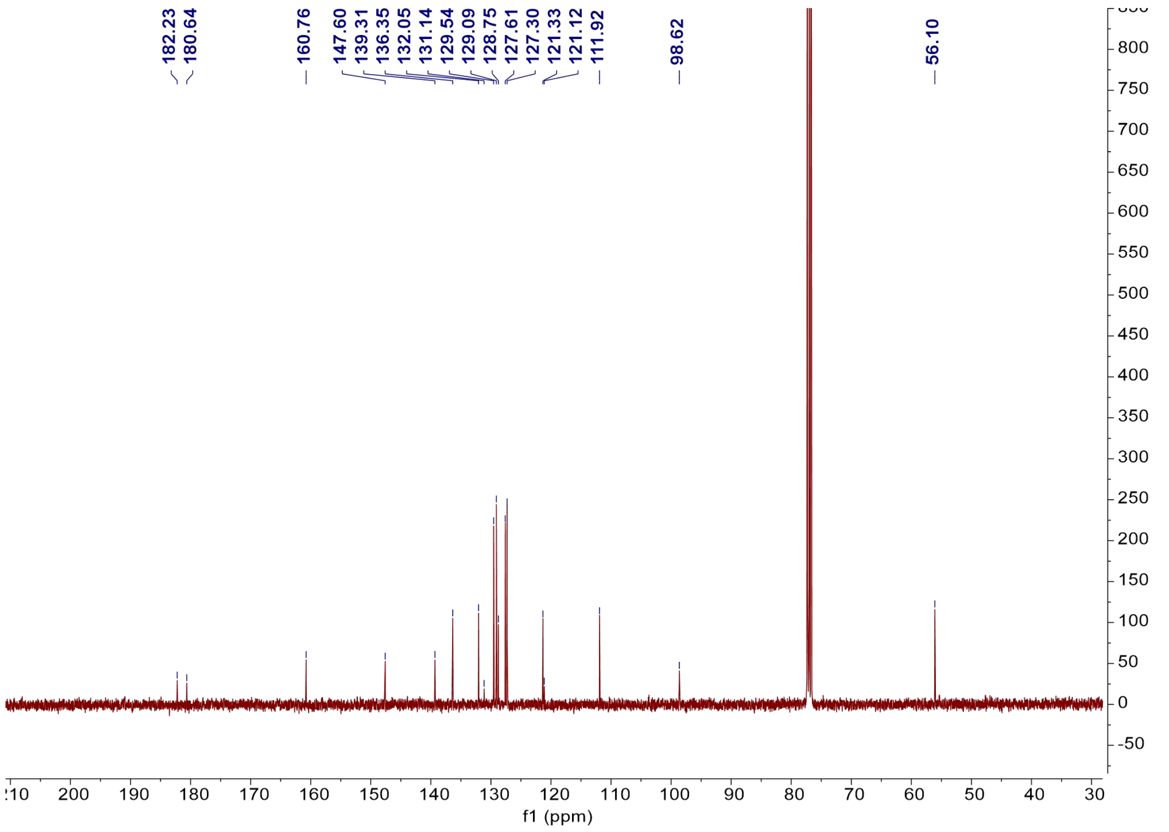

Figure S69. ^{13}C NMR spectrum of compound **6.**

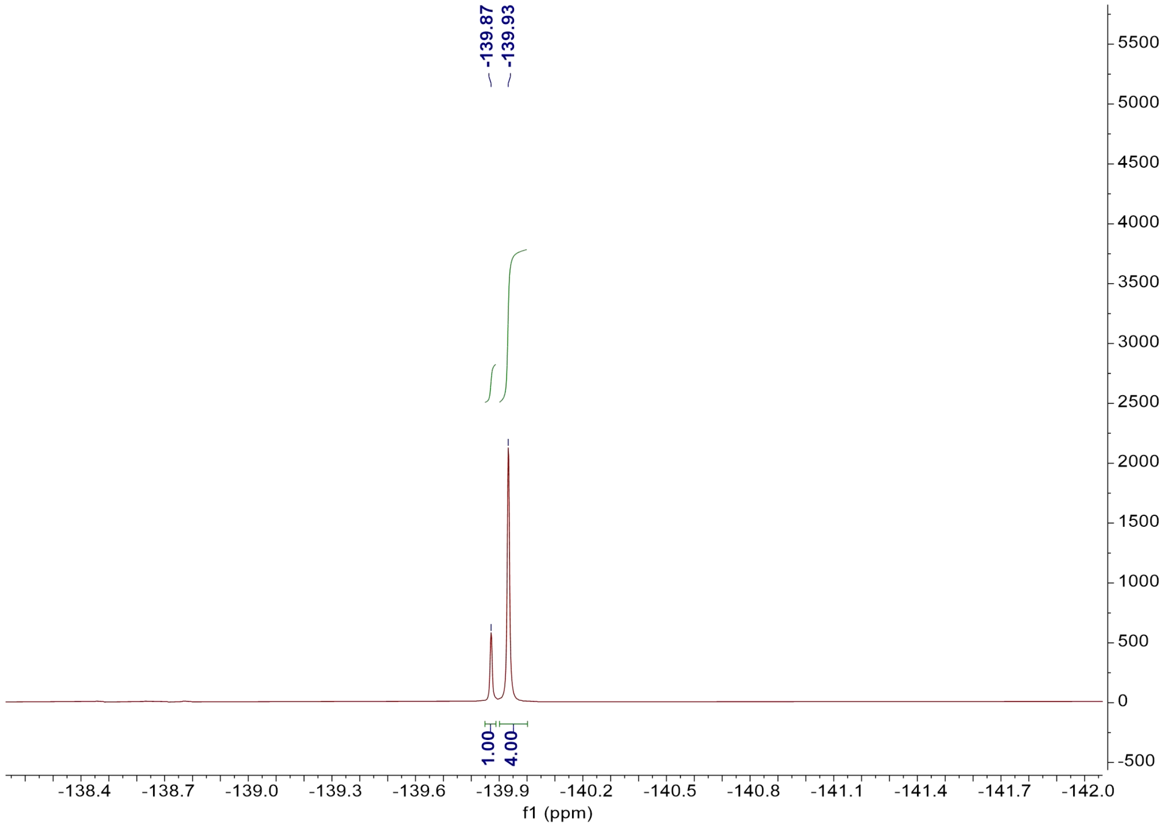

Figure S70. ^{19}F NMR spectrum of compound **6.**

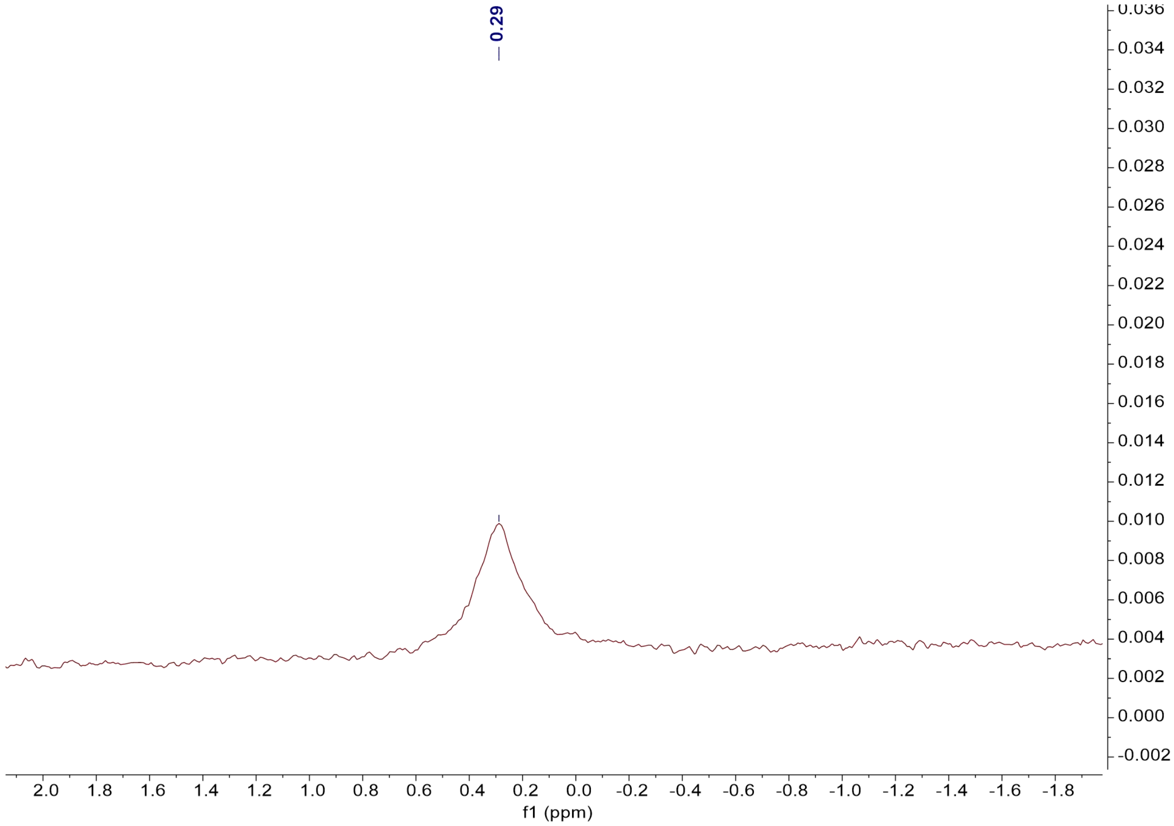

Figure S71. ^{11}B NMR spectrum of compound **6.**

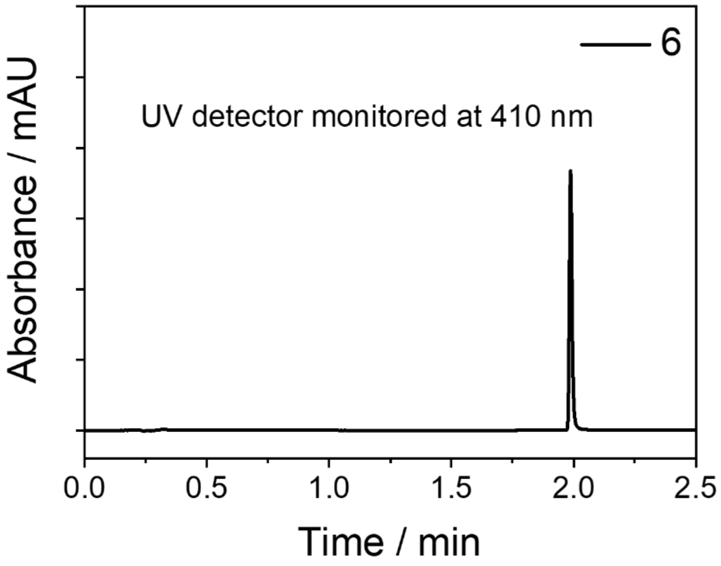

Figure S72. HPLC spectrum of compound **6** UV absorption monitored at 410 nm.

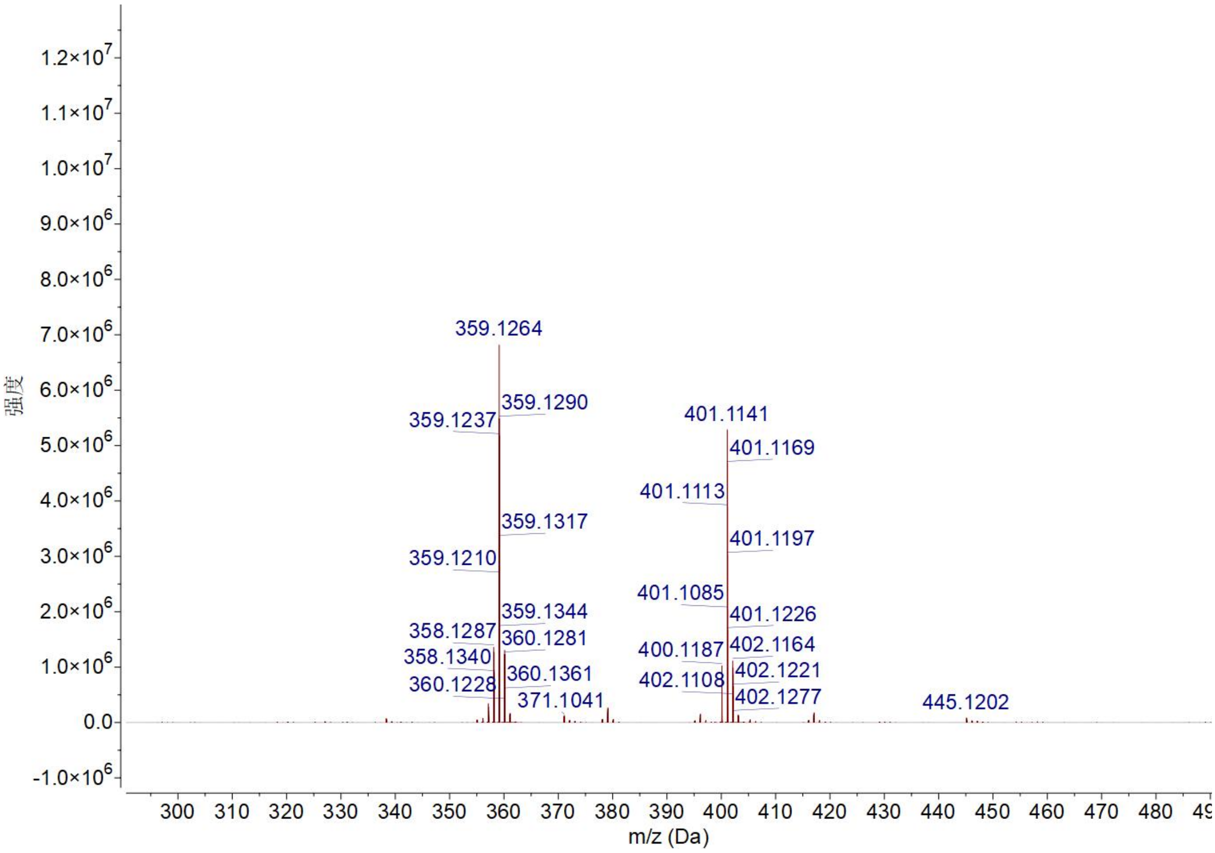

Figure S73. HRMS spectrum of compound **6**.

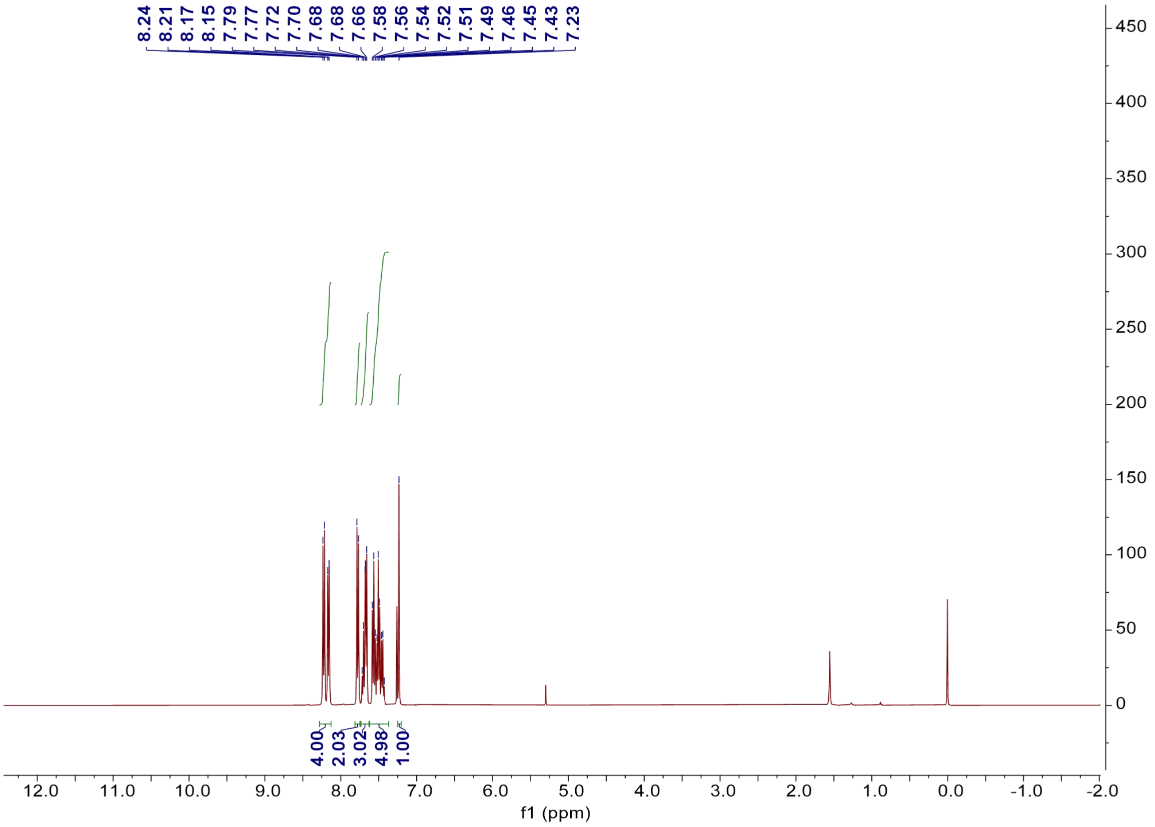

Figure S74. ^{1}H NMR spectrum of compound **7.**

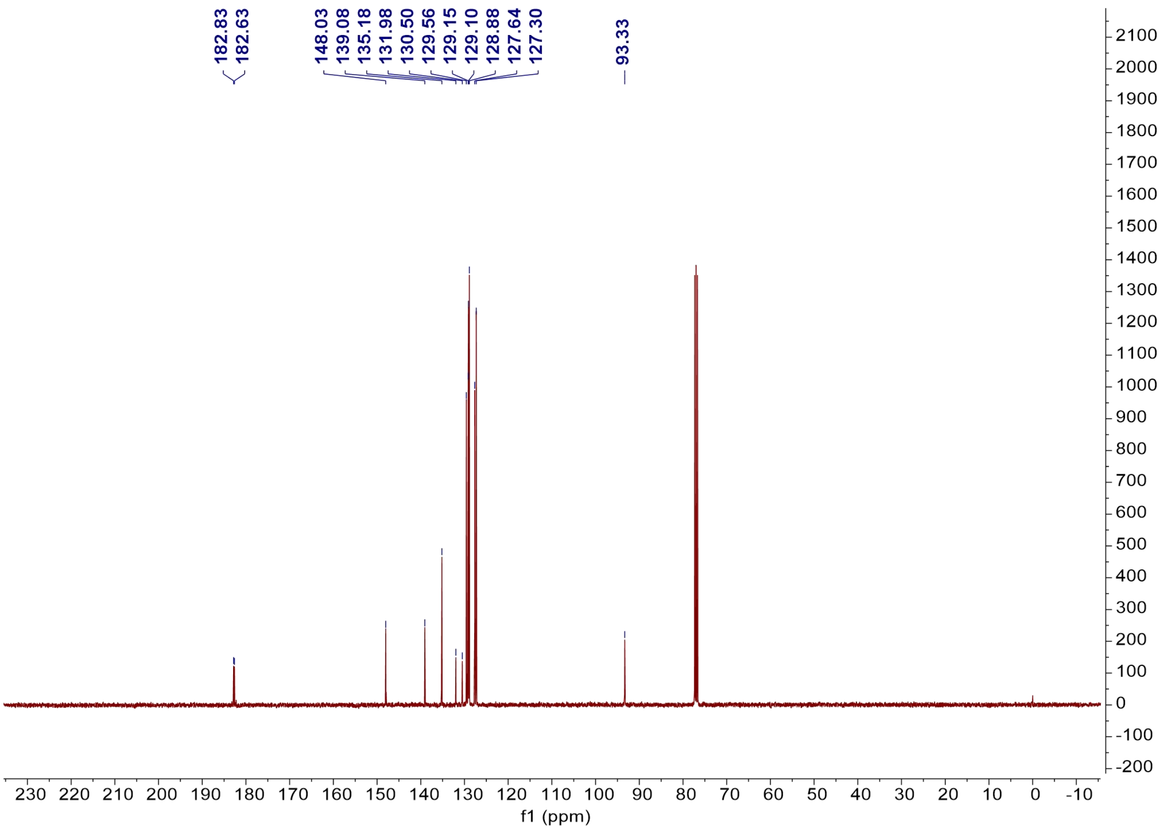

Figure S75. ^{13}C NMR spectrum of compound **7.**

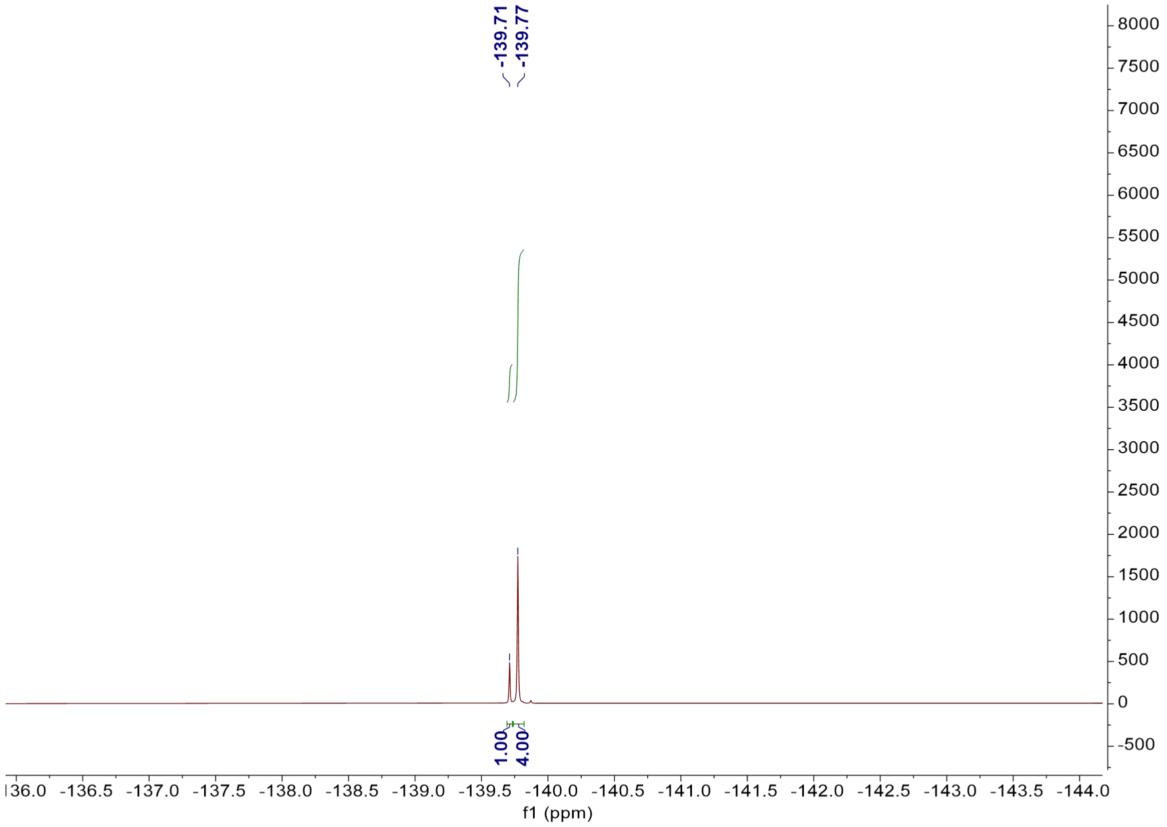

Figure S76. ^{19}F NMR spectrum of compound **7**.

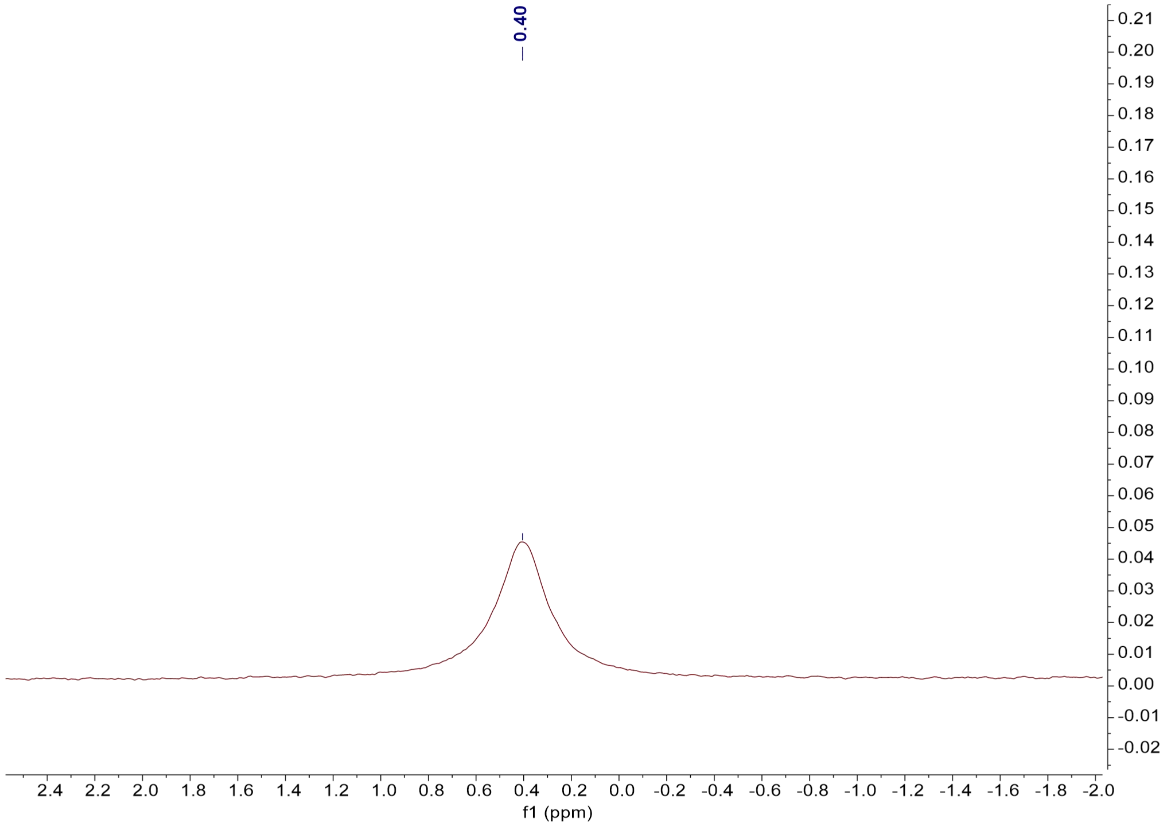

Figure S77. ^{11}B NMR spectrum of compound **7**.

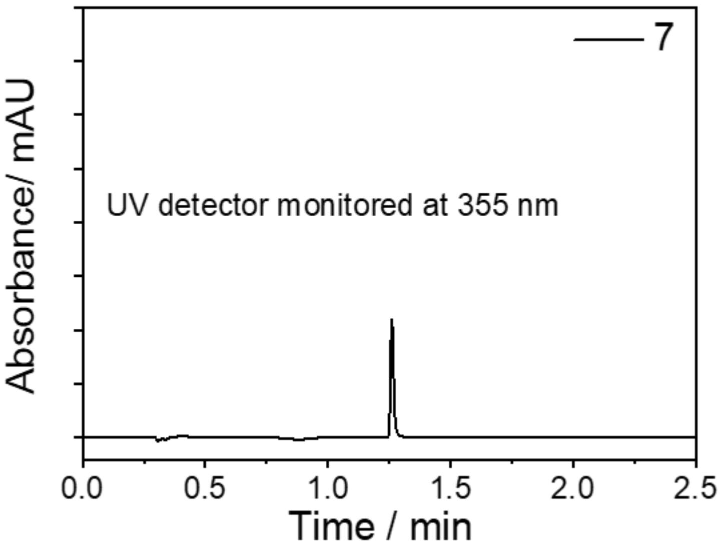

Figure S78. HPLC spectrum of compound **7** UV absorption monitored at 355 nm.

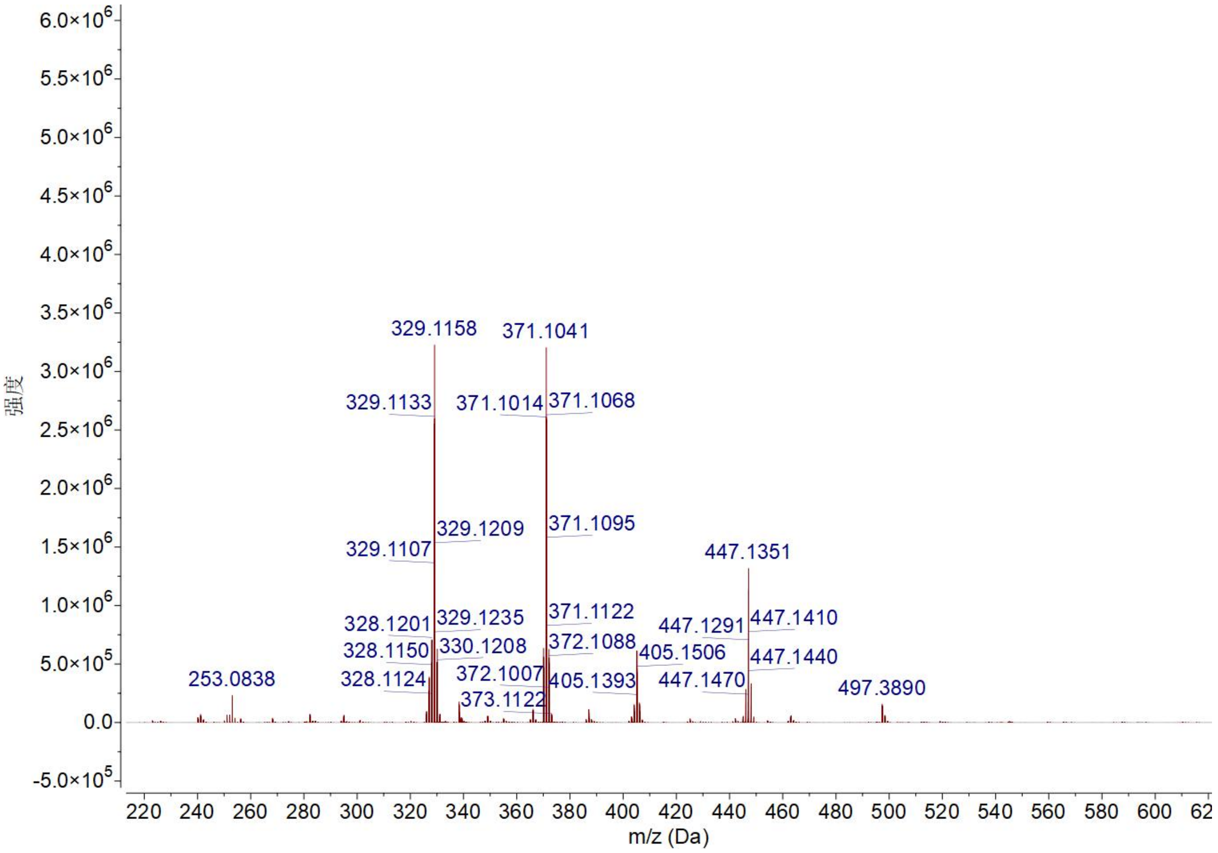

Figure S79. HRMS spectrum of compound **7**.

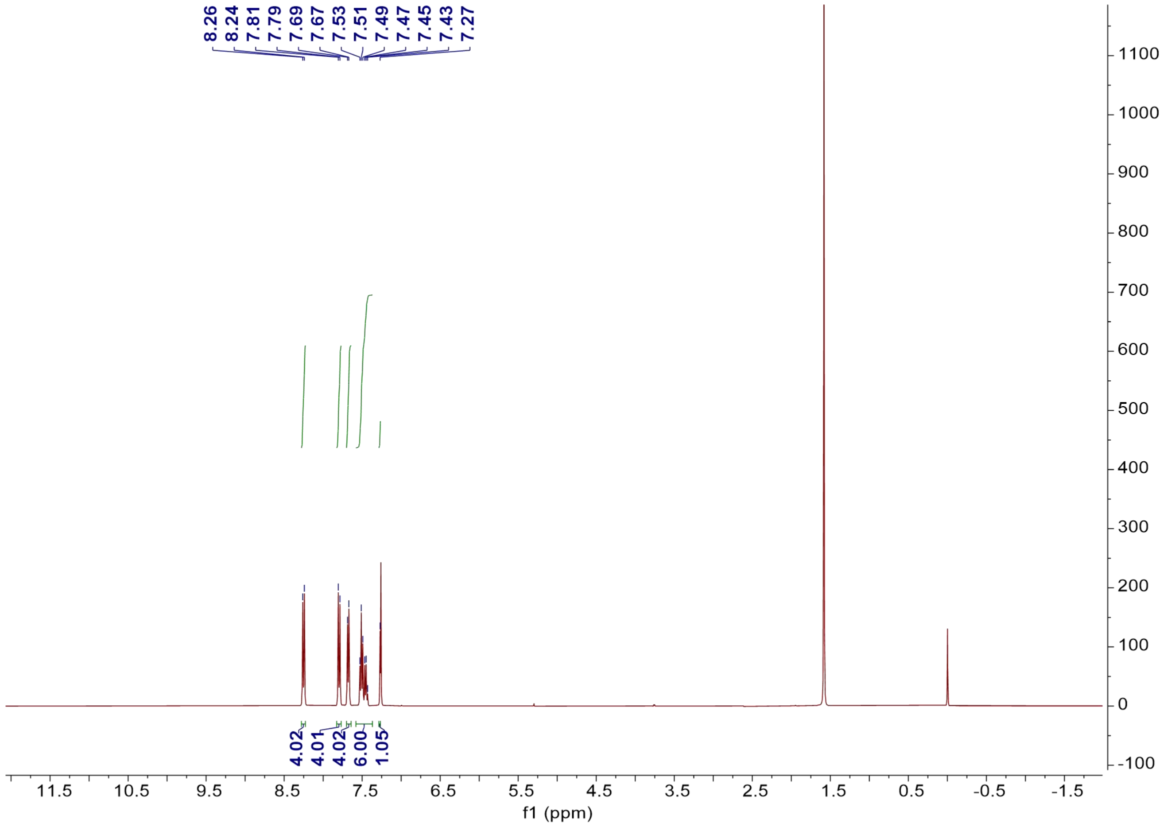

Figure S80. [11]H NMR spectrum of compound **8**.

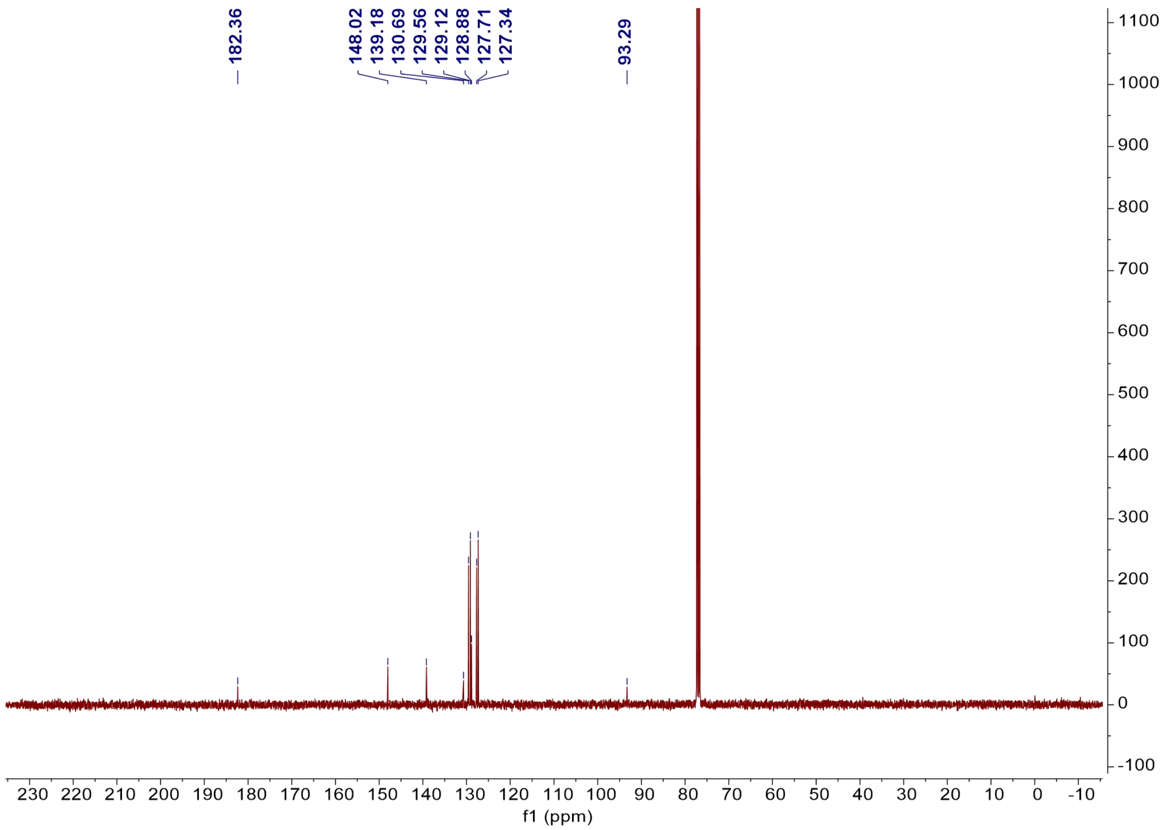

Figure S81. [11]C NMR spectrum of compound **8**.

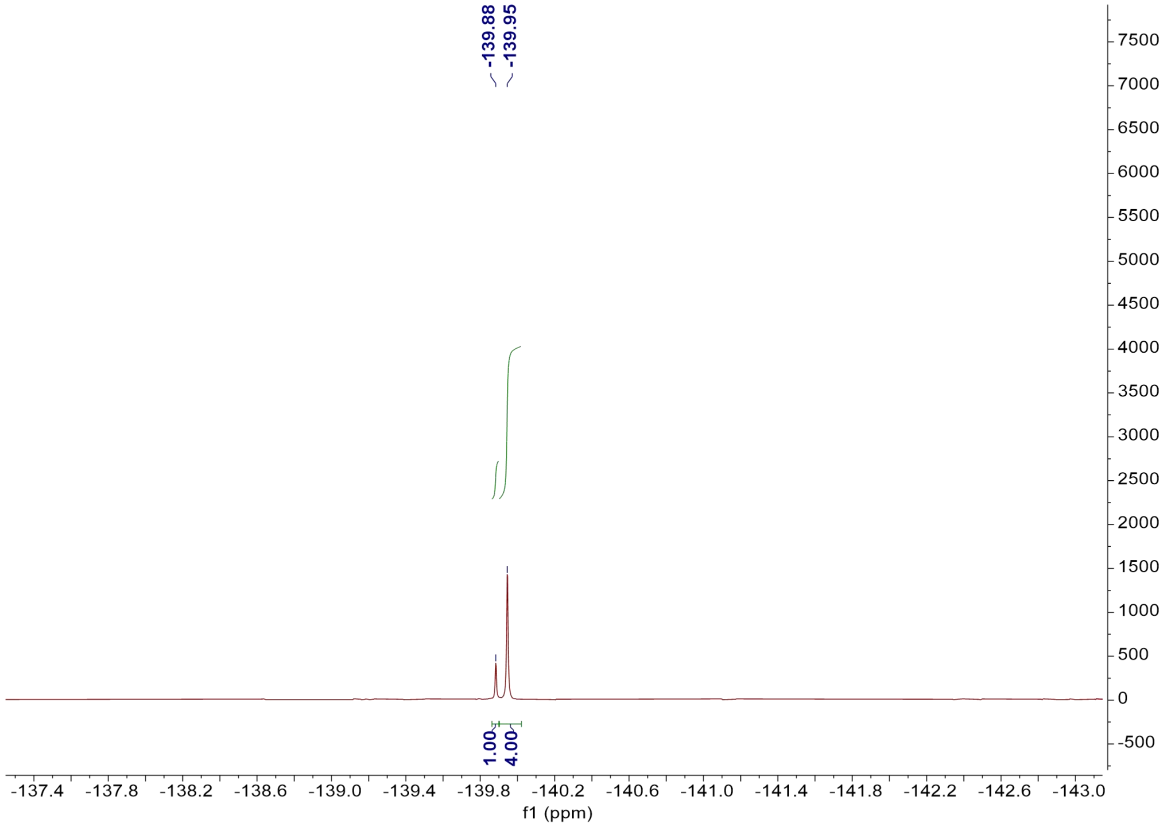

Figure S82. ^{19}F NMR spectrum of compound **8**.

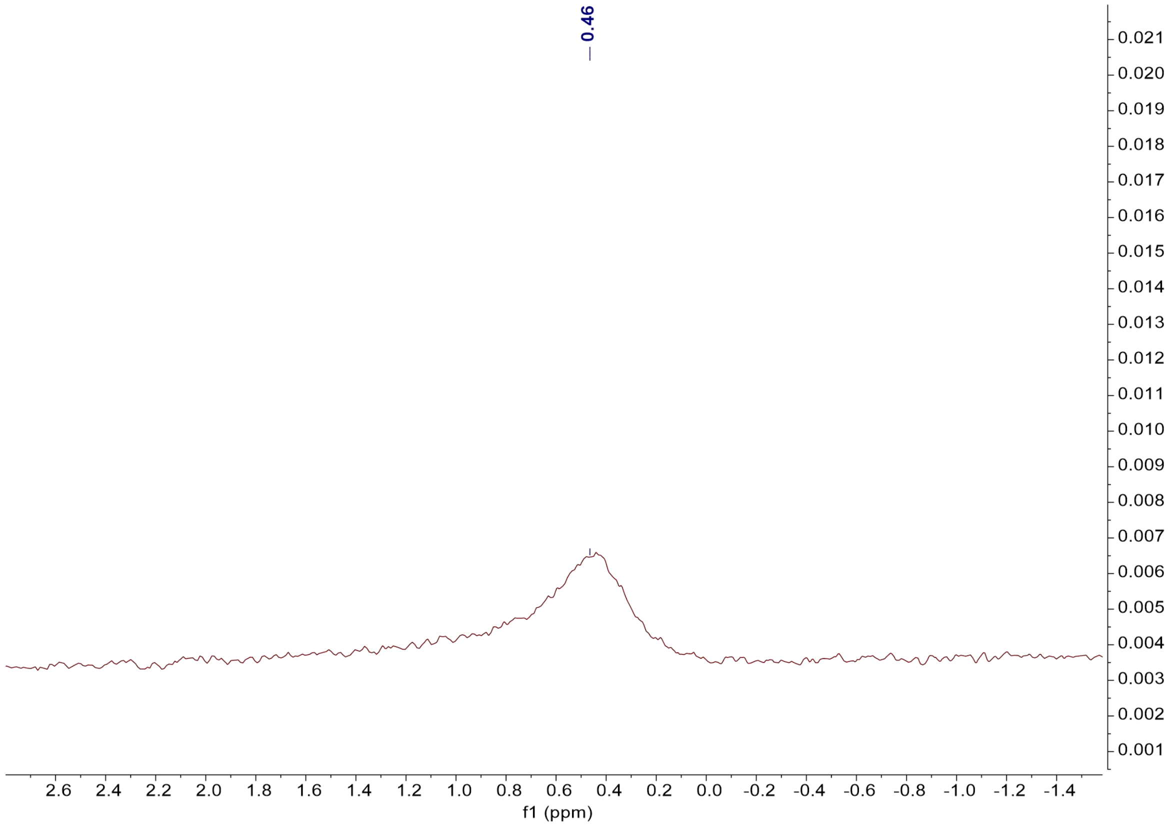

Figure S83. ^{11}B NMR spectrum of compound **8**.

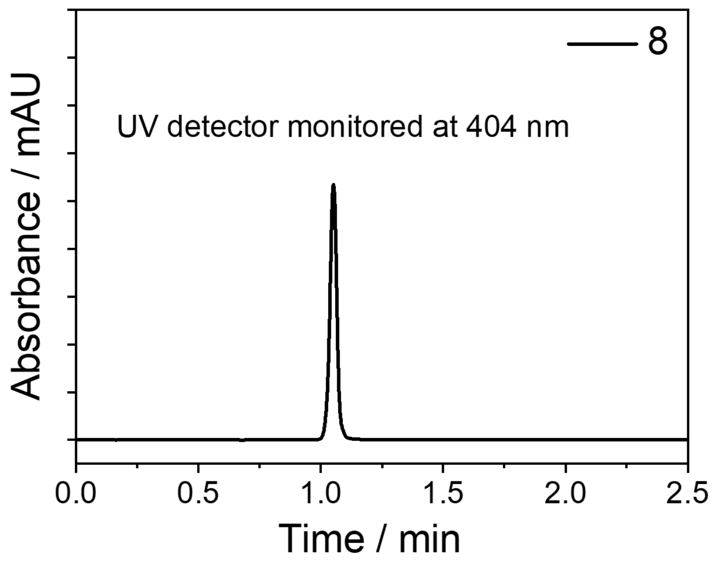

Figure S84. HPLC spectrum of compound **8** UV absorption monitored at 404 nm.

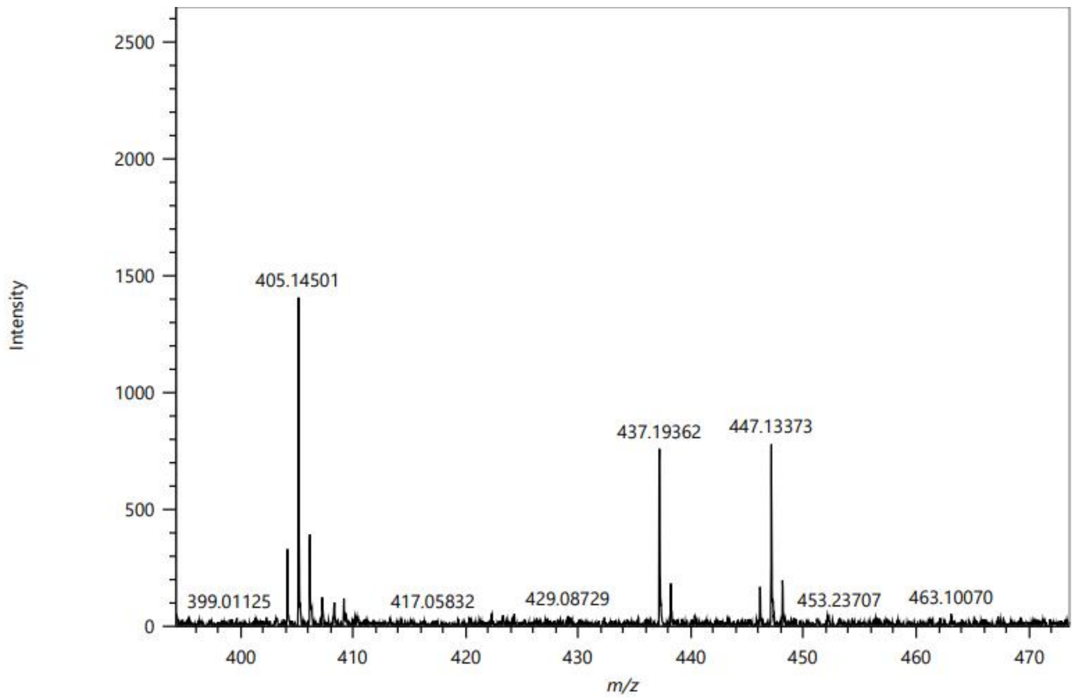

Figure S85. HRMS spectrum of compound **8**.
